# Hepatitis B and C testing strategies in healthcare and community settings in the EU/EEA: A systematic review

**DOI:** 10.1111/jvh.13182

**Published:** 2019-08-16

**Authors:** Lauren M. K. Mason, Irene K. Veldhuijzen, Erika Duffell, Ayla van Ahee, Eveline M. Bunge, Andrew J. Amato‐Gauci, Lara Tavoschi

**Affiliations:** ^1^ Pallas Health Research and Consultancy B.V. Rotterdam The Netherlands; ^2^ The Netherlands National Institute for Public Health and the Environment Bilthoven The Netherlands; ^3^ European Centre for Disease Prevention and Control Stockholm Sweden; ^4^ Present address: University of Pisa Pisa Italy

**Keywords:** Europe, hepatitis B, hepatitis C, screening, testing

## Abstract

An estimated 9 million individuals are chronically infected with hepatitis B virus (HBV) and hepatitis C virus (HCV) across the European Union/European Economic Area (EU/EEA), many of which are yet to be diagnosed. We performed a systematic review to identify interventions effective at improving testing offer and uptake in the EU/EEA. Original research articles published between 1 January 2008 and 1 September 2017 were retrieved from PubMed and EMBASE. Search strings combined terms for HBV/HCV, intervention, testing and geographic terms (EU/EEA). Out of 8331 records retrieved, 93 studies were selected. Included studies reported on testing initiatives in primary health care (9), hospital (12), other healthcare settings (31) and community settings (41). Testing initiatives targeted population groups such as migrants, drug users, prisoners, pregnant women and the general population. Testing targeted to populations at higher risk yielded high coverage rates in many settings. Implementation of novel testing approaches, including dried blood spot (DBS) testing, was associated with increased coverage in several settings including drug services, pharmacies and STI clinics. Community‐based testing services were effective in reaching populations at higher risk for infection, vulnerable and hard‐to‐reach populations. In conclusion, our review identified several successful testing approaches implemented in healthcare and community settings, including testing approaches targeting groups at higher risk, community‐based testing services and DBS testing. Combining a diverse set of testing opportunities within national testing strategies may lead to higher impact both in terms of testing coverage and in terms of reduction, on the undiagnosed fraction.

**Abbreviations**
DBS: dried blood spot
DBS: dried blood spot
GPs: general practitioners
HBV: hepatitis B virus
HCV: hepatitis C virus
MSM: men who have sex with men
OST: opioid substitution therapy
PLHIV: people living with HIV
PWID: people who inject drugs
RCT: randomized controlled trial
SIGN: Scottish Intercollegiate Guidelines Network
STI: sexually transmitted infection

## 1 INTRODUCTION

Across the European Union/European Economic Area (EU/EEA), an estimated 4.7 million people are chronically infected with hepatitis B virus (HBV) and 3.9 million are chronically infected with hepatitis C virus (HCV).^1^ HBV and HCV can both cause acute and chronic hepatitis, potentially leading to the development of cirrhosis, liver cancer or death of infected patients.^2^, ^3^ Early disease and development of liver damage are often asymptomatic,^4^, ^5^, ^6^ meaning that HBV and HCV infection may go undetected for many years^7^ and many infected people remain undiagnosed.^8^

Transmission of HBV and HCV can occur sexually, through blood‐to‐blood contact or vertically (mother‐to‐child). Over the past decades, there have been shifts in the patterns of transmission in Europe, due to various factors, including improvements in blood transfusion and healthcare safety standards, HBV vaccination programmes, harm reduction programmes targeting injecting drug use, as well as significant changes in patterns of injecting drug use and immigration. However, a number of population groups are still potentially at high risk or have a high burden of HBV/HCV in EU/EEA countries, including people who inject drugs (PWID), men who have sex with men (MSM), people living with HIV (PLHIV), people in prison and migrants from countries of high endemicity.^9^, ^10^, ^11^

As highly effective treatment options have become available for HBV and HCV,^12^, ^13^ it is crucial that an informed public health response is in place tailored to the local epidemiological situation, which will ensure that those infected are diagnosed and linked to care. Estimates of the undiagnosed fraction in the general population in EU/EEA countries range from 40% to 85% for HBV and 20% to 91% for HCV, with large variability between countries.^11^ The WHO has formulated an action plan to eliminate viral hepatitis as a public health threat in the European region by 2030, setting targets of 50% of people with chronic infection diagnosed by 2020 and 90% by 2030.^14^ To this end, testing programmes must be scaled up in order to reduce the undiagnosed fraction.

Testing can occur through a number of methods and in various settings, depending on the population group targeted and the local epidemiology and healthcare infrastructure. Testing in health care may take place across a range of different settings in primary health care and hospitals as well as other healthcare settings such as sexually transmitted infection (STI) clinics, antenatal services and pharmacies. Outside of formal healthcare facilities, settings within the community, such as homeless shelters, migrant services and community‐based drugs services can offer testing services which are adapted, targeted and made accessible to the populations that frequent them.^15^ Outreach testing, for example using mobile units, street outreach by community health workers, or satellite services based at other agencies can be used to reach people who are not in contact with other health services.^16^ Common testing strategies for hepatitis B and C include universal screening, birth cohort testing or testing targeted towards those at increased risk. In recent years, new technologies such as self‐testing kits, and strategies for testing implementation have been developed, which may be considered for incorporation in countries' national testing policies and programmes. Evidence around the effectiveness of different interventions in relation to uptake and positivity rates could help inform countries in deciding which strategic approaches to incorporate in national testing programmes.

A systematic review covering HBV and HCV testing studies in key populations in the European region until June 2013 found that, although a large number of studies on testing existed, these were unevenly distributed across Europe and that large gaps existed in certain key populations including migrants, people in prison and MSM.^17^ Previous systematic reviews on testing interventions focused solely on either HBV or HCV,^18^, ^19^, ^20^, ^21^, ^22^, ^23^ specific key populations or settings,^19^, ^21^, ^22^, ^24^ targeted testing interventions^18^, ^23^ or included comparative studies only.^25^ Finally, a comprehensive report on hepatitis testing policies and activities in EU/EEA countries revealed substantial gaps in testing coverage and testing offers targeting specifically higher risk groups across the region.^8^

The scope of this study was to provide an overview of different effective testing strategies for hepatitis B and C and their outcomes in the EU/EEA, covering all relevant population groups and settings. A systematic review was performed to collect, synthesize and analyse available data on HBV/HCV testing outcomes and acceptability measures from EU/EEA countries. This study was conducted as part of a larger project to develop an integrated European testing guidance for HBV, HCV and HIV, coordinated by the European Centre for Disease Prevention and Control (ECDC).

## 2 METHODS

### 2.1 Search strategy and selection criteria

Original research articles were retrieved from PubMed and EMBASE databases on 1 September 2017. PICO questions (Patient/problem, Intervention, Comparison and Outcome^26^) were formulated (listed in Supporting information). Search strategies combined controlled (MeSH/Emtree terms) and natural vocabulary on terms for HBV and HCV with terms for intervention and testing and geographic terms (EU/EEA) (detailed in Supporting Information). The search strategy employed also included terms for linkage to care, however, for the purposes of this article, methods and results applying to approaches to improve testing coverage are presented only. Only studies published between 1 January 2008 and 1 September 2017 were included in the search. Articles in all EU/EEA languages were included.

Only articles reporting data from EU/EEA countries were included. Publications were included which described approaches to improve coverage of testing and reported any of the following outcomes of interest: offer of test, uptake and coverage of testing, positivity rate, acceptability and feasibility outcomes. Studies on unlinked anonymous testing to determine prevalence and studies describing the sensitivity/specificity of laboratory tests were excluded. The full inclusion and exclusion criteria are listed in Table S3. Only original research articles (ie not reviews) and conference abstracts were included in this review; however, the reference lists of relevant systematic reviews retrieved in the literature search were checked manually for additional original articles not captured by the literature search. Conference abstracts from the International Liver Congress or retrieved in the systematic literature search, reporting relevant quantitative data and published since 2015, were included as grey literature. Additional publications captured were subject to the same inclusion and exclusion criteria listed above.

Two reviewers reviewed titles and abstracts of retrieved publications. Initially, a random sample of 5% was screened in duplicate; the results were then compared and used to refine the inclusion and exclusion criteria. Further rounds of duplicate review were conducted until a level of concordance of more than 95% was achieved, after which the remaining publications were divided between reviewers and screening continued in EndNote. The full texts of selected articles were subsequently screened by two reviewers, of which a random sample of 20% was screened in duplicate and these reached more than 95% concordance. The remaining 80% of publications were divided between the reviewers and screened. Articles were included in case of uncertainty about inclusion or exclusion if this was not resolved after discussion between the reviewers.

### 2.2 Definitions

Primary health care was defined as health care provided by general practitioners (GPs). Hospital settings included all hospital departments including inpatients, outpatients, medical admissions units and infectious disease units. Other healthcare settings included any formal healthcare settings outside of primary health care or hospital departments, for example STI clinics, pharmacies and prisons. Community‐based testing was defined as any programme or service offering HBV/HCV testing outside of formal health facilities. Drugs services were defined as services offering prevention, support, detox and treatment for addiction to drugs or alcohol, either embedded in healthcare settings or set in the community. Outreach activities were defined as testing activities taking place in the community without a fixed‐site facility, including mobile units or vans, street outreach by community health workers and regular satellite services based at other agencies.

Testing offer rate was defined as the proportion of people targeted by a testing programme who were offered testing, while testing coverage was defined as the percentage of people targeted by a testing programme that received testing. Positivity rate was defined as the percentage of people testing positive for HBsAg and anti‐HCV for HBV and HCV, respectively. When other markers were reported instead, these data were extracted, and the marker was specified in tables.

### 2.3 Data extraction and quality assessment

Relevant data were extracted from included articles and recorded in a data extraction file in Microsoft Excel. A predefined set of variables covering study characteristics, study population details and outcomes was extracted per study. The complete list of variables is provided in Table S5. The unit for data extraction was study, not article. A study is defined as a report of data on a testing approach or linkage to care approach for HBV or HCV, in a defined country, over a discrete period of time. Therefore, one article may contain more than one study. If a study was captured by two different articles, the study was extracted once and the article with the most detail used as a reference.

The quality of included peer‐reviewed literature was assessed using checklists developed by the Scottish Intercollegiate Guidelines Network (SIGN)^27^ that include the most important criteria on publication quality from the PRISMA and STROBE guidelines. Using these checklists, an overall quality score was assigned per study: high (++), acceptable (+) and low (−). As some of the included publications concerned study designs for which no checklists exist, a list (provided in Table S6) was compiled of relevant aspects from standard checklists, regarding the level of detail and clarity of the study, appropriateness of study population, data collection and denominator, and the representativeness of the sample, which were answered with yes or no per study. Using the checklist, it was not possible to calculate an overall quality score for these studies; therefore, all relevant articles were included regardless of their quality, but the results of the assessment were taken into consideration in interpreting the results. Articles were excluded, however, when the methods and/or results provided an insufficient level of details making it not possible to accurately extract data. No quality assessment was performed on included grey literature publications.

A set of detailed summary tables were developed per setting (provided in Tables S7‐S11), containing the following information: study reference, country, study period, study design, study population and specific setting, sample size, outcomes (test offer, coverage and positivity rate, acceptance rate and patient and provider indicators of acceptability and feasibility), critical appraisal and comments. Within each table, findings are ordered by virus, study population, country and year of publication.

## 3 RESULTS

**Figure 1:**
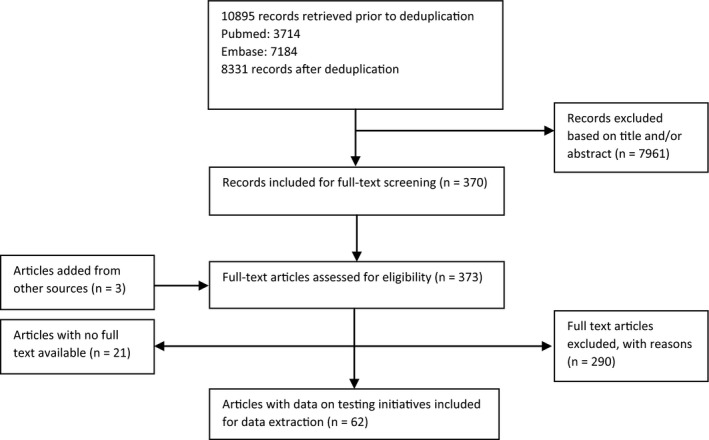
PRISMA flow diagram for the systematic review

The literature search retrieved 8331 unique publications, of which 370 were selected based on title and abstract and were assessed in full text for eligibility. Of these, 62 articles were retrieved that formed the evidence base for the effectiveness of testing initiatives and interventions (Figure 1). Reasons for exclusion of publications are listed in Table S4. The included publications comprised 93 studies in total, each detailing an intervention designed to improve coverage of HBV or HCV testing in a certain setting. A total of 78 studies were from peer‐reviewed publications and 15 concerned conference abstracts. A formal quality assessment was performed for 19 peer‐reviewed studies; the remainder had study designs which precluded this. Detailed information of each included study is provided in Tables S7‐S11.

### 3.1 Testing initiatives in primary healthcare settings

Nine studies that reported outcomes on testing initiatives performed in primary healthcare settings were retrieved^28^, ^29^, ^30^, ^31^, ^32^, ^33^, ^34^, ^35^, ^36^ (Table 1). HBV/HCV test offer rates, coverage and positivity rates, where reported, ranged widely between studies. The highest offer rate and coverage reported was 70% and 100%, respectively, in a study targeting migrants.^30^ Very high positivity rates for HCV were reported in initiatives targeting PWID (70%) and homeless people (26%).^29^, ^33^ Two initiatives targeting migrants reported HBV and HCV positivity rates of 0%.^30^, ^32^

**Table 1:** Evidence base for the effectiveness of testing initiatives in primary healthcare settings

Intervention	Studies included Quality of evidence	Target population	Test offer	Coverage/number of tests/tested^b^	Positivity rate
Risk group testing	**HBV**				
	N = 3 studies^30^, ^32^, ^36^ Quality: 3 studies NA	Migrants (n = 3) (sample size: 47‐560^a^)	100%	Number tested/tests: 2223 Coverage: 2.3% and 70%	0%‐6.7%
	**HCV**				
	N = 4 studies^29^, ^30^, ^32^, ^33^ Quality: 1 study low 3 studies NA	PWID (n = 1) (sample size: intervention 485, control NR)	52% intervention	Coverage: 24.8% intervention, 0.3% control	Comparative study: 70% intervention, 22% control
		Homeless (n = 1) (sample size: NR)		Number tested/tests: 460	26% 9.6% newly diagnosed
		Migrants (n = 2) (sample size: 47 and 560)	100%	Coverage: 2.3% and 70%	0%
Birth cohort testing	**HCV**				
	N = 1 study^28^ Quality: Acceptable	30‐ to 54‐year‐olds (n = 1) (sample size: Intervention 584, control NR)	Comparative study: 72% intervention; 0% control	Comparative study: coverage: 20% intervention; 0% control	Comparative study: 13% intervention; NA control
Novel testing	**HCV**				
	N = 2 studies^33^, ^35^ Quality: 2 studies NA	General pop (n = 1) (sample size: 600‐29 600)		Coverage (oral): 9.1%‐22%	0.4%‐4.5%
		Homeless (n = 1) (sample size: NR)		Number tested/tests (oral): 460	26% 9.6% newly diagnosed
Education	**HBV**				
	N = 1 study^30^ Quality: NA	Targeted at GPs Migrants (n = 1) (sample size: 47)	100%	Coverage: 70%	0%
	**HCV**				
	N = 2 studies^30^, ^31^ Quality: 1 study low 1 study NA	Targeted at GPs Migrants (n = 1); general pop. (n = 1) (sample size: 47^a^)	100%	Coverage: 70% Comparative study: number tested: campaign + education (intervention): 57 tests before; 172 during; campaign (control): 86 tests before; 118 during OR of the increase intervention vs control: 2.2 (95% CI 1.5‐3.3)	0% Comparative study: campaign + education (intervention): 0% before; 1.7% during; campaign (control): 1.7% before; 0.8% during
Campaign	**HCV**				
	N = 2 studies 31, 34 Quality: 1 study low 1 study NA	Targeted at public General pop. (n = 1) (no sample)		Comparative study: number tested/tests: campaign + education (intervention): 57 tests before; 172 during; campaign (control): 86 tests before; 118 during OR of the increase intervention vs control: 2.2 (95% CI 1.5‐3.3)	Comparative study HCV: campaign + education (intervention): 0% before; 1.7% during; campaign (control): 1.7% before; 0.8% during
		Targeted at GPs/risk groups General pop. (n = 1) (no sample)		Comparative study: number HCV tested/tests: before 5421 tests; during 10 117 tests	Comparative study HCV: before 9.6%; during 6.8%

Abbreviations: HBV: hepatitis B virus; HCV: hepatitis C virus; HIV: human immunodeficiency virus; NA: not applicable; NR: not reported; oral: oral sampling; PWID: people who inject drugs; TB: tuberculosis

a Denominator not reported for all studies.

b Number of tests is only reported here if no data on coverage are available.

One comparative study on HCV risk group testing in PWID reported testing coverage of 24.8% among PWID in GP practices exposed to the intervention, compared to 0.3% of PWID tested in comparable control practices.^29^ In a comparative study on birth cohort testing for HCV testing in an area of high prevalence of HCV and intravenous drug use, 72% of 30‐ to 54‐year‐olds were offered testing, compared to 0% in a control practice in the same area where birth cohort testing was not implemented.^28^ In an additional comparative study, the number of tests performed increased from 5421 before to 10 117 during a national testing programme which involved awareness‐raising activities for GPs and those at higher risk.^34^ Another comparative study found a significant increase in the number tested when a public awareness campaign was combined with additional educational brochures and training for GPs, which resulted in three times more people being tested, compared to a control group in which only the campaign was implemented and testing rates increased by 1.4 times.^31^

Two studies provided data on the acceptability and feasibility of testing in primary healthcare settings. Testing acceptance rates were 56% among PWID^29^ and 70% among migrants.^30^ Among PWID interviewed about a testing intervention, all responded positively about the acceptability of provider‐initiated HCV testing. Staff viewed the intervention as an opportunity to facilitate identification and subsequent referral.^29^

### 3.2 Testing initiatives in hospital settings

Twelve studies were identified on the effectiveness of testing initiatives and interventions in hospital settings^34^, ^35^, ^37^, ^38^, ^39^, ^40^, ^41^ (Table 2). Test offer rates, coverage and HBV/HCV positivity rates varied with high offer rates (83% and 100%) in testing initiatives targeted at migrants and psychiatric patients.^37^, ^40^, ^41^ Coverage was highest (88.4%) in a study reporting on a universal testing initiative conducted at a single emergency department.^38^ A separate universal testing initiative conducted in multiple emergency departments, however, yielded a much lower overall coverage of 27%, although with variations among testing sites.^39^ Positivity rates for HBV and HCV were higher in studies reporting on initiatives targeted at key populations (2.2%‐7.8% for HBV and 0.3%‐8.7% for HCV) than those aimed at the general population.^37^, ^40^, ^41^

**Table 2:** Evidence base for the effectiveness of testing initiatives in hospital settings

Intervention	Studies included Quality of evidence	Setting; target population	Test offer	Coverage/number of tests/tested^a^	Positivity rate
Risk group testing	**HBV**				
	N = 3 studies^37^, ^40^, ^41^ Quality: 3 studies NA	Other hospital departments (n = 3) (sample size: 105‐3226) Migrants (n = 2); psychiatric patients (n = 1)	Migrants: 100% Psychiatric patients: 83%	Coverage migrants: 28.7% and 61% Coverage psychiatric patients: 54%	Migrants: 2.2% and 7.8% Psychiatric patients: 7.0%
	**HCV**				
	N = 3 studies^37^, ^40^, ^41^ Quality: 3 studies NA	Other hospital departments (n = 3) (sample size: 105‐3226) Migrants (n = 2); psychiatric patients (n = 1)	Migrants: 100% Psychiatric patients: 83%	Coverage migrants: 28.7% and 61% Coverage psychiatric patients: 54%	Migrants: 0.3% and 3.6% Psychiatric patients: 8.7%
Universal testing	**HBV**				
	N = 2 studies^38^, ^39^ Quality: 2 studies NA	ED only (n = 2) (sample size: 7807 and 10 000) General population (n = 2)		Coverage: 27% and 88.4%	0.5% and 0.7% 0.2% and 0.5% newly diagnosed
	**HCV**				
	N = 2 studies^38^, ^39^ Quality: 2 studies NA	ED only (n = 2) (sample size: 7807 and 10 000) General population (n = 2)		Coverage: 27% and 88.4%	1.8% and 5% 0.6% and 0.7% newly diagnosed
Novel testing	**HCV**				
	N = 1 study^35^ Quality: NA	Other hospital departments (n = 1 (sample size: 600‐29 600) General population (n = 1)		Comparative study: coverage (oral): 9.1% (2011) 14% (2012) 16.9% (2013) 22% (2014)	Comparative study: 0.5% (2011) 0.5% (2011) 0.4% (2013) 4.5% (2014)
Education	**HBV**				
	N = 1 study^40^ Quality: NA	Targeted at migrants Migrants (n = 1) (sample size: 3226)	100%	Coverage: 28.7%	2.2%
	**HCV**				
	N = 1 study^40^ Quality: NA	Targeted at migrants Migrants (n = 1) (sample size: 3226)	100%	Coverage: 28.7%	0.3%
Campaign	**HBV**				
	N = 2 studies^39^, ^40^ Quality: 3 studies NA	Targeted at patients General pop. (n = 1) (sample size: 7807)		Coverage: 27%	0.7% 0.5% newly diagnosed
		Targeted at‐risk groups Migrants (n = 1) (sample size: 3226)	100%	Coverage: 28.7%	2.2%
	**HCV**				
	N = 3 studies^34^, ^39^, ^40^ Quality: 3 studies NA	Targeted at patients General pop. (n = 1) (sample size: 7807)		Coverage: 27%	1.8% 0.7% newly diagnosed
		Targeted at‐risk groups General pop. (n = 1) (sample size: NR) Migrants (n = 1) (sample size: 3226)	100%	Comparative study: before 10 536 tests; during 12 170 Coverage: 28.7%	Comparative study: 9.9% before; 7.9% during 0.3%

Abbreviations: ED, emergency department; HBV, hepatitis B virus; HCV, hepatitis C virus; HIV, human immunodeficiency virus; NA, not applicable; NR, not reported; oral, oral sampling.

a Number of tests is only reported here if no data on coverage are available.

One comparative study reported on implementation of oral sampling for HCV testing between 2011 and 2014.^35^ Over this period, coverage increased from 9.1% to 22% and positivity rates increased from 0.5% to 4.5%. In another comparative study, a national programme involving awareness‐raising activities was associated with an increase in number of tests taken in hospitals from 10 536 tests before and 12 170 during the intervention.^34^

### 3.3 Testing initiatives in other healthcare settings

Thirty‐one studies were included relating to other healthcare settings^34^, ^35^, ^42^, ^43^, ^44^, ^45^, ^46^, ^47^, ^48^, ^49^, ^50^, ^51^, ^52^, ^53^, ^54^, ^55^, ^56^, ^57^, ^58^, ^59^, ^60^, ^61^, ^62^, ^63^, ^64^ (Table 3), which included antenatal services, clinics for people with no health insurance, drug services (embedded in health services), migrant clinics, pharmacies, prisons, public health clinics and STI clinics. Testing coverage during or after interventions were found to vary widely between and within settings, with the highest coverage levels reported by studies in Italian migrant clinics (87%‐91.4%),^48^, ^54^ clinics for people with no health insurance (71%‐98.2%)^44^, ^57^ and public health clinics (90% and 98%).^51^ Four studies on novel testing initiatives yielded high coverage levels when dried blood spot (DBS) sampling or rapid tests were used (up to 98.2% for rapid testing and up to 96.6% for DBS).^44^, ^50^, ^64^ The highest positivity rates for both diseases were reported in studies targeting drug users or PWID (up to 48% anti‐HBc positive for HBV and up to 61% for HCV).^43^, ^51^, ^52^, ^59^, ^62^, ^64^ No studies conducted in other healthcare settings reported test offer rates.

**Table 3:** Evidence base for the effectiveness of testing initiatives in other health care settings

Intervention	Studies included Quality of evidence	Setting/target population	Coverage/number of tests/tested^c^	Positivity rate
Risk group testing	**HBV**			
	N = 6 studies^48^, ^54^, ^56^, ^57^, ^61^, ^62^ Quality: 6 studies NA	Migrant clinic (n = 2) (sample size: 516 and 4078)	Coverage: 87% and 91.4%	6.0% and 7.7%
		Prisons (n = 2) (sample size: 3468^a^)	Number tested: 160 Coverage: 65.3%	0% and 4.4% 1.5% newly diagnosed
		Drug services (n = 1) (sample size: 287 and 2024)	Coverage: 34% and 69%	33% and 48% (anti‐HBc)
		Clinic for people with no health insurance (n = 1) (sample size: NR)	Coverage: 90% when screening is proposed during a prevention interview; 71% without interview	6.9%
	**HCV**			
	N = 15 studies^43^, ^45^, ^47^, ^48^, ^49^, ^51^, ^52^, ^56^, ^57^, ^58^, ^59^, ^61^, ^62^, ^63^, ^64^ Quality: 3 studies acceptable 1 study low 11 studies NA	STI clinics (n = 1) (sample size: 3365) MSM (n = 1)	Coverage: 69%	0.65%
		Prisons (n = 3) (sample size: 3468 ‐ 3600^a^)	Number tested: 160 Coverage: 64.6% Comparative study (DBS): ORs for effect of the intervention on testing rate: OR: 0.86; 95% CI: 0.71‐1.06; *P* = 0.153	22.8% and 33.8% 1.5% newly diagnosed
		Drug services (n = 3) (sample size: 287‐2566)	Coverage: 53%‐84.2%	26%‐61%
		Antenatal services (n = 1) (sample size: 4369)	28.3% received targeted screening	Comparative study: 1.3% (compared to 1.7% universal screening; difference between the two NS)
		Migrant clinic (n = 1) (sample size: 4078)	Coverage: 90.8%	3.6%
		Pharmacies (n = 2) (sample size: 143 intervention, 561 control; 244 conventional care pathway; 262 pharmacist care pathway)	Comparative study (DBS): 30% intervention; 13% control. OR: 2.25 (95% CI 1.48‐3.42) Comparative study: 24% coverage conventional care pathway; 36% pharmacist care pathway	Comparative study: 25.9% conventional care pathway 26.5%; pharmacist care pathway
		Drug clinics and prisons (n = 1) (sample size: 6550 intervention, 5800 control)	Comparative study coverage (DBS) intervention: 8.4% before, 20.6% during intervention control: 7.7% before, 5.4% during intervention	32% (overall)
		Public health clinic (n = 1) (sample size: 81 and 497)	Coverage: 90% and 98%	60% (overall)
		Specialist services^b^ (n = 1) (sample size: 1322)		55%
		Clinic for people with no health insurance (n = 1) (sample size: NR)	Number tested/tests: 1196 Coverage: 90% when screening is proposed during a prevention interview; 71% without interview	5.8%
Universal testing	**HCV**			
	N = 1 study^47^ Quality: NA	Antenatal services (n = 1) (No sample size)	Number tested: 4222	Comparative study: 1.7% (compared to 1.3% targeted screening; difference between the two NS)
Novel testing	**HBV**			
	N = 3 studies^44^, ^50^, ^56^ Quality: 1 study high 1 study acceptable 1 study NA	Clinic for people with no health insurance (n = 1) (sample size: 162 intervention, 162 control)	Coverage: 64.2% (serology); 98.2% (RT)	Comparative study: 9.6% (serology); 8.1% (RT)
		Antenatal services (n = 1) (Sample size: 41 pre‐intervention; 58 post‐intervention; 91 pre‐control; 68 post‐control)	Comparative study: Coverage (DBS): 62% pre‐intervention; 97% post‐intervention; 40% pre‐control; 39% post‐control	Comparative study: 0%‐3% pre‐intervention; 3%‐22% post‐intervention; 6%‐8% pre‐control; 2%‐9% post‐control
		Prisons (n = 1) (sample size: NR)	Number tested (DBS): 160	0%
	**HCV**			
	N = 11 studies^35^, ^42^, ^43^, ^44^, ^45^, ^49^, ^52^, ^56^, ^58^, ^59^, ^64^ Quality: 1 study high 4 studies acceptable 1 study low 5 studies NA	Clinic for people with no health insurance (n = 1) (sample size: 162 intervention, 162 control)	Coverage: 64.2% (serology); 98.2% (RT)	Comparative study: 3.8% (serology); 2.5% (RT)
		Drug services (n = 3) (sample size: 25‐1123)	Number tested/tests (DBS): 266 Coverage (FibroScan): 20% Coverage (DBS): 84.2%	31.2% and 35%
		Prisons (n = 2) (sample size: 3600^a^)	Number tested (DBS): 160 Comparative study (DBS): ORs for effect of the intervention on testing rate: 0.86; 95% CI: 0.71‐1.06; *P* = 0.153	33.8%
		Drug clinics and prisons (n = 1) (sample size: 6550 intervention, 5800 control)	Comparative study coverage (DBS) intervention: 8.4% before, 20.6% during intervention control: 7.7% before, 5.4% during intervention	32% (overall)
		STI clinics and GP practices (n = 1) (sample size: 29 600)	Coverage (oral): 15.2%	0.6%
		Specialist services^b^ (n = 1) (sample size: 1322)		55%
		Pharmacies (n = 2) (sample size: 143 intervention, 561 control; 244 conventional care pathway; 262 pharmacist care pathway)	Comparative study coverage (DBS): 30% intervention; 13% control. OR: 2.25 (95% CI 1.48‐3.42) Comparative study coverage (DBS): 24% conventional care pathway; 36% pharmacist care pathway	Comparative study: 25.9% conventional care pathway; 26.5% pharmacist care pathway
Education	**HCV**			
	N = 1 study^42^ Quality: High	Targeted at‐risk groups Drug services (n = 1) (sample size: 52)	Comparative study coverage: 7% control; 20% intervention (NS) Willingness for HCV screening: Control: 89%; 56%; 67% (baseline; after 1 mo; after 3 mo) Intervention: 86%; 96%; 100%; 77%; 100% (baseline; after info; after 1 mo; after 3 mo; after FibroScan)	
Campaign	**HCV**			
	N = 2 studies^34^, ^46^ Quality: 2 studies NA	Targeted at general pop. Mixed settings (n = 1), STI clinic/Prison (n = 1) (sample size: 4200 and 33 667^a^)	Coverage from mixed settings: 2.3%‐3.7%	Comparative study: prison: 44.4% before, 27.0% during intervention STI clinic: 5.2% before, 3.5% during intervention 45%‐53% newly diagnosed
Guideline	**HCV**			
	N = 1 study^53^ Quality: NA	STI clinics (n = 1) (sample size: NR)	Comparative study: Coverage: 4.7% before, 13.6% after intervention	
Clinical decision‐making tools Computer‐assisted self/ personal interviewing (CASI/CAPI) vs paper & pen (PAPI, control)	**HBV**			
	N = 1 study^60^ Quality: High	STI clinic (n = 1) (sample size: 2318)	Comparative study: Coverage: PAPI: 16%; CAPI: 24%; CASI: 17%	Comparative study: Any STI: PAPI: 10%; CAPI: 11%; CASI: 10%
	**HCV**			
	N = 1 study^60^ Quality: High	STI clinic (n = 1) (sample size: 2318)	Comparative study: Coverage: PAPI: 3%; CAPI: 9%; CASI: 3%	Comparative study: Any STI: PAPI: 10%; CAPI: 11%; CASI: 10%

Abbreviations: BBV, bloodborne virus; CASI, computer‐assisted self‐interviewing; CAPI, computer‐assisted personal interviewing; CI, confidence interval; DBS, dried blood spot; GP, general practitioner; HBV, hepatitis B virus; HCV, hepatitis C virus; HIV, human immunodeficiency virus; mo, months; MSM, men who have sex men; NA, not applicable; NS, not significant; OR, odds ratio; PAPI, pen‐and‐paper interviewing; RT, rapid test; STI, sexually transmitted infection.

a Denominator not reported for all studies.

b specialist services mainly targeting PWID.

c Number of tests is only reported here if no data on coverage are available.

Eleven comparative studies reported on interventions in other healthcare settings. In pharmacies, HCV testing coverage was 30% among drug users receiving opioid substitution therapy (OST) when DBS testing was offered, compared to 13% in pharmacies which did not offer DBS. Furthermore, DBS testing coverage was 36% in pharmacies in which pharmacist‐led care pathways were used compared to 24% when conventional care pathways were used.^58^, ^59^ One randomized controlled trial (RCT) found that offering DBS testing for HCV in drug clinics and prisons significantly increased coverage rates in intervention sites with a 14.5% increase compared to control sites^49^; however, another RCT in which DBS testing for HCV was offered in prisons found an OR of 0.86 (95% CI: 0.71‐1.06) for the effect of the intervention on testing rate.^45^ Another comparative study reported a doubling in the number of tests performed in prisons and STI clinics during a national testing programme which involved awareness‐raising activities.^34^ Point‐of‐care rapid testing in clinics for people without healthcare coverage was found to significantly increase testing coverage to 98.2%, compared to standard serology testing performed elsewhere which had 64.2% coverage.^44^ One study which implemented testing of household contacts of HBV‐positive pregnant women through nurse‐led at‐home DBS testing reported a significant increase in coverage from 62% to 97% in antenatal services in which the intervention was implemented, compared to control sites where there was no increase observed in the same time frame.^50^ Another comparative study compared universal and risk‐based HCV screening for pregnant women in antenatal services and reported a positivity rate of 1.7% for universal screening compared to 1.3% for targeted screening; the difference was not significant.^47^ Information sessions and peer education increased coverage from 7% to 20% in a drug clinic, although this difference was not significant.^42^ Introduction of a clinic‐specific guideline in an STI clinic led to an increase in coverage from 4.7% to 13.6%.^53^ One comparative study reported on a clinical decision‐making tool initiative which included computer‐assisted self/personal interviewing vs paper‐and‐pen interviews in STI clinics. Both HBV and HCV testing coverage were highest (24% and 9%, respectively) after computer‐assisted personal interviewing.^60^

Acceptability and feasibility of testing initiatives in other healthcare settings were reported by three studies.^44^, ^58^, ^60^ One study on rapid DBS testing for HCV in pharmacies showed that patients found the pharmacy a good place to be tested, but some patients were suspicious when offered testing due to previous experience of discrimination at pharmacies.^58^ Among pharmacy staff, rapid DBS testing for HCV was found to be simple to perform. Rapid testing in a clinic for people with no health insurance was preferred over serological tests by 76% of the participants, with reasons including less stress with same‐day results and more practical use. Half of the clinical staff said that rapid testing simplified their consultation.^44^ A study that compared computer‐assisted interviewing to paper‐and‐pen interviews in STI clinics found that computer‐assisted interviewing encouraged the disclosure of sexual risk‐taking behaviour more.^60^

### 3.4 Testing initiatives in community settings

Forty‐one studies were retrieved that formed the evidence base for the effectiveness of testing initiatives and interventions in community settings.^34^, ^43^, ^64^, ^65^, ^66^, ^67^, ^68^, ^69^, ^70^, ^71^, ^72^, ^73^, ^74^, ^75^, ^76^, ^77^, ^78^, ^79^, ^80^, ^81^, ^82^, ^83^, ^84^, ^85^, ^86^, ^87^, ^88^, ^89^, ^90^ Results of initiatives performed in community settings are presented in Table 4. Results for all intervention types are presented first, stratified by setting type.

**Table 4:** Evidence base for the effectiveness of testing initiatives in community settings

Intervention	Studies included Quality of evidence	Target population	Test offer	Coverage/number of tests/tested^c^	Positivity rate
All per setting	**HBV**				
	N = 18 studies 65, 66, 69, 72, 73, 74, 75, 76, 78, 79, 82, 83, 84, 85, 86, 87, 88, 89 Quality: 3 studies acceptable 15 studies NA	Community‐based testing sites (n = 2) (sample size: 512 and 744)		Coverage: 37% and 71.1%	8.3% and 9.4%
		Outreach (n = 11) (sample size: 30‐86 000)	100%	Number tested/tests: 299‐1090 Coverage: 9.8%‐76.2% Comparative study coverage: 1.5% control; 42.8% intervention (outreach education + testing at health centre); 59.7% intervention (outreach education + onsite test) Comparative study: Coverage: 53.3% DBS postal kit; 83.3% nurse‐led sauna outreach testing; 100% standard STI clinic testing	0%‐12.4% Comparative study: 0% control; 2.1% intervention (outreach education + testing at health centre); 4.8% intervention (outreach education + onsite test)
		Drug services/ harm reduction programmes (n = 2) (sample size: intervention 27, control 28; 391)		Coverage: 49% Comparative study coverage: Intervention (oral) 92.6%; control (referral) 7.4%	20% (anti‐HBc)
		Online tools (n = 3) (sample size: 265 and 1400^a^)		Coverage: 16.2% 4305 self‐sampling kits requested; 48% of requested kits returned Comparative study coverage: 43.9% intervention (behavioural tailoring plus cultural tailoring) (OR 0.94 95% CI 0.69‐1.26); 43.5% intervention (behavioural tailoring) (OR 0.88 95% CI 0.65‐1.19) 46.0% control	0% and 0.2%
	**HCV**				
	N = 23 studies^34^, ^43^, ^64^, ^65^, ^67^, ^68^, ^69^, ^70^, ^71^, ^72^, ^73^, ^74^, ^75^, ^77^, ^78^, ^79^, ^80^, ^81^, ^82^, ^83^, ^88^, ^89^, ^90^ Quality: 3 studies acceptable 20 studies NA	Community‐based testing sites (n = 3) (sample size: 32‐744)		Number tested/tests: 95 Coverage: 37% and 71.1%	0%‐6.3%
		Outreach (n = 7) (sample size: 30‐65 000)	100%	Number tested/tests: 491‐520 Coverage: 9.8%‐76.2% Comparative study coverage: 1.5% control; 42.8% intervention (outreach education + testing at health centre); 59.7% intervention (outreach education + onsite test) Comparative study coverage: Coverage: 53.3% DBS postal kit; 83.3% nurse‐led sauna outreach testing; 100% standard STI clinic testing	0.8%‐37.6% Comparative study: 0% control; 3.2% intervention (outreach education + testing at health centre); 2.8% intervention (outreach education + onsite test) Comparative study: HBV/HCV active; cleared infection 6.25%; 25% DBS postal kits 0%; 4% nurse‐led sauna outreach testing 0%; 0% standard STI clinic testing
		Drug services/harm reduction programmes (n = 8) (sample size: 27‐5399)		Comparative study: 67 tests before 973 during Coverage: 42%‐84.2% Comparative study coverage: Intervention (oral) 100%; control (referral) 7.4% Comparative study: 44% intervention; 51% control	18%‐53% Comparative study: 15% intervention; 14% control Comparative study: 19.4% before; 38.1% during
		Online tools (n = 2) (sample size: 265 and 9653)	15.3%	Coverage: 4.4% and 16.2%	4.5% and 4.6%
		Combined settings (n = 3) (sample size: 240 and 564^a^)	73%	Number tested/tests: 266 Coverage: 37% and 72%	4.8%‐35%
Risk group testing	**HBV**				
	N = 16 studies^65^, ^66^, ^69^, ^72^, ^73^, ^75^, ^76^, ^78^, ^79^, ^82^, ^83^, ^84^, ^85^, ^86^, ^88^, ^89^ Quality: 3 studies acceptable 13 studies NA	Drug users (n = 3) (sample size: intervention 27, control 28; 391^a^)	100%	Coverage: 49%‐76.2% Comparative study coverage: intervention (oral) 92.6%; control (referral) 7.4%	0% 20% (anti‐HBc)
		Homeless (n = 1) (sample size: NR)		Number tested/tests: 491	12.4%
		Migrants (n = 9) (sample size: 744‐65 000^a^)		Number tested/tests: 299‐1090 Coverage: 9.8%‐71.1% Comparative study coverage: 43.9% intervention (behavioural tailoring plus cultural tailoring) (OR 0.94 95% CI 0.69‐1.26); 43.5% intervention (behavioural tailoring) (OR 0.88 95% CI 0.65‐1.19) 46.0% control	0.6%‐8.7%
		MSM (n = 2) (sample size: 30‐265)		Coverage: 16.2% Comparative study: coverage: 53.3% DBS postal kit; 83.3% outreach sauna nurse; 100% standard STI clinic	0% Comparative study: HBV/HCV active; cleared infection 6.25%; 25% DBS postal kits 0%; 4% nurse‐led sauna outreach testing 0%; 0% standard STI clinic testing
		Underprivileged people^b^ (n = 1) (sample size: 811 control; 222 intervention; 243 intervention + onsite test		Comparative study coverage: 1.5% control; 42.8% intervention (outreach education + testing at health centre); 59.7% intervention (outreach education + onsite test)	Comparative study: 0% control; 2.1% intervention (outreach education + testing at health centre); 4.8% intervention (outreach education + onsite test)
	**HCV**				
	N = 20 studies 43, 64, 65, 67, 68, 69, 70, 71, 72, 73, 75, 76, 78, 79, 80, 81, 82, 83, 89, 90 Quality: 2 studies acceptable 18 studies NA	Drug users (n = 6) (sample size: 27‐9653)	100%	Number tested/tests: 266 Coverage: 49%‐84.2% Comparative study coverage: intervention (oral) 100%; control (referral) 7.4%	31.2%‐41%
		PWID (n = 3) (sample size: 240 and 3463^a^)	73%	Number tested/tests: 24‐202 Coverage: 42% and 72%	18% and 20.3%
		Homeless (n = 2) (sample size: NR)		Number tested/tests: 95‐491	6.3% and 13%
		Migrants (n = 5) (sample size: 744‐65 000)		Number tested/tests: 520 Coverage: 9.8%‐71.1%	0.3%‐4.7%
		MSM (n = 2) (sample size: 30‐265)		Coverage: 16.2% Comparative study: coverage: 53.3% DBS postal kit; 83.3% outreach sauna nurse; 100% standard STI clinic	4.6% Comparative study: HBV/HCV active; cleared infection 6.25%; 25% DBS postal kits 0%; 4% nurse‐led sauna outreach testing 0%; 0% standard STI clinic testing
		Underprivileged people^b^ (n = 1) (sample size: 811 control; 222 intervention; 243 intervention + onsite test		Comparative study coverage: 1.5% control; 42.8% intervention (outreach education + testing at health centre); 59.7% intervention (outreach education + onsite test)	Comparative study: 0% control; 3.2% intervention (outreach education + testing at health centre); 2.8% intervention (outreach education + onsite test)
		Risk groups (n = 1) (sample size: 9653)	15.3%	Coverage: 4.4%	4.5%
Novel testing	**HBV**				
	N = 8 studies 65, 69, 73, 74, 75, 85, 87, 88 Quality: 1 study acceptable 7 studies NA	Drug users (n = 3) (sample size: intervention 27, control 28; 391^a^)	100%	Coverage (DBS): 49% Coverage (FibroScan): 76.2% Comparative study coverage: intervention (oral) 92.6%; control (referral) 7.4%	0% 20% (anti‐HBc)
		Migrants (n = 2) (sample size: 21 000 and 65 000)		Number tested/tests (DBS): 229‐1126	8.7%
		MSM (n = 1) (sample size: 30 per group)		Comparative study: coverage: 53.3% DBS postal kit; 83.3% outreach sauna nurse; 100% standard STI clinic	Comparative study: HBV/HCV active; cleared infection 6.25%; 25% DBS postal kits 0%; 4% nurse‐led sauna outreach testing 0%; 0% standard STI clinic testing
		Students (n = 1) (sample size: 512)		Coverage (DBS): 37%	9.4%
		General pop (n = 1) (sample size: NR)		4305 self‐sampling kits requested; 48% of requested kits returned	0.2%
	**HCV**				
	N = 13 studies^34^, ^43^, ^64^, ^65^, ^67^, ^68^, ^69^, ^73^, ^74^, ^75^, ^80^, ^81^, ^88^ Quality: 1 study acceptable 12 studies NA	Drug users (n = 7) (Sample size: 27‐1123)		Number tested/tests (DBS): 266 Coverage (DBS): 13%‐49% Coverage (FibroScan): 76.2% Comparative study coverage: Intervention (oral) 100%; control (referral) 7.4% Comparative study (DBS): 3‐fold increase in testing (RR = 3.5, *P* < 0.001)	27.5%‐53% Comparative study (DBS): 12‐fold increase in positives (RR = 12.1, *P* < 0.001)
		PWID (n = 2) (sample size: 240^a^)	73% (oral)	Number tested/tests (DBS): 202 Coverage (oral): 72%	20.3% (oral)
		Homeless (n = 1) (sample size: NR)		Number tested/tests (DBS): 95	6.3%
		Migrants (n = 1) (sample size: 65 000)		Number tested/tests (DBS): 520	0.8%
		MSM (n = 1) (sample size: 30 per group)		Comparative study: coverage: 53.3% DBS postal kit; 83.3% outreach sauna nurse; 100% standard STI clinic	Comparative study: HBV/HCV active; cleared infection 6.25%; 25% DBS postal kits 0%; 4% nurse‐led sauna outreach testing 0%; 0% standard STI clinic testing
		Students (n = 1) (sample size: 512)		Coverage (DBS): 37%	0%
Education	**HBV**				
	N = 3 studies 76, 79, 89 Quality: 1 study acceptable 2 studies NA	Targeted at‐risk groups Underprivileged people^b^ (n = 1) (sample size: 811 control; 222 intervention; 243 intervention + onsite test) Migrants (n = 2) (sample size: 1500 and 6337		Coverage: 11.2% and 31% Comparative study coverage: 1.5% control; 42.8% intervention (outreach education + testing at health centre); 59.7% intervention (outreach education + onsite test)	1.1% and 2.8% Comparative study 0% control; 2.1% intervention (outreach education + testing at health centre); 4.8% intervention (outreach education + onsite test)
	**HCV**				
	N = 4 studies^76^, ^77^, ^79^, ^89^ Quality: 1 study acceptable 3 studies NA	Targeted at‐risk groups Underprivileged people^b^ (n = 1) (sample size: 811 control; 222 intervention; 243 intervention + onsite test) Migrants (n = 2) (sample size: 1500 and 6337) PWID (n = 1) (sample size: 88)		Coverage: 11.2% and 31% Comparative study coverage: 1.5% control; 42.8% intervention (outreach education + testing at health centre); 59.7% intervention (outreach education + onsite test) Comparative study: 78% control; 85% intervention	0.3% and 2.4% Comparative study HCV: 0% control; 3.2% intervention (outreach education + testing at health centre); 2.8% intervention (outreach education + onsite test) Comparative study: 14% control; 15% intervention
Campaign	**HBV**				
	N = 4 studies^66^, ^76^, ^85^, ^86^ Quality: 4 studies NA	Targeted at‐risk groups Migrants (n = 4) (sample size: 6337‐29 000)		Number tested/tests: 299 and 1090 Coverage: 11.2% and 15.3%	2.8%‐8.7% 2.9% newly diagnosed
	**HCV**				
	N = 3 studies 34, 76, 90 Quality: 3 studies NA	Targeted at‐risk groups Migrants (n = 1) (sample size: 6337)		Coverage: 11.2%	0.3%
		Targeted at GPs and those at risk General pop. (n = 1) (sample size: 5339)		Comparative study number tested/tests: 67 before; 973 during	Comparative study: 19.4% before; 38.1% during
		Targeted at public General pop. (n = 1) (sample size: 9653)		15.3% website visitors offered testing. Coverage: 4.4%	4.5%
National programme	**HCV**				
	N = 2 studies^34^, ^70^ Quality: 2 studies NA	PWID (n = 1) (sample size: 3463)		Coverage: 42%	18%
		General population (n = 1) (sample size: 5399)		Comparative study: coverage before and after programme: 26% (in 2000); 62% (in 2008)	Comparative study: 19.4% before; 38.1% during
Communication & technology	**HBV**				
	N = 2 studies^78^, ^84^ Quality: 1 study acceptable 1 study NA	MSM (n = 1) (sample size: 265)		Coverage: 16.2%	0%
		Migrants (n = 1) (HCV n = 0, HBV n = 1 sample:432‐496)		Comparative study coverage: 43.9% BCT; 43.5% BT; 46.0% GI ORs (95%CI): BCT vs GI: 0.94 (0.69‐1.26) BT vs GI: 0.88 (0.65‐1.19)	
	**HCV**				
	N = 2 studies^78^, ^90^ Quality: 2 studies NA	MSM (n = 1) (sample size: 265)		Coverage: 16.2%	4.6%
		General population (n = 1) (sample size: 9653)		15.3% website visitors offered testing. Coverage: 4.4%	4.5%

Abbreviations: anti‐HBC, antibody to the hepatitis B core antigen; BCT, behavioural plus cultural tailoring; BT, behavioural tailoring; CI, confidence interval; DBS, dried blood spot; GI, generic information; HBV, hepatitis B virus; HCV, hepatitis C virus; HIV, human immunodeficiency virus; MSM, men who have sex with men; NA, not applicable; OR, odds ratio; PWID, people who inject drugs; STI, sexually transmitted infection; TB, tuberculosis.

a Denominator not reported by all studies.

b Unemployed, social assistance beneficiaries and seekers of political asylum living in long‐term shelters.

c Number of tests is only reported here if no data on coverage is available.

Coverage rates above 80% were reported by two testing initiatives conducted in community drugs services,^64^, ^65^ although the other six studies conducted in this setting reported lower coverage. HCV positivity rates were high in this setting.^34^, ^64^, ^70^, ^75^, ^77^, ^81^ Outreach testing activities and testing initiatives conducted in fixed community sites yielded coverage rates of up to 83.3% and 71.1%, respectively.^66^, ^69^, ^72^, ^73^, ^74^, ^76^, ^79^, ^80^, ^82^, ^83^, ^85^, ^86^, ^88^, ^89^ In general, online testing initiatives reported somewhat lower coverage rates relative to other settings (4.4% and 16.2%),^78^, ^90^ as well as low positivity rates for HBV (0% and 0.2%). Across all settings, novel testing initiatives yielded relatively high coverage rates in general, with eight out of fourteen studies that reported coverage testing more than 50% of the targeted population.^34^, ^43^, ^64^, ^65^, ^67^, ^68^, ^69^, ^73^, ^74^, ^75^, ^80^, ^81^, ^85^, ^87^, ^88^

Two comparative studies reported on novel testing initiatives targeted to risk groups. In community drug services, onsite oral sampling of drug users had a reported coverage of 100%, compared to 7.4% for standard serological testing which was performed offsite, at an STI clinic.^65^ Another study compared nurse‐delivered outreach screening at a sauna and self‐sampled DBS postal testing kits to standard screening at an STI clinic for MSM and reported coverage rates for the first 30 users of each service: 83.3% for outreach, 53.3% for DBS postal kits and 100% for standard screening. Positivity rates were higher for outreach (4% had a cleared HBV or HCV infection) and DBS postal kits (25% had a cleared HBV or HCV infection), compared to 0% for those tested in STI clinics. In addition, almost all STI clinic users had been tested previously, compared to just over half of sauna and postal kit users.^88^

An RCT compared outreach testing in shelters for underprivileged people, involving group education sessions and individual consultations during which subjects were offered testing, either at a health centre or onsite. Coverage was 42.8% at healthcare centres and 59.7% onsite. Coverage in a control group that received no intervention during the same time period was 1.5%.^79^ Educational sessions for PWID attending harm reduction centres contributed to an increased coverage from 44% to 85%, compared to an increase of 51 to 78% in a control group that received no educational intervention.^77^ During an HCV action plan in Scotland, implementation of DBS testing and awareness activities resulted in a 3‐fold increase in HCV testing coverage in community drug services (RR 3.5, *P* < 0.001) and a 12‐fold increase in positive test results (RR = 12.1, *P* < 0.001).In England, a national framework of activities to improve prevention, diagnosis and treatment of HCV led to an increase from 26% coverage in 2000 (prior to the national programme) to 62% coverage in 2008.^34^, ^70^ Lastly, a communication and technology HBV testing initiative involving Internet‐based recruitment of migrants for screening yielded coverage of 43.5%‐46.0%, although comparison between different strategies (behavioural tailoring, behavioural plus cultural tailoring or generic information) showed no differences.^84^

Eight studies provided data on the feasibility and acceptability of testing in community settings. Test acceptance rates ranged from 28.4% to 98.2%^68^, ^69^, ^74^, ^90^ with the highest rates reported in two studies which described outreach initiatives targeting drug users, one of which used oral sampling.^68^, ^69^ Another study reporting on an outreach testing initiative targeted at Chinese migrants reported that 100% of interviewed recipients found an information session prior to testing useful and just 5% responded that the test caused discomfort.^85^ A study describing an oral sampling initiative at a homeless shelter reported that 91% of the participants would agree to screening or thought an oral swab test was acceptable form of testing.^80^ An online platform offering home sampling kits for HBV, HIV and STIs reported that of the samples returned, 15% were provided insufficient blood and 39% of participants reported difficulties taking blood samples. However, 95% said they would use the online service again and 93% would recommend it to family and friends.^87^ A testing initiative targeted at university students with an acceptance rate of 37% reported that there was no indication or reports from students or staff that the testing offer and process were stigmatizing or undesirable.^74^ Lastly, a culturally targeted screening project involving campaigns and educational meetings for Turkish migrants was considered good and understandable by 97% of the participants.^76^

### 3.5 Testing initiatives in multiple/unspecified settings

In addition, two studies were retrieved which provided data on initiatives conducted in multiple settings or did not specify results per setting. One study examined the outcomes of a French national HCV prevention programme implemented in 1999 and reported that testing increased from 2000 to 2005 by 45% but decreased by 10% in 2006. Positivity rates decreased from 4.3% to 2.9%.^91^ A further study describing a national testing programme which involved awareness‐raising activities reported outcomes across all settings; 19 058 tests were taken before; and 29 045 tests were taken during the programme and positivity rates decreased from 9.6% to 6.8%.^34^

## 4 DISCUSSION

We undertook a comprehensive systematic review to collect, synthesize and analyse available data on HBV/HCV testing strategies across the EU/EEA. Available evidence on testing strategies was analysed qualitatively across primary health care, hospital settings, other healthcare settings and community settings.

Primary health care is usually the first point of contact that patients will have with the health care system. Therefore, it is an important setting for testing a number of population groups that may not present to other settings and may be especially important for specific population groups including ex‐PWID.^92^ However, according to a UK study, of all HCV diagnostic tests performed in a pool of sentinel laboratories, only 16% were requested by GPs.^93^ Overall, the evidence retrieved on the effectiveness of testing interventions to improve HBV and HCV testing coverage in primary health care was limited, largely restricted to Western European countries, and focused mainly on targeted test offer to risk groups such as migrants, PWID and homeless. A previous meta‐analysis assessing the effectiveness of targeted HCV testing interventions found these strategies to be effective in diagnosing cases and increasing uptake of treatment, particularly when strategies involved practitioners.^18^ In this review, limited evidence indicated that educational interventions targeting GPs and campaigns targeting the public, GPs or risk groups may have benefit, particularly when education and campaigns are combined, and that offering HCV testing in this setting was acceptable to patients. A recent EU‐funded project investigated options to further expand testing opportunities for migrant communities in this setting and has produced a useful toolkit that may support its implementation.^94^

Hospitals are another important location for testing, with more than half of all HCV tests performed in UK sentinel laboratories requested by hospital‐based clinicians, especially hepatology and infectious disease departments.^93^ However, the evidence on testing interventions aimed at improving HBV and HCV testing in hospital settings retrieved was relatively limited. In many studies, testing for HBV and HCV was performed as part of a BBV screening test including HIV testing. Specific hospital departments could be strategic settings to capture patients belonging to certain risk groups and encourage and facilitate testing. Targeted testing to individuals from risk groups (migrants and psychiatric patients) was implemented in hospital departments with generally high positivity rates and fairly high coverage levels in studies involving hospital inpatients or outpatients attending the hospital for reasons other than testing, although uptake was lower when participants were invited to come to the hospital for the sole purpose of testing. This emphasizes the importance of practitioners considering opportunistic test offer for patients that may be at risk. Two studies on universal testing in emergency departments reported lower positivity rates compared to other strategies; however, this could be an important strategy for case finding in areas that are known to have a high prevalence or burden of disease, or where injecting drug use is prevalent.

Evidence relating to other healthcare settings found similar variation in coverage and positivity rates which were often high when risk groups were targeted. This suggests that offering testing to groups at high risk of infection at locations they specifically attend for health purposes, such as health clinics for migrants, could be an effective method for case finding. Locations frequently visited by certain groups, where staff are medically trained, present an opportunity that can be exploited for testing. For example, individuals receiving OST access pharmacies on a regular basis to receive medication. People receiving OST were found to be more likely to accept DBS testing at the pharmacy than testing from other providers.^58^ Furthermore, pharmacies in some countries can provide treatment for those found to be infected.^59^ Novel testing approaches such as rapid testing and DBS were frequently employed in testing initiatives in the various healthcare settings and were found to be effective in increasing coverage in a number of comparative settings, as well as being highly acceptable among users and testing staff. Rapid testing allows point‐of‐care testing, simplifying the testing process as those tested do not have to visit outside testing centres or wait to pick up results, although it is crucial to ensure that individuals testing positive are linked to ongoing care. Another strategy identified that may help to improve testing coverage in other healthcare settings included implementation of clinic‐specific guidelines.^53^ These may be particularly useful in countries where national hepatitis testing guidelines are not available.^8^

Community‐based testing services, both fixed‐site and outreach‐based, represent potential sites to target specific population groups and individuals who may be at increased risk of infection and who are not in contact with formal health services. Testing in community settings can be adapted and made accessible for these groups in order to maximize testing uptake^15^; however, a gap in policy on community and outreach testing was reported by some EU countries suggesting that it is not always implemented at scale.^8^ Yet, we identified considerable evidence supporting the implementation of community‐based testing services. According to the evidence, testing services in community settings often resulted in high coverage and high positivity rates, particularly when targeting MSM, people with migration background and drug users, although influenced by the underlying epidemiology. Outreach activities, including short‐term testing facilities, mobile testing services and street‐based outreach, were also shown to be highly effective in targeting these population groups, while being highly adaptable. Available evidence also suggested that use of oral sampling and DBS increase testing coverage and oral sampling was considered acceptable in community‐based services. Compared to other infectious diseases, the market for HBV and HCV rapid tests is less abundant, and the scale of implementation is comparatively limited.^8^ However, DBS and rapid tests are recommended by the WHO to facilitate testing in settings where no laboratory is available.^95^ Finally, educational interventions, campaigns and national programmes were also found to increase community‐based testing coverage for HBV and HCV, including among migrant communities. An earlier systematic review on chronic HBV testing in community settings identified common features of successful screening interventions that included strategies to increase community awareness and knowledge and incorporated the target population's values in the design and implementation of the programme and were able to provide low cost or free access to care.^21^

Contact tracing and partner notification for HBV and HCV have recognized public health benefits, such as controlling the spread, reducing morbidity and mortality, reaching people with asymptomatic infection and people who do not present for diagnosis, counselling and treatment^96^ and have been shown to be effective.^50^ Partner notification may be implemented in different ways,^97^ and approaches designed to meet diverse structural, societal and legal barriers. However, our study identified very limited evidence on this approach, possibly due to its suboptimal implementation in EU/EEA, as reported in a previous study.^98^

A recent WHO guideline recommended that HCV screening in birth cohorts of older persons at higher risk of infection and morbidity may be applied in populations with overall lower general prevalence, where indicated by the local epidemiology.^95^, ^99^ Recent epidemiological studies to assess the relevance of such an approach have been performed in some EU/EEA countries, demonstrating the growing interest in this testing strategy and suggesting a possible role of this approach in the elimination effort.^100^, ^101^, ^102^, ^103^ The evidence resulting from our study, however, indicated that the utility of birth cohort screening is dependent on the local epidemiology and context of the screening.

Self‐sampling and self‐testing are relatively new testing modalities that have the potential to increase testing coverage. For HIV, use of self‐sampling or self‐testing kits is already authorized in a limited number of countries.^104^ For HIV, self‐testing has been associated with increased testing uptake among men in a number of RCTs and is recommended as a testing approach by the WHO.^105^ We identified very limited evidence on self‐sampling kits and no evidence on self‐testing for HBV/HCV. Rather than indicating a lack of interest or limited applicability, this may reflect the lack of technology availability for HBV/HCV self‐testing. However, as these technologies become available in the future, self‐testing may become a potentially important approach to expand access to testing.^106^ Transferability of the evidence and relevance of these approaches from the HIV field may contribute to this process.

Barriers to testing exist at individual, healthcare provider and institutional levels which can impede case‐finding efforts. In general, the asymptomatic course of HBV/HCV, low levels of knowledge and awareness and fear of stigma and discrimination may prevent people from seeking testing or accepting test offer.^32^, ^58^, ^107^, ^108^, ^109^, ^110^, ^111^, ^112^ For vulnerable populations, health and social problems, unstable or unstructured lives and poverty can be barriers, for PWID venous access can be an issue and for people with a migration background, culture, faith and language and their perceptions and understanding of the health care system may present barriers to testing.^29^, ^58^, ^109^, ^110^, ^111^, ^113^ The proximity of health care services to individuals, low awareness among health care professionals and forgetting to test are barriers that may exist at the testing provider level.^58^, ^109^, ^113^ Barriers specific to health care include administrational limitations, time limitations and the GP's relationship with their patients.^29^, ^32^, ^113^ Potential barriers specific to community include inconvenient testing facilities and lack of advocacy and promotion.^109^, ^112^ At an institutional level, pressures, capacity and funding shortages in primary healthcare sectors and community organizations can present barriers to testing.^109^, ^110^ Implementing certain testing strategies may help to alleviate many of these barriers; for example, educational initiatives, campaigns and other health promotional activities could help to raise awareness and knowledge within certain populations, the wider public and healthcare professionals. Using novel techniques such as DBS or oral sampling can bypass any issues with venous access. Demedicalizing services and bringing them out into the community could circumvent barriers which exist within healthcare settings. Implementing testing activities in a range of settings could lessen the impact of setting‐specific barriers.

The comparability of data retrieved in the systematic review was limited by the large degree of heterogeneity between studies in the outcomes measured, the specific populations targeted, recruitment and length of interventions, and whether testing initiatives were combined with health promotional activities, all of which could influence the success of the intervention. In addition, the lack of a threshold for sufficient levels of testing uptake or coverage precluded quantitative analysis. The majority of studies did not use a comparator to assess effectiveness of interventions. Furthermore, none of the studies retrieved assessed the long‐term outcomes of interventions such as the impact of testing on prevalence and incidence over time. Few studies were deemed to be of high quality and the majority had a study design which precluded formal quality assessment. For these studies, the most common quality issues noted were a lack of clarity or detail in methodology (eg data collection methods), limited description of the study population and unclear or inappropriate denominator. A significant proportion (16%) of the evidence base of the review was from included conference abstracts, that is non‐peer‐reviewed literature for which methods and results were often extremely limited and quality assessment was not possible.

In conclusion, evidence was retrieved for successful testing approaches applied in primary health care, hospital and other healthcare settings and community settings, although within most settings the evidence was fairly limited. Testing approaches targeting population groups at high risk of HBV/HCV were found to be viable in various settings, and there was evidence that other interventions such as awareness campaigns, education and the implementation of testing in the context of a national strategy may improve coverage by helping to overcome some of the barriers to testing. DBS was also associated with increased testing coverage in several different settings and other potentially effective testing strategies include contact tracing, home sampling and birth cohort testing. Further research and an understanding of local factors including the epidemiology are needed to provide policymakers with a clearer overview of which initiatives will be most successful in improving testing uptake and yielding high positivity rates. However, combining a diverse set of testing opportunities within national testing strategies may lead to higher impact in terms of both testing coverage and reduction of the undiagnosed fraction. Elimination of viral hepatitis in Europe by 2030, according to the WHO regional goal, will require diagnosing those infected and ensuring linkage to appropriate prevention, care, treatment and support services.^14^, ^95^ Implementation of diversified set of effective, evidence‐based testing strategies, particularly among vulnerable and hard‐to‐reach populations is a vital step in realizing this goal.

## CONFLICT OF INTEREST

The authors have no conflict of interest to declare.

## AUTHORS’ CONTRIBUTIONS

LT, ED, LM, IV, EB, AAG contributed to the design of the project and the development of the study protocol, LT coordinated the study. LM, AA, EB, LT performed the systematic review, including data collection and data analysis. All authors contributed to data interpretation, manuscript drafting and review. LM drafted the first version of the manuscript.
