# Supplementary material for: Hepatitis B and C testing strategies in healthcare and community settings in the EU/EEA: A systematic review
Source: J Viral Hepat. 2019 Aug 16;26(12):1431–53. doi: 10.1111/jvh.13182 (PMC6899601; doi:10.1111/jvh.13182)
Supplement: Supplementary file 1 [file JVH-26-1431-s001.docx]

**Supplementary material**

**Hepatitis B and C testing strategies in healthcare and community settings in the EU/EEA: a systematic review**

## PICO questions

##### Table 1. PICO on topic testing

| **1** | **What approaches to increase coverage and uptake of hepatitis B/C testing have been implemented in the EU/EEA and how effective are they?** |
| --- | --- |
| P | General population and population subgroups possibly at risk for and/or with a high burden of hepatitis B or C |
| I | Interventions to increase coverage and uptake of hepatitis B/C testing |
| C | No intervention/other intervention |
| O | *Qualitative outcomes:*   - Description of intervention - Acceptance/barriers to testing - Feasibility of testing intervention   *Quantitative outcomes:*   - Offer of test - Uptake and coverage of testing - Positivity rate/diagnosis rate - Changes in prevalence/incidence |

##### Table 2: PICO on topic linkage to care

| **2** | **What linkage to care strategies have been implemented in the EU/EEA for hepatitis B/C and how effective are they?** |
| --- | --- |
| P | Newly diagnosed patients/persons testing negative |
| I | Interventions to increase linkage to care |
| C | No intervention/other intervention |
| O | *Qualitative outcomes:*   - Description of intervention - Acceptance/barriers to linkage to care - Feasibility of intervention   *Quantitative outcomes:*   - Proportion of persons referred to care/preventative care - Proportion of persons linked to care/preventative care - Proportion of persons receiving care/preventative care |

## Search strings

**PubMed search strings**

#### #a string for Hepatitis B and Hepatitis C virus

Hepatitis B[MeSH] OR Hepatitis B virus[Mesh] OR Hepatitis B Antigens[Mesh] OR Hepatitis B Antibodies[Mesh] OR hepatitis b[tiab] OR hbv[ti] OR Hep B[tiab] OR hbsag[tiab] OR “hbs ag”[tiab] OR Hepatitis C[MeSH] OR Hepacivirus[MeSH] OR Hepatitis C Antibodies[MeSH] OR Hepatitis C Antigens[Mesh] OR Hepatitis C[tiab] OR hepaciviru*[tiab] OR hcv[ti] OR hep c[tiab] OR blood borne virus*[tiab] or bbv[ti]

#### #b string for testing

“Mass Screening”[Mesh] OR “Mandatory Testing”[Mesh] OR “Point-of-Care Testing”[Mesh] OR “Dried Blood Spot Testing”[Mesh] OR "Early Diagnosis"[Mesh:NoExp] OR “mandatory test”[tiab] OR “mandatory testing”[tiab] OR “Point-of-Care”[tiab] OR “Dried Blood Spot”[tiab] OR screen[tiab] OR screened[tiab] OR screening[tiab] OR “hepatitis B testing”[tiab] OR “HBV testing”[tiab] OR “hepatitis B test”[tiab] OR “HBV test”[tiab] OR “testing for hepatitis B”[tiab] OR “testing for hbv”[tiab] OR “test for hepatitis B”[tiab] OR “test for hbv”[tiab] OR “hepatitis C testing”[tiab] OR “HCV testing”[tiab] OR “hepatitis C test”[tiab] OR “HCV test”[tiab] OR “testing for hepatitis C”[tiab] OR “testing for hcv”[tiab] OR “test for hepatitis C”[tiab] OR “test for hcv”[tiab] OR “bedside test”[tiab] OR “bedside testing”[tiab] OR diagnos*[tiab] OR (case*[tiab] AND (find*[tiab] OR identif*[tiab] OR detect*[tiab]))

#### #c string for linkage to care

((uptake[tiab] OR adhere*[tiab] OR comply*[tiab] OR compliance[tiab] OR complied[tiab] OR retain*[tiab] OR retention[tiab] OR link*[tiab] OR initiat[tiab] OR begin*[tiab] OR began[tiab] OR start*[tiab] enter*[tiab] OR commence*[tiab] OR refer*[tiab]) AND (care[tiab] OR healthcare[tiab] OR treat*[tiab] OR therap*[tiab])) OR prevent[tiab] OR prevention[tiab] OR preventative[tiab]

#### #d string for intervention

"Program Evaluation"[Mesh] OR "Health Promotion"[Mesh] OR "Feasibility Studies"[Mesh] OR "Pilot Projects"[Mesh] OR intervention*[tiab] OR approach*[tiab] OR program*[tiab] OR campaign*[tiab] OR promot*[tiab] OR pilot*[tiab] OR evaluation*[tiab] OR appraisal*[tiab] OR assessment*[tiab] OR feasibility[tiab] OR service*[tiab] OR strateg*[tiab] OR outreach[tiab] OR scheme*[tiab] OR project*[tiab] OR policy[tiab] OR policies[tiab] OR audit*[tiab] OR (community[tiab] AND based[tiab])

#### #d string for EU/EEA

(((((((Europe*[ad] OR Europa*[ad] OR EU[ad] OR EEA[ad] OR "EU/EEA"[ad] OR ECSC[ad] OR Euratom[ad] OR Eurozone[ad] OR EEC[ad] OR ec[ad] OR (Schengen[ad] AND (area[ad] OR countr*[ad] OR region*[ad] OR state[ad] OR states[ad])) OR Euroregion[ad] OR Euroregions[ad] OR Balkan[ad] OR Balkans[ad] OR Baltic[ad] OR (Mediterranean[ad] AND (area[ad] OR countr*[ad] OR region*[ad] OR state[ad] OR states[ad])) OR (Alpine[ad] AND (area[ad] OR countr*[ad] OR region*[ad] OR state[ad] OR states[ad])) OR Scandinavia[ad] OR Scandinavian[ad] OR "Nordic country"[ad] OR "Nordic countries"[ad] OR "Nordic state"[ad] OR "Nordic states"[ad] OR Danubian[ad] OR "Iberian peninsula"[ad] OR "Peninsula iberica"[ad] OR "Peninsule Iberique"[ad] OR "Iberiar Penintsula"[ad] OR Iberia[ad] OR Czechoslovakia[ad] OR "Czecho Slovakia"[ad] OR Ceskoslovensko[ad] OR "Cesko slovensko"[ad] OR Benelux[ad] OR Fennoscandia[ad] OR "Fenno Scandinavia"[ad] OR Fennoskandi*[ad] OR (Visegrad[ad] AND (Group[ad] OR Four[ad] OR Triangle[ad])) OR "Visegradska ctyrka"[ad] OR "Visegradska skupina"[ad] OR "Visegradi Egyuttmukodes"[ad] OR "Visegradi negyek"[ad] OR "Grupa Wyszehradzka"[ad] OR "Vysehradska skupina"[ad] OR "Vysehradska stvorka"[ad])) OR ("Iceland"[Mesh] OR Iceland[tw] OR Icelandic*[tw] OR islenska*[tw] OR Icelander*[tw] OR islendinga*[tw] OR Islendigar[tw] OR Inslenska[tw] OR Reykjavik[tw] OR Reykjavikurborg[tw] OR Hofudborgarsvaedi[tw] OR Sudurnes[tw] OR Vesturland[tw] OR Vestfirdir[tw] OR Westfjords[tw] OR Nordurland[tw] OR Austurland[tw] OR Sudurland[tw] OR Kopavogur[tw] OR Hafnarfjordur[tw] OR Akureyri[tw] OR Gardabaer[tw] OR Mosfellsbaer[tw] OR Keflavik[tw] OR Akranes[tw] OR Selfoss[tw] OR Seltjarnarnes[tw]) OR ((Iceland[ad] OR Icelandic*[ad] OR islenska*[ad] OR Icelander*[ad] OR islendinga*[ad] OR Islendigar[ad] OR Inslenska[ad] OR Reykjavik[ad] OR Reykjavikurborg[ad] OR Hofudborgarsvaedi[ad] OR Sudurnes[ad] OR Vesturland[ad] OR Vestfirdir[ad] OR Westfjords[ad] OR Nordurland[ad] OR Austurland[ad] OR Sudurland[ad] OR Kopavogur[ad] OR Hafnarfjordur[ad] OR Akureyri[ad] OR Gardabaer[ad] OR Mosfellsbaer[ad] OR Keflavik[ad] OR Akranes[ad] OR Selfoss[ad] OR Seltjarnarnes[ad]) OR (Norway[ad] OR Norwegian*[ad] OR Norge[ad] OR Noreg[ad] OR Norgga[ad] OR Akershus[ad] OR "Aust Agder"[ad] OR Buskerud[ad] OR Finnmark[ad] OR Hedmark[ad] OR Hordaland[ad] OR "More og Romsdal"[ad] OR "More and Romsdal"[ad] OR "More Romsdal"[ad] OR Nordland[ad] OR Trondelag[ad] OR Oppland[ad] OR Oslo[ad] OR Ostfold[ad] OR Rogaland[ad] OR "Sogn og fjordane"[ad] OR "Sogn and fjordane"[ad] OR "sogn fjordane"[ad] OR Telemark[ad] OR Troms[ad] OR Romsa[ad] OR Romssa[ad] OR "Vest Agder"[ad] OR Vestfold[ad] OR Bergen[ad] OR Stavanger[ad] OR Sandnes[ad] OR Trondheim[ad] OR Trondhjem[ad] OR Kaupangen[ad] OR Nidaros[ad] OR Drammen[ad] OR Fredrikstad[ad] OR Skien[ad] OR Tromso[ad] OR Sarpsborg[ad]) OR (Liechtenstein[ad] OR Lienchtensteiner*[ad] OR Balzers[ad] OR Eschen[ad] OR Gamprin[ad] OR Mauren[ad] OR Planken[ad] OR Ruggell[ad] OR Schaan[ad] OR Schellenberg[ad] OR Triesen[ad] OR Triesenberg[ad] OR Vaduz[ad]))) OR (Iceland[ad] OR Icelandic*[ad] OR islenska*[ad] OR Icelander*[ad] OR islendinga*[ad] OR Islendigar[ad] OR Inslenska[ad] OR Reykjavik[ad] OR Reykjavikurborg[ad] OR Hofudborgarsvaedi[ad] OR Sudurnes[ad] OR Vesturland[ad] OR Vestfirdir[ad] OR Westfjords[ad] OR Nordurland[ad] OR Austurland[ad] OR Sudurland[ad] OR Kopavogur[ad] OR Hafnarfjordur[ad] OR Akureyri[ad] OR Gardabaer[ad] OR Mosfellsbaer[ad] OR Keflavik[ad] OR Akranes[ad] OR Selfoss[ad] OR Seltjarnarnes[ad]) OR ((((("Liechtenstein"[Mesh] OR Liechtenstein[tw] OR Lienchtensteiner*[tw] OR Balzers[tw] OR Eschen[tw] OR Gamprin[tw] OR Mauren[tw] OR Planken[tw] OR Ruggell[tw] OR Schaan[tw] OR Schellenberg[tw] OR Triesen[tw] OR Triesenberg[tw] OR Vaduz[tw])) OR ("Norway"[Mesh] OR Norway[tw] OR Norwegian*[tw] OR Norge[tw] OR Noreg[tw] OR Norgga[tw] OR Akershus[tw] OR "Aust Agder"[tw] OR Buskerud[tw] OR Finnmark[tw] OR Hedmark[tw] OR Hordaland[tw] OR "More og Romsdal"[tw] OR "More and Romsdal"[tw] OR "More Romsdal"[tw] OR Nordland[tw] OR Trondelag[tw] OR Oppland[tw] OR Oslo[tw] OR Ostfold[tw] OR Rogaland[tw] OR "Sogn og fjordane"[tw] OR "Sogn and fjordane"[tw] OR "sogn fjordane"[tw] OR Telemark[tw] OR Troms[tw] OR Romsa[tw] OR Romssa[tw] OR "Vest Agder"[tw] OR Vestfold[tw] OR Bergen[tw] OR Stavanger[tw] OR Sandnes[tw] OR Trondheim[tw] OR Trondhjem[tw] OR Kaupangen[tw] OR Nidaros[tw] OR Drammen[tw] OR Fredrikstad[tw] OR Skien[tw] OR Tromso[tw] OR Sarpsborg[tw])) OR ("Iceland"[Mesh] OR Iceland[tw] OR Icelandic*[tw] OR islenska*[tw] OR Icelander*[tw] OR islendinga*[tw] OR Islendigar[tw] OR Inslenska[tw] OR Reykjavik[tw] OR Reykjavikurborg[tw] OR Hofudborgarsvaedi[tw] OR Sudurnes[tw] OR Vesturland[tw] OR Vestfirdir[tw] OR Westfjords[tw] OR Nordurland[tw] OR Austurland[tw] OR Sudurland[tw] OR Kopavogur[tw] OR Hafnarfjordur[tw] OR Akureyri[tw] OR Gardabaer[tw] OR Mosfellsbaer[tw] OR Keflavik[tw] OR Akranes[tw] OR Selfoss[tw] OR Seltjarnarnes[tw]))) OR (("European Union"[Mesh] OR "Europe"[Mesh:noexp] OR Europe*[tw] OR Europa*[tw] OR EU[tw] OR EEA[tw] OR "EU/EEA"[tw] OR ECSC[tw] OR Euratom[tw] OR Eurozone[tw] OR EEC[tw] OR ec[tw] OR (Schengen[tw] AND (area[tw] OR countr*[tw] OR region*[tw] OR state[tw] OR states[tw])) OR Euroregion[tw] OR Euroregions[tw] OR "Europe, Eastern"[Mesh:noexp] OR "Balkan Peninsula"[Mesh] OR Balkan[tw] OR Balkans[tw] OR "Baltic States"[Mesh] OR Baltic[tw] OR "Mediterranean Region"[Mesh] OR (Mediterranean[tw] AND (area[tw] OR countr*[tw] OR region*[tw] OR state[tw] OR states[tw])) OR (Alpine[tw] AND (area[tw] OR countr*[tw] OR region*[tw] OR state[tw] OR states[tw])) OR "Scandinavian and Nordic Countries"[Mesh] OR Scandinavia[tw] OR Scandinavian[tw] OR "Nordic country"[tw] OR "Nordic countries"[tw] OR "Nordic state"[tw] OR "Nordic states"[tw] OR Danubian[tw] OR "Iberian peninsula"[tw] OR "Peninsula iberica"[tw] OR "Peninsule Iberique"[tw] OR "Iberiar Penintsula"[tw] OR Iberia[tw] OR Anatolia[tw] OR Anadolu[tw] OR Anatole[tw] OR Anatolian[tw] OR "Yugoslavia"[Mesh] OR Yugoslavia[tw] OR "Czechoslovakia"[Mesh] OR Czechoslovakia[tw] OR "Czecho Slovakia"[tw] OR Ceskoslovensko[tw] OR "Cesko slovensko"[tw] OR Benelux[tw] OR Fennoscandia[tw] OR "Fenno Scandinavia"[tw] OR Fennoskandi*[tw] OR (Visegrad[tw] AND (Group[tw] OR Four[tw] OR Triangle[tw])) OR "Visegradska ctyrka"[tw] OR "Visegradska skupina"[tw] OR "Visegradi Egyuttmukodes"[tw] OR "Visegradi negyek"[tw] OR "Grupa Wyszehradzka"[tw] OR "Vysehradska skupina"[tw] OR "Vysehradska stvorka"[tw]) OR ("Austria"[Mesh] OR Austria*[tw] OR Osterreich*[tw] OR Oesterreich*[tw] OR Ostosterreich[tw] OR Ostoesterreich[tw] OR Sudosterreich[tw] OR Sudoesterreich[tw] OR Westosterreich[tw] OR Westoesterreich[tw] OR Burgenland[tw] OR Carinthia[tw] OR Karnten[tw] OR Kaernten[tw] OR Niederosterreich[tw] OR Niederoesterreich[tw] OR Oberosterreich[tw] OR Oberoesterreich[tw] OR Salzburg[tw] OR Saizburg[tw] OR Styria[tw] OR Steiermark[tw] OR Tyrol[tw] OR Tirol[tw] OR Vorarlberg[tw] OR Vienna[tw] OR Wien[tw] OR Graz[tw] OR Linz[tw] OR Innsbruck[tw] OR Klagenfurt[tw] OR Villach[tw] OR Wels[tw] OR "St Polten"[tw] OR "St Poelten"[tw] OR "Sankt Polten"[tw] OR "Sankt Poelten"[tw] OR Dornbirn[tw]) OR ("Belgium"[Mesh] OR Belgi*[tw] OR Belge*[tw] OR Belg[tw] OR Brussel*[tw] OR Bruxelles[tw] OR Bruxelloise[tw] OR Walloon*[tw] OR Wallon*[tw] OR Vlaams[tw] OR Flander*[tw] OR Flandern[tw] OR Flandre[tw] OR Flemish[tw] OR Flamand[tw] OR Flemisch[tw] OR Flamisch*[tw] OR Vlaanderen[tw] OR Flamande[tw] OR Waals[tw] OR Antwerp*[tw] OR Anvers[tw] OR Henegouwen[tw] OR Hennegau[tw] OR Hainault[tw] OR Hainaut[tw] OR Liege[tw] OR Luik[tw] OR Luttich[tw] OR Limbourg[tw] OR Limburg[tw] OR Namur[tw] OR Namen[tw] OR Ostflandern[tw] OR Westflandern[tw] OR Ghent[tw] OR Gent[tw] OR Gand[tw] OR Charleroi[tw] OR Bruges[tw] OR Brugge*[tw] OR Schaerbeek[tw] OR Schaarbeek[tw] OR Anderlecht[tw] OR Leuven[tw] OR Louvain[tw]) OR ("Bulgaria"[Mesh] OR Bulgaria*[tw] OR Balgariya[tw] OR Balgarija[tw] OR Blagoevgrad*[tw] OR "Pirin Macedonia"[tw] OR Burgas[tw] OR Dobrich[tw] OR Gabrovo[tw] OR Haskovo[tw] OR Kardzhali[tw] OR Kurdzhali[tw] OR Kyustendil[tw] OR Lovech[tw] OR Lovec[tw] OR Montana[tw] OR Pazardzhik[tw] OR Pernik[tw] OR Pleven*[tw] OR Plovdiv[tw] OR Razgrad[tw] OR Rousse[tw] OR Ruse[tw] OR Rusenka[tw] OR Shumen[tw] OR Silistra[tw] OR Sliven[tw] OR Smolyan[tw] OR Sofia[tw] OR Sofyiska[tw] OR Sofiiska[tw] OR "Stara Zagora"[tw] OR Targovishte[tw] OR Varna[tw] OR "Veliko Tarnovo"[tw] OR Vidin[tw] OR Vratsa[tw] OR Vratza[tw] OR Yambol[tw]) OR ("Croatia"[Mesh] OR Croat*[tw] OR Hrvatsk*[tw] OR hrvati[tw] OR Bjelovar[tw] OR "Bjelovarsko bilogorska"[tw] OR "Brod Posavina"[tw] OR "Brodsko posavska"[tw] OR "Dubrovnik Neretva"[tw] OR "dubrovacko neretvanska"[tw] OR Zagreb[tw] OR Zagrebacka[tw] OR Istria[tw] OR Istarska[tw] OR Karlovacka[tw] OR Karlovac[tw] OR "Koprivnicko krizevacka"[tw] OR Koprivnica[tw] OR Krizevci[tw] OR "Krapina Zagorje"[tw] OR "Krapinsko zagorska"[tw] OR "Lika Senj"[tw] OR "Licko senjska"[tw] OR Medimurska[tw] OR Medimurje[tw] OR Osijek[tw] OR Baranja[tw] OR "Osjecko baranjska"[tw] OR "Pozega Slavonia"[tw] OR "Pozesko slavonska"[tw] OR "Primorje Gorski Kotar"[tw] OR "Primorsko goranska"[tw] OR "Sibensko kninska"[tw] OR "Sibensko kninske"[tw] OR Sibenik[tw] OR Knin[tw] OR Sisak[tw] OR "Sisacko moslavacka"[tw] OR Moslavina[tw] OR "Splitsko dalmatinska"[tw] OR Split[tw] OR Dalmatia[tw] OR Varazdin[tw] OR Varazdinska[tw] OR Viroviticko[tw] OR podravska[tw] OR Virovitica[tw] OR Podravina[tw] OR "Vukovarsko srijemska"[tw] OR Vukovar[tw] OR Srijem[tw] OR Zadar[tw] OR Zadarska[tw] OR Rijeka[tw] OR "Velika gorica"[tw] OR "Slavonski brod"[tw] OR Pula[tw]) OR ("Cyprus"[Mesh] OR Cyprus[tw] OR Cypriot*[tw] OR Kypros[tw] OR Kibris*[tw] OR kypriaki[tw] OR Kyprioi[tw] OR Nicosia[tw] OR Lefkosa[tw] OR Lefkosia[tw] OR Famagusta[tw] OR Magusa[tw] OR Ammochostos[tw] OR Gazimagusa[tw] OR Kyrenia[tw] OR Girne[tw] OR Keryneia[tw] OR Larnaca[tw] OR Larnaka[tw] OR Iskele[tw] OR Limassol[tw] OR Lemesos[tw] OR Limasol[tw] OR Leymosun[tw] OR Paphos[tw] OR Pafos[tw] OR Baf[tw] OR Strovolos[tw] OR Lakatamia[tw] OR Lakadamya[tw] OR "Kato Polemidia"[tw] OR "Kato Polemidhia"[tw] OR Aglandjia[tw] OR Eglence[tw] OR Aglantzia[tw] OR Aradhippou[tw] OR Aradippou[tw] OR Engomi[tw]) OR ("Czech Republic"[Mesh] OR Czech*[tw] OR Cesky[tw] OR Ceska[tw] OR Cech[tw] OR Cestina[tw] OR Prague[tw] OR Praha[tw] OR Prag[tw] OR Stredoces*[tw] OR Jihoces*[tw] OR Bohemia[tw] OR Bohemian[tw] OR Plzen*[tw] OR Pilsen[tw] OR Karlovars*[tw] OR "Karlovy Vary"[tw] OR Usteck*[tw] OR Usti[tw] OR Liberec*[tw] OR "Hradec Kralove"[tw] OR Kralovehradec*[tw] OR Pardubic*[tw] OR Olomouc*[tw] OR Olomoc[tw] OR Holomoc[tw] OR Moravskoslezs*[tw] OR Jihomorav*[tw] OR Moravia[tw] OR Moravian[tw] OR Morava[tw] OR Vysocina[tw] OR Zlin[tw] OR Zlinsk*[tw] OR "Ceske Budejovice"[tw] OR Budweis[tw] OR Brno[tw] OR Ostrava[tw]) OR ("Denmark"[Mesh] OR Denmark[tw] OR Danish*[tw] OR dane[tw] OR danes[tw] OR Danmark[tw] OR dansk*[tw] OR Hovedstaden[tw] OR Midtjylland[tw] OR Nordjylland[tw] OR Sjaelland[tw] OR Sealand[tw] OR "Zealand region"[tw] OR "region Zealand"[tw] OR Syddanmark[tw] OR Jutland[tw] OR Jylland[tw] OR Sonderjyllands[tw] OR Copenhagen[tw] OR Kobenhavn[tw] OR Arhus[tw] OR Aarhus[tw] OR Bornholm[tw] OR Frederiksberg[tw] OR Frederiksborg[tw] OR Ringkjobing[tw] OR Viborg[tw] OR Vejle[tw] OR Roskilde[tw] OR Storstrom[tw] OR Vestsjaellands[tw] OR "West Zealand"[tw] OR Funen[tw] OR Ribe[tw] OR "Kalaallit Nunaat"[tw] OR Gronland[tw] OR Foroyar[tw] OR Faeroerne[tw] OR "Faroe islands"[tw] OR Aalborg[tw] OR Alborg[tw] OR Odense[tw] OR Esbjerg[tw] OR Gentofte[tw] OR Gladsaxe[tw] OR Randers[tw] OR Kolding[tw]) OR ("Estonia"[Mesh] OR Estonia*[tw] OR Eesti[tw] OR Eestlased[tw] OR Eestlane[tw] OR Harju[tw] OR Harjumaa[tw] OR Hiiu[tw] OR Hiiumaa[tw] OR "Ida Viru"[tw] OR "Ida Virumaa"[tw] OR Jarvamaa[tw] OR Jarva[tw] OR Jogevamaa[tw] OR Jogeva[tw] OR Laanemaa[tw] OR Laane[tw] OR "Laane Virumaa"[tw] OR Parnu[tw] OR Parnumaa[tw] OR Polva[tw] OR Polvamaa[tw] OR Rapla[tw] OR Raplamaa[tw] OR Saare[tw] OR Saaremaa[tw] OR Tartu[tw] OR Tartumaa[tw] OR Valga[tw] OR Valgamaa[tw] OR Viljandimaa[tw] OR Viljandi[tw] OR Voru[tw] OR Vorumaa[tw] OR Tallinn[tw] OR Narva[tw] OR "Kohtla Jarve"[tw] OR Rakvere[tw] OR Maardu[tw] OR Sillamae[tw] OR Kuressaare[tw]) OR ("Finland"[Mesh] OR Finland[tw] OR Finnish*[tw] OR Finn[tw] OR Finns[tw] OR Suomi[tw] AND Suomen[tw] OR Suomalaiset[tw] OR Aland[tw] OR Ahvenanmaa[tw] OR Uusimaa[tw] OR Nyland[tw] OR Karelia[tw] OR Karjala[tw] OR Karelen[tw] OR Ostrobothnia[tw] OR Pohjanmaa[tw] OR Osterbotten[tw] OR Savonia[tw] OR Savo[tw] OR Savolax[tw] OR Kainuu[tw] OR Kajanaland*[tw] OR "Kanta Hame"[tw] OR Tavastia[tw] OR Tavastland[tw] OR Kymenlaakso[tw] OR Kymmenedalen[tw] OR Lapland[tw] OR Lappi[tw] OR Lappland[tw] OR "Paijat Hame"[tw] OR Pirkanmaa[tw] OR Birkaland[tw] OR Satakunta[tw] OR Satakunda[tw] OR Helsinki[tw] OR Helsingfors[tw] OR Espoo[tw] OR Esbo[tw] OR Tampere[tw] OR Tammerfors[tw] OR Vantaa[tw] OR Vanda[tw] OR Oulu[tw] OR Uleaborg[tw] OR Turku[tw] OR Abo[tw] OR Jyvaskyla[tw] OR Kuopio[tw] OR Lahti[tw] OR Lahtis[tw] OR Kouvola[tw]) OR ("France"[Mesh] OR France[tw] OR French*[tw] OR Francais*[tw] OR Alsace[tw] OR Elsass[tw] OR Aquitaine[tw] OR Aquitania[tw] OR Akitania[tw] OR Aguiene[tw] OR Auvergne[tw] OR Auvernhe[tw] OR Auvernha[tw] OR Normandie[tw] OR Normandy[tw] OR Normaundie[tw] OR Bourgogne[tw] OR Burgundy[tw] OR Bregogne[tw] OR Borgoegne[tw] OR Borgogne[tw] OR Brittany[tw] OR Breizh[tw] OR Bertaeyn[tw] OR Bretagne[tw] OR "Champagne Ardenne"[tw] OR Corse[tw] OR Corsica[tw] OR "Franche Comte"[tw] OR "Frantche Comte"[tw] OR "Franche Comtat"[tw] OR Guadeloupe[tw] OR Guyane[tw] OR Guiana[tw] OR "Languedoc Roussillon"[tw] OR "Lengadoc Rosselhon"[tw] OR "Llenguadoc-Rossello"[tw] OR Limousin[tw] OR Lemosin[tw] OR Lorraine[tw] OR Lothringen[tw] OR Lottringe[tw] OR Martinique[tw] OR "Midi Pyrenees"[tw] OR "Miegjorn Pireneus"[tw] OR "Mieidia Pireneus"[tw] OR "Mediodia Pirineos"[tw] OR "Pays de la Loire"[tw] OR "Broiou al Liger"[tw] OR Picardie[tw] OR Picardy[tw] OR "Poitou Charentes"[tw] OR "Peitau Charantas"[tw] OR "Poetou-Cherentes"[tw] OR Provence[tw] OR Provenca[tw] OR Prouvenco[tw] OR "Cote d Azur"[tw] OR "Cote d'Azur"[tw] OR "Costo d'Azur"[tw] OR "Costo d Azur"[tw] OR "Costa d'Azur"[tw] OR "Costa d Azur"[tw] OR Reunion[tw] OR "Rhone Alpes"[tw] OR "Rono Arpes"[tw] OR "Rose Aups"[tw] OR Ain[tw] OR Aisne[tw] OR Allier[tw] OR "Alpes de Haute Provence"[tw] OR "Haute Alpes"[tw] OR "Alpes Maritimes"[tw] OR Ardeche[tw] OR Ardennes[tw] OR Ariege[tw] OR Aube[tw] OR Aude[tw] OR Aveyron[tw] OR "Bas Rhin"[tw] OR "Bouches du Rhone"[tw] OR Calvados[tw] OR Cantal[tw] OR Charente[tw] OR Cher[tw] OR Correze[tw] OR "Corse du Sud"[tw] OR "Cote d Or"[tw] OR "Cote d'Or"[tw] OR "Cotes d Armor"[tw] OR "Cotes d'Armor"[tw] OR Creuse[tw] OR "Deux Sevres"[tw] OR Dordogne[tw] OR Doubs[tw] OR Drome[tw] OR Essonne[tw] OR Eure[tw] OR Finistere[tw] OR Gard[tw] OR Gers[tw] OR Gironde[tw] OR "Haute Corse"[tw] OR "Haute Garonne"[tw] OR "Haute Marne"[tw] OR "Hautes Alpes"[tw] OR "Haute Saone"[tw] OR "Haute Savoie"[tw] OR "Hautes Pyrenees"[tw] OR "Haute Vienne"[tw] OR "Haut Rhin"[tw] OR "Hauts de Seine"[tw] OR Herault[tw] OR "Ile de France"[tw] OR "Ille et Vilaine"[tw] OR Indre[tw] OR Isere[tw] OR Jura[tw] OR Landes[tw] OR Loire[tw] OR Loiret[tw] OR (Lot[tw] AND (departement[tw] OR department[tw])) OR "Lot et Garonne"[tw] OR "Loir et Cher"[tw] OR Lozere[tw] OR Manche[tw] OR Marne[tw] OR Mayenne[tw] OR Mayotte[tw] OR "Meurthe et Moselle"[tw] OR Meuse[tw] OR Morbihan[tw] OR Moselle[tw] OR (Nord[tw] AND (department[tw] OR departement[tw])) OR Nievre[tw] OR Oise[tw] OR Orne[tw] OR "Pas de calais"[tw] OR "Noord-Nauw van Kales"[tw] OR Paris[tw] OR "Puy de dome"[tw] OR "Pyrenees Atlantiques"[tw] OR "Pyrenees Orientales"[tw] OR Rhone[tw] OR Sarthe[tw] OR Savoie[tw] OR "Seine et Marne"[tw] OR "Seine Maritime"[tw] OR Somme[tw] OR Tarn[tw] OR "Territoire de Belfort"[tw] OR "Val de Marne"[tw] OR "Val d Oise"[tw] OR Var[tw] OR Vaucluse[tw] OR Vendee[tw] OR Vienne[tw] OR Vosges[tw] OR Yonne[tw] OR Yvelines[tw] OR Marseille[tw] OR Lyon[tw] OR Nice[tw] OR Nantes[tw] OR Strasbourg[tw] OR Montpellier[tw] OR Bordeaux[tw] OR Lille[tw] OR Toulouse[tw] OR "Outre Mer"[tw] OR "Seine Saint Denis"[tw]) OR ("Germany"[Mesh] OR German*[tw] OR Deutsch*[tw] OR Bundesrepublik[tw] OR Westdeutschland[tw] OR Ostdeutschland[tw] OR Baden[tw] OR Wuerttemberg[tw] OR Wurttemberg[tw] OR Bayern[tw] OR Bavaria[tw] OR Berlin[tw] OR Brandenburg[tw] OR Bremen[tw] OR Oldenburg[tw] OR Mitteldeutschland[tw] OR Rhein[tw] OR Rhine[tw] OR Hannover[tw] OR Braunschweig[tw] OR Gottingen[tw] OR Goettingen[tw] OR Nurnberg[tw] OR Nuernberg[tw] OR Ruhr[tw] OR Koln[tw] OR koeln[tw] OR Bonn[tw] OR Hamburg[tw] OR Hessen[tw] OR Hesse[tw] OR Hessia[tw] OR Mecklenburg[tw] OR Vorpommern[tw] OR Pomerania[tw] OR Niedersachsen[tw] OR Neddersassen[tw] OR Saxony[tw] OR Niederbayern[tw] OR "Northern Rhine"[tw] OR "North Rhine"[tw] OR Westphalia[tw] OR Westfalen[tw] OR "Rhineland Palatinate"[tw] OR "Rheinland Pfalz"[tw] OR Saarland[tw] OR Sachsen[tw] OR "Schleswig Holstein"[tw] OR Thuringia[tw] OR Thuringen[tw] OR Thueringen[tw] OR Munchen[tw] OR Muenchen[tw] OR Munich[tw] OR Frankfurt[tw] OR Stuttgart[tw] OR Dusseldorf[tw] OR Duesseldorf[tw] OR Dortmund[tw] OR Essen[tw]) OR ("Greece"[Mesh] OR Greece[tw] OR "Hellenic republic"[tw] OR Greek*[tw] OR Ellada[tw] OR Elladas[tw] OR "Elliniki Dimokratia"[tw] OR Hellas[tw] OR Hellenes[tw] OR Attica[tw] OR Attiki[tw] OR Makedonia*[tw] OR Macedonia[tw] OR Thraki[tw] OR Thrace[tw] OR Crete[tw] OR Kriti[tw] OR "Ionia Nisia"[tw] OR "Ionion neson"[tw] OR "Ionion nIson"[tw] OR "Ionian islands"[tw] OR "Ionian island"[tw] OR Epirus[tw] OR Ipeiros[tw] OR "Perifereia Ipeirou"[tw] OR "North aegean"[tw] OR "Northern Aegean"[tw] OR "Aegean islands"[tw] OR "Aegean island"[tw] OR "Nisoi Agaiou"[tw] OR "Notio Aigaio"[tw] OR Peloponnese[tw] OR Peloponniso*[tw] OR Thessaly[tw] OR Thessalia[tw] OR Thessalian[tw] OR Petthalia[tw] OR "Voreio Aigaio"[tw] OR "Voreio Aigaiou"[tw] OR "South aegean"[tw] OR "Southern Aegean"[tw] OR "Mount athos"[tw] OR "Oros Athos"[tw] OR Cyclades[tw] OR Cycklades[tw] OR Kiklades[tw] OR Dodecanese[tw] OR Dodekanisa[tw] OR Athens[tw] OR Athina[tw] OR Thessaloniki[tw] OR Thessalonica[tw] OR Patras[tw] OR Patra[tw] OR Pireas[tw] OR Piraeus[tw] OR Larissa[tw] OR Larisa[tw] OR Heraklion[tw] OR Heraclion[tw] OR Iraklion[tw] OR Irakleion[tw] OR Iraklio[tw] OR Volos[tw] OR Rhodes[tw] OR Rodos[tw] OR Ioannina[tw] OR Janina[tw] OR Yannena[tw] OR Chania[tw] OR Chalcis[tw] OR Chalkida[tw]) OR ("Hungary"[Mesh] OR Hungar*[tw] OR Magyarorszag[tw] OR Magyar*[tw] OR Dunantuli[tw] OR Transdanubia[tw] OR Dunantul[tw] OR "Great Plain"[tw] OR "Eszak Alfold"[tw] OR "Del Alfold"[tw] OR "Alfold es eszak"[tw] OR "Northern Alfold"[tw] OR "North Alfold"[tw] OR "South Alfold"[tw] OR "Southern Alfold"[tw] OR Bacs[tw] OR Kiskun[tw] OR Baranya[tw] OR Bekes[tw] OR Borsod[tw] OR Abauj[tw] OR Zemplen[tw] OR Budapest[tw] OR Csongrad[tw] OR Fejer[tw] OR gyor[tw] OR moson[tw] OR sopron[tw] OR hajdu[tw] OR bihar[tw] OR Heves[tw] OR "jasz nagykun szolnok"[tw] OR komarom[tw] OR esztergom[tw] OR Nograd[tw] OR (Pest[tw] AND (megye[tw] OR county[tw])) OR Somogy[tw] OR szabolcs[tw] OR szatmar[tw] OR bereg[tw] OR Tolna[tw] OR Vas[tw] OR Veszprem[tw] OR Zala[tw] OR Debrecen[tw] OR Miskolc[tw] OR Szeged[tw] OR Pecs[tw] OR Gyor[tw] OR Nyiregyhaza[tw] OR Kecskemet[tw] OR Szekesfehervar[tw] OR Szombathely[tw]) OR ("Ireland"[Mesh] OR Ireland[tw] OR Eire[tw] OR Irish*[tw] OR Fingal[tw] OR "Fine Gall"[tw] OR Dublin[tw] OR "Ath Cliath"[tw] OR "Dun Laoghaire"[tw] OR Wicklow[tw] OR "Cill Mhantain"[tw] OR "Chill Mhantain"[tw] OR Wexford[tw] OR "Loch Garman"[tw] OR Carlow[tw] OR Ceatharlach[tw] OR Kildare[tw] OR "Cill Dara"[tw] OR "Chill Dara"[tw] OR Meath[tw] OR "An Mhi"[tw] OR "Contae na Mi"[tw] OR Louth[tw] OR "Contae Lu"[tw] OR Monaghan[tw] OR Muineachan[tw] OR Mhuineachain[tw] OR Cavan[tw] OR "An Cabhan"[tw] OR "An Cabhain"[tw] OR Longford[tw] OR "An Longfort"[tw] OR "an Longfoirt"[tw] OR Langfurd[tw] OR Westmeath[tw] OR "An Iarmhi"[tw] OR "na Iarmhi"[tw] OR Offaly[tw] OR "Uibh Fhaili"[tw] OR Laois[tw] OR Laoise[tw] OR Kilkenny[tw] OR "Chill Chainnigh"[tw] OR "Cill Chainnigh"[tw] OR Waterford[tw] OR "Port Lairge"[tw] OR Watterford[tw] OR Cork[tw] OR Corcaigh[tw] OR Chorcai[tw] OR Kerry[tw] OR Ciarrai[tw] OR Chiarrai[tw] OR Limerick[tw] OR Luimneach[tw] OR Luimnigh[tw] OR Tipperary[tw] OR "Tiobraid Arann"[tw] OR "Thiobraid Arann"[tw] OR Clare[tw] OR "An Clar"[tw] OR "an Chlair"[tw] OR Galway[tw] OR Gaillimh[tw] OR "na Gaillimhe"[tw] OR Mayo[tw] OR "Maigh Eo"[tw] OR "Mhaigh Eo"[tw] OR Roscommon[tw] OR "Ros comain"[tw] OR Sligo[tw] OR Sligeach[tw] OR Shligigh[tw] OR Leitrim[tw] OR Liatroim[tw] OR Liatroma[tw] OR Donegal[tw] OR "Dhun na nGall"[tw] OR Dinnygal[tw] OR Dunnyga[tw] OR Leinster[tw] OR Laighin[tw] OR "Cuige Laighean"[tw] OR Munster[tw] OR Mumhain[tw] OR "Cuige Mumhan"[tw] OR Connacht[tw] OR Connachta[tw] OR Drogheda[tw] OR "Droichead Atha"[tw] OR Dundalk[tw] OR "Dun Dealgan"[tw] OR Swords[tw] OR Sord[tw] OR Bray[tw] OR Bre[tw] OR Navan[tw] OR "An Uaimh"[tw]) OR ("Italy"[Mesh] OR Italy[tw] OR Italia*[tw] OR Abruzzo[tw] OR Abruzzi[tw] OR Basilicata[tw] OR Lucania[tw] OR Calabria[tw] OR Campania[tw] OR "Emilia Romagna"[tw] OR "friuli venezia giulia"[tw] OR Lazio[tw] OR Latium[tw] OR Liguria*[tw] OR Lombardy[tw] OR Lombardia[tw] OR Marche[tw] OR Marches[tw] OR Molisano[tw] OR Molise[tw] OR Piedmont*[tw] OR Piemonte[tw] OR Bolzano[tw] OR Bozen[tw] OR Trentino[tw] OR Trento[tw] OR Puglia[tw] OR Apulia[tw] OR Sardinia[tw] OR Sardegna[tw] OR Sicily[tw] OR Sicilia[tw] OR Toscana[tw] OR Tuscany[tw] OR Umbria[tw] OR "Valle d Aosta"[tw] OR "Vallee d Aoste"[tw] OR "Valle d'Aosta"[tw] OR "Vallee d'Aoste"[tw] OR "Aosta Valley"[tw] OR Veneto[tw] OR Venetia[tw] OR Triveneto[tw] OR Rome[tw] OR Roma[tw] OR Milan[tw] OR Milano[tw] OR Naples[tw] OR Napoli[tw] OR Turin[tw] OR Torino[tw] OR Palermo[tw] OR Genoa[tw] OR Genova[tw] OR Bologna[tw] OR Florence[tw] OR Firenze[tw] OR Bari[tw] OR Catania[tw]) OR ("Latvia"[Mesh] OR Latvi*[tw] OR Riga[tw] OR Courland[tw] OR Kurzeme[tw] OR Kurland[tw] OR Latgale[tw] OR Lettgallia[tw] OR Latgola[tw] OR Latgalia[tw] OR Vidzeme[tw] OR Vidumo[tw] OR Semigallia[tw] OR Semigalia[tw] OR Zemgale[tw] OR Pieriga[tw] OR Daugavpils[tw] OR Dinaburg[tw] OR Jekabpils[tw] OR Jakobstadt[tw] OR Jelgava[tw] OR Jurmala[tw] OR Liepaja[tw] OR Libau[tw] OR Rezekne[tw] OR Rezne[tw] OR Rositten[tw] OR Valmiera[tw] OR Wolmar[tw] OR Ventspils[tw] OR Windau[tw] OR Ogre[tw]) OR ("Lithuania"[Mesh] OR Lithuania*[tw] OR "Lietuvos Respublika"[tw] OR Lietuva[tw] OR lietuviu[tw] OR Alytus[tw] OR Alytaus[tw] OR Kaunas[tw] OR Kauno[tw] OR Klaipeda[tw] OR Klaipedos[tw] OR Marijampoles[tw] OR Marijampole[tw] OR Panevezys[tw] OR Panevezio[tw] OR Siauliai[tw] OR Siauliu[tw] OR Taurages[tw] OR Taurage[tw] OR Telsiu[tw] OR Telsiai[tw] OR Utenos[tw] OR Utena[tw] OR Vilnius[tw] OR Vilniaus[tw] OR Mazeikiai[tw] OR Jonava[tw] OR Mazeikiu[tw] OR Jonavos[tw]) OR ("Luxembourg"[Mesh] OR Luxembourg*[tw] OR Luxemburg[tw] OR Letzebuerg[tw] OR Diekirch[tw] OR Grevenmacher[tw] OR "Esch sur Alzette"[tw] OR "Esch Uelzecht"[tw] OR "Esch an der Alzette"[tw] OR "Esch an der Alzig"[tw] OR Dudelange[tw] OR Diddeleng[tw] OR Dudelingen[tw] OR Duedelingen[tw] OR Schifflange[tw] OR Scheffleng[tw] OR Schifflingen[tw] OR Bettembourg[tw] OR Beetebuerg[tw] OR Bettemburg[tw] OR Petange[tw] OR Peiteng[tw] OR Petingen[tw] OR Ettelbruck[tw] OR Ettelbreck[tw] OR Ettelbrueck[tw] OR Diekirch[tw] OR Dikrech[tw] OR Strassen[tw] OR Stroossen[tw] OR Bertrange[tw] OR Bartreng[tw] OR Bartringen[tw]) OR ("Malta"[Mesh] OR Malta[tw] OR Maltese*[tw] OR Maltin[tw] OR Gozo[tw] OR Ghawdex[tw] OR Valletta[tw] OR "Ill Belt"[tw] OR Birkirkara[tw] OR "B Kara"[tw] OR "B'Kara"[tw] OR Birchircara[tw] OR Mosta[tw] OR Qormi[tw] OR "St Paul s Bay"[tw] OR "St Paul's Bay"[tw] OR "Pawl il Bahar"[tw] OR Zabbar[tw] OR Sliema[tw] OR Naxxar[tw] OR Gwann[tw] OR "St John"[tw] OR Zebbug[tw] OR "Citta rohan"[tw] OR Fgura[tw]) OR ("Netherlands"[Mesh] OR Netherlands[tw] OR Nederland*[tw] OR Dutch*[tw] OR Drenthe[tw] OR Flevoland[tw] OR Friesland[tw] OR Fryslan[tw] OR Frisia[tw] OR Gelderland[tw] OR Guelders[tw] OR Groningen[tw] OR Limburg[tw] OR Brabant[tw] OR Holland[tw] OR Overijssel[tw] OR Overissel[tw] OR Utrecht[tw] OR Zeeland[tw] OR Amsterdam[tw] OR Rotterdam[tw] OR Hague[tw] OR "s-Gravenhage"[tw] OR "Den Haag"[tw] OR Eindhoven[tw] OR Tilburg[tw] OR Almere[tw] OR Breda[tw] OR Nijmegen[tw] OR Nimeguen[tw]) OR ("Poland"[Mesh] OR Poland[tw] OR Polska[tw] OR Polish[tw] OR Pole[tw] OR Poles[tw] OR Polski[tw] OR Polak[tw] OR Polka[tw] OR Polacy[tw] OR Dolnoslaskie[tw] OR Silesia*[tw] OR Slask[tw] OR Pomorskie[tw] OR Pomerania*[tw] OR Kujawsko[tw] OR Kuyavian[tw] OR Lodzkie[tw] OR Lodz[tw] OR Lubelskie[tw] OR Lublin[tw] OR Lubuskie[tw] OR Lubusz[tw] OR Lubus[tw] OR Malopolskie[tw] OR Mazowieckie[tw] OR Mazowske[tw] OR Masovia[tw] OR Masovian[tw] OR Opolskie[tw] OR Opole[tw] OR Podkarpackie[tw] OR Subcarpathian*[tw] OR Podlaskie[tw] OR Podlachia[tw] OR Podlasie[tw] OR Slaskie[tw] OR Swietokrzyskie[tw] OR "Varmia Mazuria"[tw] OR "Varmian Mazurian"[tw] OR "Varmia Masuria"[tw] OR "Varmian Masurian"[tw] OR "Warmia Mazury"[tw] OR "Warminsko Mazurskie"[tw] OR "Warmian Masurian"[tw] OR Wielkopolskie[tw] OR Zachodniopomorskie[tw] OR Warsaw[tw] OR Warszawa[tw] OR Krakow[tw] OR Cracow[tw] OR Wroclaw[tw] OR Poznan[tw] OR Gdansk[tw] OR Szczecin[tw] OR Bydgoszcz[tw] OR Katowice[tw]) OR ("Portugal"[Mesh] OR Portugal[tw] OR Portugues*[tw] OR Azores[tw] OR Acores[tw] OR Madeira[tw] OR Alentejo[tw] OR Algarve[tw] OR Lisboa[tw] OR Lisbon[tw] OR "Alto Tras-os-Montes"[tw] OR (Ave[tw] AND (community[tw] OR intermunicipal[tw] OR comunidade[tw])) OR Mondego[tw] OR Vouga[tw] OR Beira[tw] OR Cavado[tw] OR Lafoes[tw] OR Douro[tw] OR Porto[tw] OR Oporto[tw] OR Tejo[tw] OR Minho[tw] OR Setubal[tw] OR Pinhal[tw] OR "Serra da Estrela"[tw] OR Tamega[tw] OR Leira[tw] OR Santarem[tw] OR Beja[tw] OR Faro[tw] OR Evora[tw] OR Portalegre[tw] OR "Castelo Branco"[tw] OR Guarda[tw] OR Cimbra[tw] OR Aveiro[tw] OR Viseu[tw] OR Braganca[tw] OR Braganza[tw] OR Braga[tw] OR "Vila real"[tw] OR "Viana do Castelo"[tw] OR Gaia[tw] OR Amadora[tw] OR Funchal[tw] OR Coimbra[tw] OR Almada[tw] OR (Agualva[tw] AND Cacem[tw])) OR ("Romania"[Mesh] OR Romania*[tw] OR Rumania*[tw] OR Roumania*[tw] OR Romani[tw] OR Rumani[tw] OR Alba[tw] OR Arad[tw] OR Arges[tw] OR Bacau[tw] OR Bihor[tw] OR "Bistrita Nasaud"[tw] OR Botosani[tw] OR Braila[tw] OR Brasov[tw] OR Kronstadt[tw] OR Brasso[tw] OR Brassovia[tw] OR Coron[tw] OR Bucharest[tw] OR Bucuresti[tw] OR Buzau[tw] OR Calarasi[tw] OR "Caras-Severin"[tw] OR Cluj[tw] OR Klausenburg[tw] OR Kolozsvar[tw] OR Constanta[tw] OR Tomis[tw] OR Konstantia[tw] OR Kostence[tw] OR Covasna[tw] OR Dambovita[tw] OR Dolj[tw] OR Galati[tw] OR Galatz[tw] OR Galac[tw] OR Kalas[tw] OR Giurgiu[tw] OR Gorj[tw] OR Harghita[tw] OR Hunedoara[tw] OR Ialomita[tw] OR Iasi[tw] OR Jassy[tw] OR Lassy[tw] OR Ilfov[tw] OR Maramures[tw] OR Mehedinti[tw] OR Mures[tw] OR Neamt[tw] OR (Olt[tw] AND (river[tw] OR county[tw] OR region[tw] OR judetul[tw] OR Raul[tw])) OR Prahova[tw] OR Salaj[tw] OR "Satu Mare"[tw] OR Sibiu[tw] OR Suceava[tw] OR Teleorman[tw] OR Timis[tw] OR Tulcea[tw] OR Valcea[tw] OR Vilcea[tw] OR Vaslui[tw] OR Vrancea[tw] OR Timisoara[tw] OR Temeswar[tw] OR Temeschburg[tw] OR Temeschwar[tw] OR Temesvar[tw] OR Temisvar[tw] OR Timisvar[tw] OR Temesva[tw] OR Craiova[tw] OR Ploiesti[tw] OR Ploesti[tw] OR Oradea[tw] OR Varad[tw] OR Varat[tw]) OR ("Slovakia"[Mesh] OR Slovakia[tw] OR Slovensk*[tw] OR Slovak*[tw] OR Slovaci[tw] OR Slovenki[tw] OR Bratislav*[tw] OR Presporok[tw] OR Pressburg[tw] OR Pressburg[tw] OR Posonium[tw] OR Banskobystri*[tw] OR "Banska Bystrica"[tw] OR Neusohl[tw] OR Besztercebanya[tw] OR Kosic*[tw] OR Kaschau[tw] OR Kassa[tw] OR Nitrian*[tw] OR Nitra[tw] OR Neutra[tw] OR Nyitra[tw] OR Nyitria[tw] OR Trnav*[tw] OR Tyrnau[tw] OR Nagyszombat[tw] OR Tyrnavia[tw] OR Presov*[tw] OR Trencian*[tw] OR Trencin[tw] OR Trentschin[tw] OR Trencsen[tw] OR Zilina[tw] OR Sillein[tw] OR Zsolna[tw] OR Zylina[tw] OR (Martin[tw] AND (city[tw] OR Svaty[tw])) OR Turocszentmarton[tw] OR Poprad[tw] OR Deutschendorf[tw] OR Zvolen[tw]) OR ("Slovenia"[Mesh] OR Slovenia*[tw] OR Slovenija[tw] OR slovensk*[tw] OR Slovenci[tw] OR Slovene*[tw] OR Gorenjska[tw] OR Carniola[tw] OR Goriska[tw] OR Gorizia[tw] OR Jugovzhodna[tw] OR Koroska[tw] OR Carinthia[tw] OR "Notranjsko kraska"[tw] OR "Obalno kraska"[tw] OR "Coastal karst"[tw] OR Osrednjeslovenska[tw] OR Podravska[tw] OR Drava[tw] OR Pomurska[tw] OR Mura[tw] OR Savinjska[tw] OR Savinja[tw] OR Spodnjeposavska[tw] OR Zasavska[tw] OR "Central Sava"[tw] OR Posavska[tw] OR "Lower Sava"[tw] OR Ljubljana[tw] OR Laibach[tw] OR Lubiana[tw] OR Maribor[tw] OR "Marburg an der Drau"[tw] OR Kranj[tw] OR Carnium[tw] OR Creina[tw] OR Chreina[tw] OR Krainbur[tw] OR Koper[tw] OR Capodistria[tw] OR Kopar[tw] OR Celje[tw] OR "Novo mesto"[tw] OR Neustadtl[tw] OR Domzale[tw] OR Velenje[tw] OR Wollan[tw] OR Woellan[tw] OR "Nova Gorica"[tw] OR Kamnik[tw]) OR ("Spain"[Mesh] OR Spain[tw] OR Espana[tw] OR Spanish[tw] OR Espanol*[tw] OR Spaniard*[tw] OR Andalucia[tw] OR Andalusia[tw] OR Aragon[tw] OR Arago[tw] OR Cantabria[tw] OR Canarias[tw] OR "Canary Islands"[tw] OR (Canaries[tw] AND island*[tw]) OR "Castile and leon"[tw] OR "Castilla y Leon"[tw] OR "Castile La Mancha"[tw] OR "Castilla La Mancha"[tw] OR Cataluna[tw] OR Catalonia[tw] OR Ceuta[tw] OR Madrid[tw] OR Melilla[tw] OR Navarra[tw] OR Navarre[tw] OR Valencia*[tw] OR Extremadura[tw] OR Galicia[tw] OR Balears[tw] OR "Balearic Islands"[tw] OR "Balear Islands"[tw] OR Baleares[tw] OR "La Rioja"[tw] OR "Pais Vasco"[tw] OR "Basque Country"[tw] OR "Baske region"[tw] OR Euskadi[tw] OR Asturias[tw] OR Murcia[tw] OR Coruna[tw] OR Alava[tw] OR Araba[tw] OR Albacete[tw] OR Alicante[tw] OR Alacant[tw] OR Almeria[tw] OR Avila[tw] OR Badajoz[tw] OR Badajos[tw] OR Barcelona[tw] OR Burgos[tw] OR Caceres[tw] OR Cadiz[tw] OR Castellon[tw] OR Castello[tw] OR "Ciudad Real"[tw] OR Cordoba[tw] OR Cuenca[tw] OR Eivissa[tw] OR Ibiza[tw] OR Formentera[tw] OR "El Hierro"[tw] OR Fuerteventura[tw] OR Galiza[tw] OR Girona[tw] OR Gerona[tw] OR "Gran Canaria"[tw] OR Granada[tw] OR Guadalajara[tw] OR Guipuzcoa[tw] OR Gipuzkoa[tw] OR Huelva[tw] OR Huesca[tw] OR Jaen[tw] OR "La Gomera"[tw] OR "La Palma"[tw] OR Lanzarote[tw] OR Leon[tw] OR Lleida[tw] OR Lerida[tw] OR Lugo[tw] OR Malaga[tw] OR Mallorca[tw] OR Majorca[tw] OR Menorca[tw] OR Minorca[tw] OR Murcia[tw] OR Ourense[tw] OR Orense[tw] OR Palencia[tw] OR Pontevedra[tw] OR Salamanca[tw] OR Segovia[tw] OR Sevilla[tw] OR Seville[tw] OR Soria[tw] OR Tarragona[tw] OR Tenerife[tw] OR Teruel[tw] OR Toledo[tw] OR Valladolid[tw] OR Vizcaya[tw] OR Biscay[tw] OR Zamora[tw] OR Zaragoza[tw] OR Saragossa[tw] OR "Las Palmas"[tw] OR Bilbao[tw] OR Bilbo[tw]) OR ("Sweden"[Mesh] OR Sweden[tw] OR Sverige[tw] OR Swedish[tw] OR Svenska[tw] OR svenskar[tw] OR Swede[tw] OR Swedes[tw] OR Norrland[tw] OR Mellansverige[tw] OR Smaland[tw] OR Stockholm*[tw] OR Sydsverige[tw] OR Vastsverige[tw] OR Blekinge[tw] OR Dalarna[tw] OR Gavleborg*[tw] OR Gotland*[tw] OR Halland*[tw] OR Jamtland*[tw] OR Jonkoping*[tw] OR Kalmar[tw] OR Kronoberg*[tw] OR Norrbotten*[tw] OR Orebro[tw] OR Ostergotland*[tw] OR Skane[tw] OR Sodermanlands[tw] OR Uppsala[tw] OR Varmland*[tw] OR Vasterbotten*[tw] OR Vasternorrland*[tw] OR Vastmanland*[tw] OR vastergotland*[tw] OR Gotaland*[tw] OR Gothenburg[tw] OR Goteborg[tw] OR Malmo[tw] OR Vasteras[tw] OR Linkoping[tw] OR Helsingborg[tw] OR Halsingborg[tw] OR Norrkoping[tw]) OR ("Great Britain"[Mesh] OR GB[tw] OR "United kingdom"[tw] OR UK[tw] OR Britain[tw] OR British[tw] OR England[tw] OR English[tw] OR Scotland[tw] OR Scottish[tw] OR Scots[tw] OR Wales[tw] OR Cymru[tw] OR Welsh[tw] OR Irish[tw] OR Avon[tw] OR Bedfordshire[tw] OR Berkshire[tw] OR Bristol[tw] OR Buckinghamshire[tw] OR Cambridgeshire[tw] OR "Isle of Ely"[tw] OR Cheshire[tw] OR Cleveland[tw] OR Cornwall[tw] OR Cumberland[tw] OR Cumbria[tw] OR Derbyshire[tw] OR Devon[tw] OR Dorset[tw] OR Durham[tw] OR Essex[tw] OR Gloucestershire[tw] OR Hampshire[tw] OR Southampton[tw] OR (Hereford[tw] AND Worcester[tw]) OR Hertfordshire[tw] OR Herefordshire[tw] OR Humberside[tw] OR Huntingdon[tw] OR Huntingdonshire[tw] OR "Isle of Wight"[tw] OR Kent[tw] OR Lancashire[tw] OR Leicestershire[tw] OR Lincolnshire[tw] OR London[tw] OR Manchester[tw] OR Merseyside[tw] OR Middlesex[tw] OR Norfolk[tw] OR Northamptonshire[tw] OR Northumberland[tw] OR Nottinghamshire[tw] OR Oxfordshire[tw] OR Peterborough[tw] OR Rutland[tw] OR Shropshire[tw] OR Salop[tw] OR Somerset[tw] OR Yorkshire[tw] OR Staffordshire[tw] OR Suffolk[tw] OR Surrey[tw] OR Sussex[tw] OR (Tyne[tw] AND Wear[tw]) OR Warwickshire[tw] OR Midlands[tw] OR Westmorland[tw] OR Wiltshire[tw] OR Worcestershire[tw] OR "Isle of Man"[tw] OR Jersey[tw] OR Guernsey[tw] OR "Channel Islands"[tw] OR Aberdeen[tw] OR Aberdeenshire[tw] OR Angus[tw] OR Forfarshire[tw] OR Argyll[tw] OR Ayrshire[tw] OR Banffshire[tw] OR Berwickshire[tw] OR Bute[tw] OR Caithness[tw] OR Clackmannanshire[tw] OR Cromartyshire[tw] OR Dumfriesshire[tw] OR Dunbartonshire[tw] OR Dumbarton[tw] OR Dundee[tw] OR Lothian[tw] OR Haddingtonshire[tw] OR Edinburgh[tw] OR Fife[tw] OR Glasgow[tw] OR Inverness-shire[tw] OR Kincardineshire[tw] OR Kinross-shire[tw] OR Kirkcudbrightshire[tw] OR Lanarkshire[tw] OR Midlothian[tw] OR Moray[tw] OR Elginshire[tw] OR Nairnshire[tw] OR Orkney[tw] OR Peeblesshire[tw] OR Perthshire[tw] OR Renfrewshire[tw] OR (Ross[tw] AND Cromarty[tw]) OR Ross-shire[tw] OR Roxburghshire[tw] OR Selkirkshire[tw] OR Shetland[tw] OR Zetland[tw] OR Stirlingshire[tw] OR Sutherland[tw] OR Linlithgowshire[tw] OR Wigtownshire[tw] OR Anglesey[tw] OR Brecknockshire[tw] OR Caernarfonshire[tw] OR Carmarthenshire[tw] OR Cardiganshire[tw] OR Ceredigion[tw] OR Clwyd[tw] OR Denbighshire[tw] OR Dyfed[tw] OR Flintshire[tw] OR Glamorgan[tw] OR Gwent[tw] OR Gwynedd[tw] OR Merionethshire[tw] OR Montgomeryshire[tw] OR Monmouthshire[tw] OR Pembrokeshire[tw] OR Powys[tw] OR Radnorshire[tw] OR Antrim[tw] OR Aontroim[tw] OR "Contae Aontroma"[tw] OR Anthrim[tw] OR Antrim[tw] OR Entrim[tw] OR Armagh[tw] OR "Ard Mhacha"[tw] OR Airmagh[tw] OR Belfast[tw] OR (Down[tw] AND (district[tw] OR council[tw] OR County[tw])) OR "An Dun"[tw] OR "an Duin"[tw] OR Doon[tw] OR Doun[tw] OR Fermanagh[tw] OR "Fear Manach"[tw] OR "Fhear Manach"[tw] OR Fermanay[tw] OR Londonderry[tw] OR Doire[tw] OR Dhoire[tw] OR Lunnonderrie[tw] OR Derry[tw] OR Birmingham[tw] OR Leeds[tw] OR Sheffield[tw] OR Bradford[tw] OR Liverpool[tw]))) OR ((GB[ad] OR "United kingdom"[ad] OR UK[ad] OR Britain[ad] OR British[ad] OR England[ad] OR English[ad] OR Scotland[ad] OR Scottish[ad] OR Scots[ad] OR Wales[ad] OR Cymru[ad] OR Welsh[ad] OR "North Ireland"[ad] OR "Northern Ireland"[ad] OR Irish[ad] OR Avon[ad] OR Bedfordshire[ad] OR Berkshire[ad] OR Bristol[ad] OR Buckinghamshire[ad] OR Cambridgeshire[ad] OR "Isle of Ely"[ad] OR Cheshire[ad] OR Cleveland[ad] OR Cornwall[ad] OR Cumberland[ad] OR Cumbria[ad] OR Derbyshire[ad] OR Devon[ad] OR Dorset[ad] OR Durham[ad] OR Essex[ad] OR Gloucestershire[ad] OR Hampshire[ad] OR Southampton[ad] OR (Hereford[ad] AND Worcester[ad]) OR Hertfordshire[ad] OR Herefordshire[ad] OR Humberside[ad] OR Huntingdon[ad] OR Huntingdonshire[ad] OR "Isle of Wight"[ad] OR Kent[ad] OR Lancashire[ad] OR Leicestershire[ad] OR Lincolnshire[ad] OR London[ad] OR Manchester[ad] OR Merseyside[ad] OR Middlesex[ad] OR Norfolk[ad] OR Northamptonshire[ad] OR Northumberland[ad] OR Nottinghamshire[ad] OR Oxfordshire[ad] OR Peterborough[ad] OR Rutland[ad] OR Shropshire[ad] OR Salop[ad] OR Somerset[ad] OR Yorkshire[ad] OR Staffordshire[ad] OR Suffolk[ad] OR Surrey[ad] OR Sussex[ad] OR (Tyne[ad] AND Wear[ad]) OR Warwickshire[ad] OR midlands[ad] OR Westmorland[ad] OR Wiltshire[ad] OR Worcestershire[ad] OR "Isle of Man"[ad] OR Jersey[ad] OR Guernsey[ad] OR "Channel Islands"[ad] OR Aberdeen[ad] OR Aberdeenshire[ad] OR Angus[ad] OR Forfarshire[ad] OR Argyll[ad] OR Ayrshire[ad] OR Banffshire[ad] OR Berwickshire[ad] OR bute[ad] OR Caithness[ad] OR Clackmannanshire[ad] OR Cromartyshire[ad] OR Dumfriesshire[ad] OR Dunbartonshire[ad] OR Dumbarton[ad] OR Dundee[ad] OR Lothian[ad] OR Haddingtonshire[ad] OR Edinburgh[ad] OR Fife[ad] OR Glasgow[ad] OR Inverness-shire[ad] OR Kincardineshire[ad] OR Kinross-shire[ad] OR Kirkcudbrightshire[ad] OR Lanarkshire[ad] OR Midlothian[ad] OR Moray[ad] OR Elginshire[ad] OR Nairnshire[ad] OR Orkney[ad] OR Peeblesshire[ad] OR Perthshire[ad] OR Renfrewshire[ad] OR (Ross[ad] AND Cromarty[ad]) OR Ross-shire[ad] OR Roxburghshire[ad] OR Selkirkshire[ad] OR Shetland[ad] OR Zetland[ad] OR Stirlingshire[ad] OR Sutherland[ad] OR Linlithgowshire[ad] OR Wigtownshire[ad] OR Anglesey[ad] OR Brecknockshire[ad] OR Caernarfonshire[ad] OR Carmarthenshire[ad] OR Cardiganshire[ad] OR Ceredigion[ad] OR Clwyd[ad] OR Denbighshire[ad] OR Dyfed[ad] OR Flintshire[ad] OR Glamorgan[ad] OR Gwent[ad] OR Gwynedd[ad] OR Merionethshire[ad] OR Montgomeryshire[ad] OR Monmouthshire[ad] OR Pembrokeshire[ad] OR Powys[ad] OR Radnorshire[ad] OR Antrim[ad] OR Aontroim[ad] OR "Contae Aontroma"[ad] OR Anthrim[ad] OR Antrim[ad] OR Entrim[ad] OR Armagh[ad] OR "Ard Mhacha"[ad] OR Airmagh[ad] OR Belfast[ad] OR (Down[ad] AND (district[ad] OR council[ad] OR County[ad])) OR "An Dun"[ad] OR "an Duin"[ad] OR Doon[ad] OR Doun[ad] OR Fermanagh[ad] OR "Fear Manach"[ad] OR "Fhear Manach"[ad] OR Fermanay[ad] OR Londonderry[ad] OR Doire[ad] OR Dhoire[ad] OR Lunnonderrie[ad] OR Derry[ad] OR Birmingham[ad] OR Leeds[ad] OR Sheffield[ad] OR Bradford[ad] OR Liverpool[ad]) OR (Sweden[ad] OR Sverige[ad] OR Swedish[ad] OR Svenska[ad] OR svenskar[ad] OR Swede[ad] OR Swedes[ad] OR Norrland[ad] OR Mellansverige[ad] OR Smaland[ad] OR Stockholm*[ad] OR Sydsverige[ad] OR Vastsverige[ad] OR Blekinge[ad] OR Dalarna[ad] OR Gavleborg*[ad] OR Gotland*[ad] OR Halland*[ad] OR Jamtland*[ad] OR Jonkoping*[ad] OR Kalmar[ad] OR Kronoberg*[ad] OR Norrbotten*[ad] OR Orebro[ad] OR Ostergotland*[ad] OR Skane[ad] OR Sodermanlands[ad] OR Uppsala[ad] OR Varmland*[ad] OR Vasterbotten*[ad] OR Vasternorrland*[ad] OR Vastmanland*[ad] OR vastergotland*[ad] OR Gotaland*[ad] OR Gothenburg[ad] OR Goteborg[ad] OR Malmo[ad] OR Vasteras[ad] OR Linkoping[ad] OR Helsingborg[ad] OR Halsingborg[ad] OR Norrkoping[ad]) OR (Spain[ad] OR Espana[ad] OR Spanish[ad] OR Espanol*[ad] OR Spaniard*[ad] OR Andalucia[ad] OR Andalusia[ad] OR Aragon[ad] OR Arago[ad] OR Cantabria[ad] OR Canarias[ad] OR "Canary Islands"[ad] OR (Canaries[ad] AND island*[ad]) OR "Castile and leon"[ad] OR "Castilla y Leon"[ad] OR "Castile La Mancha"[ad] OR "Castilla La Mancha"[ad] OR Cataluna[ad] OR Catalonia[ad] OR Ceuta[ad] OR Madrid[ad] OR Melilla[ad] OR Navarra[ad] OR Navarre[ad] OR Valencia*[ad] OR Extremadura[ad] OR Galicia[ad] OR Balears[ad] OR "Balearic Islands"[ad] OR "Balear Islands"[ad] OR Baleares[ad] OR "La Rioja"[ad] OR "Pais Vasco"[ad] OR "Basque Country"[ad] OR "Baske region"[ad] OR Euskadi[ad] OR Asturias[ad] OR Murcia[ad] OR Coruna[ad] OR Alava[ad] OR Araba[ad] OR Albacete[ad] OR Alicante[ad] OR Alacant[ad] OR Almeria[ad] OR Avila[ad] OR Badajoz[ad] OR Badajos[ad] OR Barcelona[ad] OR Burgos[ad] OR Caceres[ad] OR Cadiz[ad] OR Castellon[ad] OR Castello[ad] OR "Ciudad Real"[ad] OR Cordoba[ad] OR Cuenca[ad] OR Eivissa[ad] OR Ibiza[ad] OR Formentera[ad] OR "El Hierro"[ad] OR Fuerteventura[ad] OR Galiza[ad] OR Girona[ad] OR Gerona[ad] OR "Gran Canaria"[ad] OR Granada[ad] OR Guadalajara[ad] OR Guipuzcoa[ad] OR Gipuzkoa[ad] OR Huelva[ad] OR Huesca[ad] OR Jaen[ad] OR "La Gomera"[ad] OR "La Palma"[ad] OR Lanzarote[ad] OR Leon[ad] OR Lleida[ad] OR Lerida[ad] OR Lugo[ad] OR Malaga[ad] OR Mallorca[ad] OR Majorca[ad] OR Menorca[ad] OR Minorca[ad] OR Murcia[ad] OR Ourense[ad] OR Orense[ad] OR Palencia[ad] OR Pontevedra[ad] OR Salamanca[ad] OR Segovia[ad] OR Sevilla[ad] OR Seville[ad] OR Soria[ad] OR Tarragona[ad] OR Tenerife[ad] OR Teruel[ad] OR Toledo[ad] OR Valladolid[ad] OR Vizcaya[ad] OR Biscay[ad] OR Zamora[ad] OR Zaragoza[ad] OR Saragossa[ad] OR "Las Palmas"[ad] OR Bilbao[ad] OR Bilbo[ad]) OR (Slovenia*[ad] OR Slovenija[ad] OR slovensk*[ad] OR Slovenci[ad] OR Slovene*[ad] OR Gorenjska[ad] OR Carniola[ad] OR Goriska[ad] OR Gorizia[ad] OR Jugovzhodna[ad] OR Koroska[ad] OR Carinthia[ad] OR "Notranjsko kraska"[ad] OR "Obalno kraska"[ad] OR "Coastal karst"[ad] OR Osrednjeslovenska[ad] OR Podravska[ad] OR Drava[ad] OR Pomurska[ad] OR Mura[ad] OR Savinjska[ad] OR Savinja[ad] OR Spodnjeposavska[ad] OR Zasavska[ad] OR "Central Sava"[ad] OR Posavska[ad] OR "Lower Sava"[ad] OR Ljubljana[ad] OR Laibach[ad] OR Lubiana[ad] OR Maribor[ad] OR "Marburg an der Drau"[ad] OR Kranj[ad] OR Carnium[ad] OR Creina[ad] OR Chreina[ad] OR Krainbur[ad] OR Koper[ad] OR Capodistria[ad] OR Kopar[ad] OR Celje[ad] OR "Novo mesto"[ad] OR Neustadtl[ad] OR Domzale[ad] OR Velenje[ad] OR Wollan[ad] OR Woellan[ad] OR "Nova Gorica"[ad] OR Kamnik[ad]) OR (Slovakia[ad] OR Slovensk*[ad] OR Slovak*[ad] OR Slovaci[ad] OR Slovenki[ad] OR Bratislav*[ad] OR Presporok[ad] OR Pressburg[ad] OR Pressburg[ad] OR Posonium[ad] OR Banskobystri*[ad] OR "Banska Bystrica"[ad] OR Neusohl[ad] OR Besztercebanya[ad] OR Kosic*[ad] OR Kaschau[ad] OR Kassa[ad] OR Nitrian*[ad] OR Nitra[ad] OR Neutra[ad] OR Nyitra[ad] OR Nyitria[ad] OR Trnav*[ad] OR Tyrnau[ad] OR Nagyszombat[ad] OR Tyrnavia[ad] OR Presov*[ad] OR Trencian*[ad] OR Trencin[ad] OR Trentschin[ad] OR Trencsen[ad] OR Zilina[ad] OR Sillein[ad] OR Zsolna[ad] OR Zylina[ad] OR (Martin[ad] AND (city[ad] OR Svaty[ad])) OR Turocszentmarton[ad] OR Poprad[ad] OR Deutschendorf[ad] OR Zvolen[ad]) OR (Romania*[ad] OR Rumania*[ad] OR Roumania*[ad] OR Romani[ad] OR Rumani[ad] OR Alba[ad] OR Arad[ad] OR Arges[ad] OR Bacau[ad] OR Bihor[ad] OR "Bistrita Nasaud"[ad] OR Botosani[ad] OR Braila[ad] OR Brasov[ad] OR Kronstadt[ad] OR Brasso[ad] OR Brassovia[ad] OR Coron[ad] OR Bucharest[ad] OR Bucuresti[ad] OR Buzau[ad] OR Calarasi[ad] OR "Caras-Severin"[ad] OR Cluj[ad] OR Klausenburg[ad] OR Kolozsvar[ad] OR Constanta[ad] OR Tomis[ad] OR Konstantia[ad] OR Kostence[ad] OR Covasna[ad] OR Dambovita[ad] OR Dolj[ad] OR Galati[ad] OR Galatz[ad] OR Galac[ad] OR Kalas[ad] OR Giurgiu[ad] OR Gorj[ad] OR Harghita[ad] OR Hunedoara[ad] OR Ialomita[ad] OR Iasi[ad] OR Jassy[ad] OR Lassy[ad] OR Ilfov[ad] OR Maramures[ad] OR Mehedinti[ad] OR Mures[ad] OR Neamt[ad] OR (Olt[ad] AND (river[ad] OR county[ad] OR region[ad] OR judetul[ad] OR Raul[ad])) OR Prahova[ad] OR Salaj[ad] OR "Satu Mare"[ad] OR Sibiu[ad] OR Suceava[ad] OR Teleorman[ad] OR Timis[ad] OR Tulcea[ad] OR Valcea[ad] OR Vilcea[ad] OR Vaslui[ad] OR Vrancea[ad] OR Timisoara[ad] OR Temeswar[ad] OR Temeschburg[ad] OR Temeschwar[ad] OR Temesvar[ad] OR Temisvar[ad] OR Timisvar[ad] OR Temesva[ad] OR Craiova[ad] OR Ploiesti[ad] OR Ploesti[ad] OR Oradea[ad] OR Varad[ad] OR Varat[ad]) OR (Portugal[ad] OR Portugues*[ad] OR Azores[ad] OR Acores[ad] OR Madeira[ad] OR Alentejo[ad] OR Algarve[ad] OR Lisboa[ad] OR Lisbon[ad] OR "Alto Tras-os-Montes"[ad] OR (Ave[ad] AND (community[ad] OR intermunicipal[ad] OR comunidade[ad])) OR Mondego[ad] OR Vouga[ad] OR Beira[ad] OR Cavado[ad] OR Lafoes[ad] OR Douro[ad] OR Porto[ad] OR Oporto[ad] OR Tejo[ad] OR Minho[ad] OR Setubal[ad] OR Pinhal[ad] OR "Serra da Estrela"[ad] OR Tamega[ad] OR Leira[ad] OR Santarem[ad] OR Beja[ad] OR Faro[ad] OR Evora[ad] OR Portalegre[ad] OR "Castelo Branco"[ad] OR Guarda[ad] OR Cimbra[ad] OR Aveiro[ad] OR Viseu[ad] OR Braganca[ad] OR Braganza[ad] OR Braga[ad] OR "Vila real"[ad] OR "Viana do Castelo"[ad] OR Gaia[ad] OR Amadora[ad] OR Funchal[ad] OR Coimbra[ad] OR Almada[ad] OR (Agualva[ad] AND Cacem[ad])) OR (Poland[ad] OR Polska[ad] OR Polish[ad] OR Pole[ad] OR Poles[ad] OR Polski[ad] OR Polak[ad] OR Polka[ad] OR Polacy[ad] OR Dolnoslaskie[ad] OR Silesia*[ad] OR Slask[ad] OR Pomorskie[ad] OR Pomerania*[ad] OR Kujawsko[ad] OR Kuyavian[ad] OR Lodzkie[ad] OR Lodz[ad] OR Lubelskie[ad] OR Lublin[ad] OR Lubuskie[ad] OR Lubusz[ad] OR Lubus[ad] OR Malopolskie[ad] OR Mazowieckie[ad] OR Mazowske[ad] OR Masovia[ad] OR Masovian[ad] OR Opolskie[ad] OR Opole[ad] OR Podkarpackie[ad] OR Subcarpathian*[ad] OR Podlaskie[ad] OR Podlachia[ad] OR Podlasie[ad] OR Slaskie[ad] OR Swietokrzyskie[ad] OR "Varmia Mazuria"[ad] OR "Varmian Mazurian"[ad] OR "Varmia Masuria"[ad] OR "Varmian Masurian"[ad] OR "Warmia Mazury"[ad] OR "Warminsko Mazurskie"[ad] OR "Warmian Masurian"[ad] OR Wielkopolskie[ad] OR Zachodniopomorskie[ad] OR Warsaw[ad] OR Warszawa[ad] OR Krakow[ad] OR Cracow[ad] OR Wroclaw[ad] OR Poznan[ad] OR Gdansk[ad] OR Szczecin[ad] OR Bydgoszcz[ad] OR Katowice[ad]) OR (Netherlands[ad] OR Nederland*[ad] OR Dutch*[ad] OR Drenthe[ad] OR Flevoland[ad] OR Friesland[ad] OR Fryslan[ad] OR Frisia[ad] OR Gelderland[ad] OR Guelders[ad] OR Groningen[ad] OR Limburg[ad] OR Brabant[ad] OR Holland[ad] OR Overijssel[ad] OR Overissel[ad] OR Utrecht[ad] OR Zeeland[ad] OR Amsterdam[ad] OR Rotterdam[ad] OR Hague[ad] OR "s-Gravenhage"[ad] OR "Den Haag"[ad] OR Eindhoven[ad] OR Tilburg[ad] OR Almere[ad] OR Breda[ad] OR Nijmegen[ad] OR Nimeguen[ad]) OR (Malta[ad] OR Maltese*[ad] OR Maltin[ad] OR Gozo[ad] OR Ghawdex[ad] OR Valletta[ad] OR "Ill Belt"[ad] OR Birkirkara[ad] OR "B Kara"[ad] OR "B'Kara"[ad] OR Birchircara[ad] OR Mosta[ad] OR Qormi[ad] OR "St Paul s Bay"[ad] OR "St Paul's Bay"[ad] OR "Pawl il Bahar"[ad] OR Zabbar[ad] OR Sliema[ad] OR Naxxar[ad] OR Gwann[ad] OR "St John"[ad] OR Zebbug[ad] OR "Citta rohan"[ad] OR Fgura[ad]) OR (Luxembourg*[ad] OR Luxemburg[ad] OR Letzebuerg[ad] OR Diekirch[ad] OR Grevenmacher[ad] OR "Esch sur Alzette"[ad] OR "Esch Uelzecht"[ad] OR "Esch an der Alzette"[ad] OR "Esch an der Alzig"[ad] OR Dudelange[ad] OR Diddeleng[ad] OR Dudelingen[ad] OR Duedelingen[ad] OR Schifflange[ad] OR Scheffleng[ad] OR Schifflingen[ad] OR Bettembourg[ad] OR Beetebuerg[ad] OR Bettemburg[ad] OR Petange[ad] OR Peiteng[ad] OR Petingen[ad] OR Ettelbruck[ad] OR Ettelbreck[ad] OR Ettelbrueck[ad] OR Diekirch[ad] OR Dikrech[ad] OR Strassen[ad] OR Stroossen[ad] OR Bertrange[ad] OR Bartreng[ad] OR Bartringen[ad]) OR (Lithuania*[ad] OR "Lietuvos Respublika"[ad] OR Lietuva[ad] OR lietuviu[ad] OR Alytus[ad] OR Alytaus[ad] OR Kaunas[ad] OR Kauno[ad] OR Klaipeda[ad] OR Klaipedos[ad] OR Marijampoles[ad] OR Marijampole[ad] OR Panevezys[ad] OR Panevezio[ad] OR Siauliai[ad] OR Siauliu[ad] OR Taurages[ad] OR Taurage[ad] OR Telsiu[ad] OR Telsiai[ad] OR Utenos[ad] OR Utena[ad] OR Vilnius[ad] OR Vilniaus[ad] OR Mazeikiai[ad] OR Jonava[ad] OR Mazeikiu[ad] OR Jonavos[ad]) OR (Latvi*[ad] OR Riga[ad] OR Courland[ad] OR Kurzeme[ad] OR Kurland[ad] OR Latgale[ad] OR Lettgallia[ad] OR Latgola[ad] OR Latgalia[ad] OR Vidzeme[ad] OR Vidumo[ad] OR Semigallia[ad] OR Semigalia[ad] OR Zemgale[ad] OR Pieriga[ad] OR Daugavpils[ad] OR Dinaburg[ad] OR Jekabpils[ad] OR Jakobstadt[ad] OR Jelgava[ad] OR Jurmala[ad] OR Liepaja[ad] OR Libau[ad] OR Rezekne[ad] OR Rezne[ad] OR Rositten[ad] OR Valmiera[ad] OR Wolmar[ad] OR Ventspils[ad] OR Windau[ad] OR Ogre[ad]) OR (Italy[ad] OR Italia*[ad] OR Abruzzo[ad] OR Abruzzi[ad] OR Basilicata[ad] OR Lucania[ad] OR Calabria[ad] OR Campania[ad] OR "Emilia Romagna"[ad] OR "friuli venezia giulia"[ad] OR Lazio[ad] OR Latium[ad] OR Liguria*[ad] OR Lombardy[ad] OR Lombardia[ad] OR Marche[ad] OR Marches[ad] OR Molisano[ad] OR Molise[ad] OR Piedmont*[ad] OR Piemonte[ad] OR Bolzano[ad] OR Bozen[ad] OR Trentino[ad] OR Trento[ad] OR Puglia[ad] OR Apulia[ad] OR Sardinia[ad] OR Sardegna[ad] OR Sicily[ad] OR Sicilia[ad] OR Toscana[ad] OR Tuscany[ad] OR Umbria[ad] OR "Valle d Aosta"[ad] OR "Vallee d Aoste"[ad] OR "Valle d'Aosta"[ad] OR "Vallee d'Aoste"[ad] OR "Aosta Valley"[ad] OR Veneto[ad] OR Venetia[ad] OR Triveneto[ad] OR Rome[ad] OR Roma[ad] OR Milan[ad] OR Milano[ad] OR Naples[ad] OR Napoli[ad] OR Turin[ad] OR Torino[ad] OR Palermo[ad] OR Genoa[ad] OR Genova[ad] OR Bologna[ad] OR Florence[ad] OR Firenze[ad] OR Bari[ad] OR Catania[ad]) OR (Ireland[ad] OR Eire[ad] OR Irish*[ad] OR Fingal[ad] OR "Fine Gall"[ad] OR Dublin[ad] OR "Ath Cliath"[ad] OR "Dun Laoghaire"[ad] OR Wicklow[ad] OR "Cill Mhantain"[ad] OR "Chill Mhantain"[ad] OR Wexford[ad] OR "Loch Garman"[ad] OR Carlow[ad] OR Ceatharlach[ad] OR Kildare[ad] OR "Cill Dara"[ad] OR "Chill Dara"[ad] OR Meath[ad] OR "An Mhi"[ad] OR "Contae na Mi"[ad] OR Louth[ad] OR "Contae Lu"[ad] OR Monaghan[ad] OR Muineachan[ad] OR Mhuineachain[ad] OR Cavan[ad] OR "An Cabhan"[ad] OR "An Cabhain"[ad] OR Longford[ad] OR "An Longfort"[ad] OR "an Longfoirt"[ad] OR Langfurd[ad] OR Westmeath[ad] OR "An Iarmhi"[ad] OR "na Iarmhi"[ad] OR Offaly[ad] OR "Uibh Fhaili"[ad] OR Laois[ad] OR Laoise[ad] OR Kilkenny[ad] OR "Chill Chainnigh"[ad] OR "Cill Chainnigh"[ad] OR Waterford[ad] OR "Port Lairge"[ad] OR Watterford[ad] OR Cork[ad] OR Corcaigh[ad] OR Chorcai[ad] OR Kerry[ad] OR Ciarrai[ad] OR Chiarrai[ad] OR Limerick[ad] OR Luimneach[ad] OR Luimnigh[ad] OR Tipperary[ad] OR "Tiobraid Arann"[ad] OR "Thiobraid Arann"[ad] OR Clare[ad] OR "An Clar"[ad] OR "an Chlair"[ad] OR Galway[ad] OR Gaillimh[ad] OR "na Gaillimhe"[ad] OR Mayo[ad] OR "Maigh Eo"[ad] OR "Mhaigh Eo"[ad] OR Roscommon[ad] OR "Ros comain"[ad] OR Sligo[ad] OR Sligeach[ad] OR Shligigh[ad] OR Leitrim[ad] OR Liatroim[ad] OR Liatroma[ad] OR Donegal[ad] OR "Dhun na nGall"[ad] OR Dinnygal[ad] OR Dunnyga[ad] OR Leinster[ad] OR Laighin[ad] OR "Cuige Laighean"[ad] OR Munster[ad] OR Mumhain[ad] OR "Cuige Mumhan"[ad] OR Connacht[ad] OR Connachta[ad] OR Drogheda[ad] OR "Droichead Atha"[ad] OR Dundalk[ad] OR "Dun Dealgan"[ad] OR Swords[ad] OR Sord[ad] OR Bray[ad] OR Bre[ad] OR Navan[ad] OR "An Uaimh"[ad]) OR (Hungar*[ad] OR Magyarorszag[ad] OR Magyar*[ad] OR Dunantuli[ad] OR Transdanubia[ad] OR Dunantul[ad] OR "Great Plain"[ad] OR "Eszak Alfold"[ad] OR "Del Alfold"[ad] OR "Alfold es eszak"[ad] OR "Northern Alfold"[ad] OR "North Alfold"[ad] OR "South Alfold"[ad] OR "Southern Alfold"[ad] OR Bacs[ad] OR Kiskun[ad] OR Baranya[ad] OR Bekes[ad] OR Borsod[ad] OR Abauj[ad] OR Zemplen[ad] OR Budapest[ad] OR Csongrad[ad] OR Fejer[ad] OR gyor[ad] OR moson[ad] OR sopron[ad] OR hajdu[ad] OR bihar[ad] OR Heves[ad] OR "jasz nagykun szolnok"[ad] OR komarom[ad] OR esztergom[ad] OR Nograd[ad] OR (Pest[ad] AND (megye[ad] OR county[ad])) OR Somogy[ad] OR szabolcs[ad] OR szatmar[ad] OR bereg[ad] OR Tolna[ad] OR Vas[ad] OR Veszprem[ad] OR Zala[ad] OR Debrecen[ad] OR Miskolc[ad] OR Szeged[ad] OR Pecs[ad] OR Gyor[ad] OR Nyiregyhaza[ad] OR Kecskemet[ad] OR Szekesfehervar[ad] OR Szombathely[ad]) OR (Greece[ad] OR "Hellenic republic"[ad] OR Greek*[ad] OR Ellada[ad] OR Elladas[ad] OR "Elliniki Dimokratia"[ad] OR Hellas[ad] OR Hellenes[ad] OR Attica[ad] OR Attiki[ad] OR Makedonia*[ad] OR Macedonia[ad] OR Thraki[ad] OR Thrace[ad] OR Crete[ad] OR Kriti[ad] OR "Ionia Nisia"[ad] OR "Ionion neson"[ad] OR "Ionion nIson"[ad] OR "Ionian islands"[ad] OR "Ionian island"[ad] OR Epirus[ad] OR Ipeiros[ad] OR "Perifereia Ipeirou"[ad] OR "North aegean"[ad] OR "Northern Aegean"[ad] OR "Aegean islands"[ad] OR "Aegean island"[ad] OR "Nisoi Agaiou"[ad] OR "Notio Aigaio"[ad] OR Peloponnese[ad] OR Peloponniso*[ad] OR Thessaly[ad] OR Thessalia[ad] OR Thessalian[ad] OR Petthalia[ad] OR "Voreio Aigaio"[ad] OR "Voreio Aigaiou"[ad] OR "South aegean"[ad] OR "Southern Aegean"[ad] OR "Mount athos"[ad] OR "Oros Athos"[ad] OR Cyclades[ad] OR Cycklades[ad] OR Kiklades[ad] OR Dodecanese[ad] OR Dodekanisa[ad] OR Athens[ad] OR Athina[ad] OR Thessaloniki[ad] OR Thessalonica[ad] OR Patras[ad] OR Patra[ad] OR Pireas[ad] OR Piraeus[ad] OR Larissa[ad] OR Larisa[ad] OR Heraklion[ad] OR Heraclion[ad] OR Iraklion[ad] OR Irakleion[ad] OR Iraklio[ad] OR Volos[ad] OR Rhodes[ad] OR Rodos[ad] OR Ioannina[ad] OR Janina[ad] OR Yannena[ad] OR Chania[ad] OR Chalcis[ad] OR Chalkida[ad]) OR (German*[ad] OR Deutsch*[ad] OR Bundesrepublik[ad] OR Westdeutschland[ad] OR Ostdeutschland[ad] OR Baden[ad] OR Wuerttemberg[ad] OR Wurttemberg[ad] OR Bayern[ad] OR Bavaria[ad] OR Berlin[ad] OR Brandenburg[ad] OR Bremen[ad] OR Oldenburg[ad] OR Mitteldeutschland[ad] OR Rhein[ad] OR Rhine[ad] OR Hannover[ad] OR Braunschweig[ad] OR Gottingen[ad] OR Goettingen[ad] OR Nurnberg[ad] OR Nuernberg[ad] OR Ruhr[ad] OR Koln[ad] OR koeln[ad] OR Bonn[ad] OR Hamburg[ad] OR Hessen[ad] OR Hesse[ad] OR Hessia[ad] OR Mecklenburg[ad] OR Vorpommern[ad] OR Pomerania[ad] OR Niedersachsen[ad] OR Neddersassen[ad] OR Saxony[ad] OR Niederbayern[ad] OR "Northern Rhine"[ad] OR "North Rhine"[ad] OR Westphalia[ad] OR Westfalen[ad] OR "Rhineland Palatinate"[ad] OR "Rheinland Pfalz"[ad] OR Saarland[ad] OR Sachsen[ad] OR "Schleswig Holstein"[ad] OR Thuringia[ad] OR Thuringen[ad] OR Thueringen[ad] OR Munchen[ad] OR Muenchen[ad] OR Munich[ad] OR Frankfurt[ad] OR Stuttgart[ad] OR Dusseldorf[ad] OR Duesseldorf[ad] OR Dortmund[ad] OR Essen[ad]) OR (France[ad] OR French*[ad] OR Francais*[ad] OR Alsace[ad] OR Elsass[ad] OR Aquitaine[ad] OR Aquitania[ad] OR Akitania[ad] OR Aguiene[ad] OR Auvergne[ad] OR Auvernhe[ad] OR Auvernha[ad] OR Normandie[ad] OR Normandy[ad] OR Normaundie[ad] OR Bourgogne[ad] OR Burgundy[ad] OR Bregogne[ad] OR Borgoegne[ad] OR Borgogne[ad] OR Brittany[ad] OR Breizh[ad] OR Bertaeyn[ad] OR Bretagne[ad] OR "Champagne Ardenne"[ad] OR Corse[ad] OR Corsica[ad] OR "Franche Comte"[ad] OR "Frantche Comte"[ad] OR "Franche Comtat"[ad] OR Guadeloupe[ad] OR Guyane[ad] OR Guiana[ad] OR "Languedoc Roussillon"[ad] OR "Lengadoc Rosselhon"[ad] OR "Llenguadoc-Rossello"[ad] OR Limousin[ad] OR Lemosin[ad] OR Lorraine[ad] OR Lothringen[ad] OR Lottringe[ad] OR Martinique[ad] OR "Midi Pyrenees"[ad] OR "Miegjorn Pireneus"[ad] OR "Mieidia Pireneus"[ad] OR "Mediodia Pirineos"[ad] OR "Pays de la Loire"[ad] OR "Broiou al Liger"[ad] OR Picardie[ad] OR Picardy[ad] OR "Poitou Charentes"[ad] OR "Peitau Charantas"[ad] OR "Poetou-Cherentes"[ad] OR Provence[ad] OR Provenca[ad] OR Prouvenco[ad] OR "Cote d Azur"[ad] OR "Cote d'Azur"[ad] OR "Costo d'Azur"[ad] OR "Costo d Azur"[ad] OR "Costa d'Azur"[ad] OR "Costa d Azur"[ad] OR Reunion[ad] OR "Rhone Alpes"[ad] OR "Rono Arpes"[ad] OR "Rose Aups"[ad] OR Ain[ad] OR Aisne[ad] OR Allier[ad] OR "Alpes de Haute Provence"[ad] OR "Haute Alpes"[ad] OR "Alpes Maritimes"[ad] OR Ardeche[ad] OR Ardennes[ad] OR Ariege[ad] OR Aube[ad] OR Aude[ad] OR Aveyron[ad] OR "Bas Rhin"[ad] OR "Bouches du Rhone"[ad] OR Calvados[ad] OR Cantal[ad] OR Charente[ad] OR Cher[ad] OR Correze[ad] OR "Corse du Sud"[ad] OR "Cote d Or"[ad] OR "Cote d'Or"[ad] OR "Cotes d Armor"[ad] OR "Cotes d'Armor"[ad] OR Creuse[ad] OR "Deux Sevres"[ad] OR Dordogne[ad] OR Doubs[ad] OR Drome[ad] OR Essonne[ad] OR Eure[ad] OR Finistere[ad] OR Gard[ad] OR Gers[ad] OR Gironde[ad] OR "Haute Corse"[ad] OR "Haute Garonne"[ad] OR "Haute Marne"[ad] OR "Hautes Alpes"[ad] OR "Haute Saone"[ad] OR "Haute Savoie"[ad] OR "Hautes Pyrenees"[ad] OR "Haute Vienne"[ad] OR "Haut Rhin"[ad] OR "Hauts de Seine"[ad] OR Herault[ad] OR "Ile de France"[ad] OR "Ille et Vilaine"[ad] OR Indre[ad] OR Isere[ad] OR Jura[ad] OR Landes[ad] OR Loire[ad] OR Loiret[ad] OR (Lot[ad] AND (departement[ad] OR department[ad])) OR "Lot et Garonne"[ad] OR "Loir et Cher"[ad] OR Lozere[ad] OR Manche[ad] OR Marne[ad] OR Mayenne[ad] OR Mayotte[ad] OR "Meurthe et Moselle"[ad] OR Meuse[ad] OR Morbihan[ad] OR Moselle[ad] OR (Nord[ad] AND (department[ad] OR departement[ad])) OR Nievre[ad] OR Oise[ad] OR Orne[ad] OR "Pas de calais"[ad] OR "Noord-Nauw van Kales"[ad] OR Paris[ad] OR "Puy de dome"[ad] OR "Pyrenees Atlantiques"[ad] OR "Pyrenees Orientales"[ad] OR Rhone[ad] OR Sarthe[ad] OR Savoie[ad] OR "Seine et Marne"[ad] OR "Seine Maritime"[ad] OR Somme[ad] OR Tarn[ad] OR "Territoire de Belfort"[ad] OR "Val de Marne"[ad] OR "Val d Oise"[ad] OR Var[ad] OR Vaucluse[ad] OR Vendee[ad] OR Vienne[ad] OR Vosges[ad] OR Yonne[ad] OR Yvelines[ad] OR Marseille[ad] OR Lyon[ad] OR Nice[ad] OR Nantes[ad] OR Strasbourg[ad] OR Montpellier[ad] OR Bordeaux[ad] OR Lille[ad] OR Toulouse[ad] OR "Outre Mer"[ad] OR "Seine Saint Denis"[ad]) OR (Finland[ad] OR Finnish*[ad] OR Finn[ad] OR Finns[ad] OR Suomi[ad] AND Suomen[ad] OR Suomalaiset[ad] OR Aland[ad] OR Ahvenanmaa[ad] OR Uusimaa[ad] OR Nyland[ad] OR Karelia[ad] OR Karjala[ad] OR Karelen[ad] OR Ostrobothnia[ad] OR Pohjanmaa[ad] OR Osterbotten[ad] OR Savonia[ad] OR Savo[ad] OR Savolax[ad] OR Kainuu[ad] OR Kajanaland*[ad] OR "Kanta Hame"[ad] OR Tavastia[ad] OR Tavastland[ad] OR Kymenlaakso[ad] OR Kymmenedalen[ad] OR Lapland[ad] OR Lappi[ad] OR Lappland[ad] OR "Paijat Hame"[ad] OR Pirkanmaa[ad] OR Birkaland[ad] OR Satakunta[ad] OR Satakunda[ad] OR Helsinki[ad] OR Helsingfors[ad] OR Espoo[ad] OR Esbo[ad] OR Tampere[ad] OR Tammerfors[ad] OR Vantaa[ad] OR Vanda[ad] OR Oulu[ad] OR Uleaborg[ad] OR Turku[ad] OR Abo[ad] OR Jyvaskyla[ad] OR Kuopio[ad] OR Lahti[ad] OR Lahtis[ad] OR Kouvola[ad]) OR (Estonia*[ad] OR Eesti[ad] OR Eestlased[ad] OR Eestlane[ad] OR Harju[ad] OR Harjumaa[ad] OR Hiiu[ad] OR Hiiumaa[ad] OR "Ida Viru"[ad] OR "Ida Virumaa"[ad] OR Jarvamaa[ad] OR Jarva[ad] OR Jogevamaa[ad] OR Jogeva[ad] OR Laanemaa[ad] OR Laane[ad] OR "Laane Virumaa"[ad] OR Parnu[ad] OR Parnumaa[ad] OR Polva[ad] OR Polvamaa[ad] OR Rapla[ad] OR Raplamaa[ad] OR Saare[ad] OR Saaremaa[ad] OR Tartu[ad] OR Tartumaa[ad] OR Valga[ad] OR Valgamaa[ad] OR Viljandimaa[ad] OR Viljandi[ad] OR Voru[ad] OR Vorumaa[ad] OR Tallinn[ad] OR Narva[ad] OR "Kohtla Jarve"[ad] OR Rakvere[ad] OR Maardu[ad] OR Sillamae[ad] OR Kuressaare[ad]) OR (Denmark[ad] OR Danish*[ad] OR dane[ad] OR danes[ad] OR Danmark[ad] OR dansk*[ad] OR Hovedstaden[ad] OR Midtjylland[ad] OR Nordjylland[ad] OR Sjaelland[ad] OR Sealand[ad] OR "Zealand region"[ad] OR "region Zealand"[ad] OR Syddanmark[ad] OR Jutland[ad] OR Jylland[ad] OR Sonderjyllands[ad] OR Copenhagen[ad] OR Kobenhavn[ad] OR Arhus[ad] OR Aarhus[ad] OR Bornholm[ad] OR Frederiksberg[ad] OR Frederiksborg[ad] OR Ringkjobing[ad] OR Viborg[ad] OR Vejle[ad] OR Roskilde[ad] OR Storstrom[ad] OR Vestsjaellands[ad] OR "West Zealand"[ad] OR Funen[ad] OR Ribe[ad] OR "Kalaallit Nunaat"[ad] OR Gronland[ad] OR Foroyar[ad] OR Faeroerne[ad] OR "Faroe islands"[ad] OR Aalborg[ad] OR Alborg[ad] OR Odense[ad] OR Esbjerg[ad] OR Gentofte[ad] OR Gladsaxe[ad] OR Randers[ad] OR Kolding[ad]) OR (Czech*[ad] OR Cesky[ad] OR Ceska[ad] OR Cech[ad] OR Cestina[ad] OR Prague[ad] OR Praha[ad] OR Prag[ad] OR Stredoces*[ad] OR Jihoces*[ad] OR Bohemia[ad] OR Bohemian[ad] OR Plzen*[ad] OR Pilsen[ad] OR Karlovars*[ad] OR "Karlovy Vary"[ad] OR Usteck*[ad] OR Usti[ad] OR Liberec*[ad] OR "Hradec Kralove"[ad] OR Kralovehradec*[ad] OR Pardubic*[ad] OR Olomouc*[ad] OR Olomoc[ad] OR Holomoc[ad] OR Moravskoslezs*[ad] OR Jihomorav*[ad] OR Moravia[ad] OR Moravian[ad] OR Morava[ad] OR Vysocina[ad] OR Zlin[ad] OR Zlinsk*[ad] OR "Ceske Budejovice"[ad] OR Budweis[ad] OR Brno[ad] OR Ostrava[ad]) OR (Cyprus[ad] OR Cypriot*[ad] OR Kypros[ad] OR Kibris*[ad] OR kypriaki[ad] OR Kyprioi[ad] OR Nicosia[ad] OR Lefkosa[ad] OR Lefkosia[ad] OR Famagusta[ad] OR Magusa[ad] OR Ammochostos[ad] OR Gazimagusa[ad] OR Kyrenia[ad] OR Girne[ad] OR Keryneia[ad] OR Larnaca[ad] OR Larnaka[ad] OR Iskele[ad] OR Limassol[ad] OR Lemesos[ad] OR Limasol[ad] OR Leymosun[ad] OR Paphos[ad] OR Pafos[ad] OR Baf[ad] OR Strovolos[ad] OR Lakatamia[ad] OR Lakadamya[ad] OR "Kato Polemidia"[ad] OR "Kato Polemidhia"[ad] OR Aglandjia[ad] OR Eglence[ad] OR Aglantzia[ad] OR Aradhippou[ad] OR Aradippou[ad] OR Engomi[ad]) OR (Croat*[ad] OR Hrvatsk*[ad] OR hrvati[ad] OR Bjelovar[ad] OR "Bjelovarsko bilogorska"[ad] OR "Brod Posavina"[ad] OR "Brodsko posavska"[ad] OR "Dubrovnik Neretva"[ad] OR "dubrovacko neretvanska"[ad] OR Zagreb[ad] OR Zagrebacka[ad] OR Istria[ad] OR Istarska[ad] OR Karlovacka[ad] OR Karlovac[ad] OR "Koprivnicko krizevacka"[ad] OR Koprivnica[ad] OR Krizevci[ad] OR "Krapina Zagorje"[ad] OR "Krapinsko zagorska"[ad] OR "Lika Senj"[ad] OR "Licko senjska"[ad] OR Medimurska[ad] OR Medimurje[ad] OR Osijek[ad] OR Baranja[ad] OR "Osjecko baranjska"[ad] OR "Pozega Slavonia"[ad] OR "Pozesko slavonska"[ad] OR "Primorje Gorski Kotar"[ad] OR "Primorsko goranska"[ad] OR "Sibensko kninska"[ad] OR "Sibensko kninske"[ad] OR Sibenik[ad] OR Knin[ad] OR Sisak[ad] OR "Sisacko moslavacka"[ad] OR Moslavina[ad] OR "Splitsko dalmatinska"[ad] OR Split[ad] OR Dalmatia[ad] OR Varazdin[ad] OR Varazdinska[ad] OR Viroviticko[ad] OR podravska[ad] OR Virovitica[ad] OR Podravina[ad] OR "Vukovarsko srijemska"[ad] OR Vukovar[ad] OR Srijem[ad] OR Zadar[ad] OR Zadarska[ad] OR Rijeka[ad] OR "Velika gorica"[ad] OR "Slavonski brod"[ad] OR Pula[ad]) OR (Bulgaria*[ad] OR Balgariya[ad] OR Balgarija[ad] OR Blagoevgrad*[ad] OR "Pirin Macedonia"[ad] OR Burgas[ad] OR Dobrich[ad] OR Gabrovo[ad] OR Haskovo[ad] OR Kardzhali[ad] OR Kurdzhali[ad] OR Kyustendil[ad] OR Lovech[ad] OR Lovec[ad] OR Montana[ad] OR Pazardzhik[ad] OR Pernik[ad] OR Pleven*[ad] OR Plovdiv[ad] OR Razgrad[ad] OR Rousse[ad] OR Ruse[ad] OR Rusenka[ad] OR Shumen[ad] OR Silistra[ad] OR Sliven[ad] OR Smolyan[ad] OR Sofia[ad] OR Sofyiska[ad] OR Sofiiska[ad] OR "Stara Zagora"[ad] OR Targovishte[ad] OR Varna[ad] OR "Veliko Tarnovo"[ad] OR Vidin[ad] OR Vratsa[ad] OR Vratza[ad] OR Yambol[ad]) OR (Belgi*[ad] OR Belge*[ad] OR Belg[ad] OR Brussel*[ad] OR Bruxelles[ad] OR Bruxelloise[ad] OR Walloon*[ad] OR Wallon*[ad] OR Vlaams[ad] OR Flander*[ad] OR Flandern[ad] OR Flandre[ad] OR Flemish[ad] OR Flamand[ad] OR Flemisch[ad] OR Flamisch*[ad] OR Vlaanderen[ad] OR Flamande[ad] OR Waals[ad] OR Antwerp*[ad] OR Anvers[ad] OR Henegouwen[ad] OR Hennegau[ad] OR Hainault[ad] OR Hainaut[ad] OR Liege[ad] OR Luik[ad] OR Luttich[ad] OR Limbourg[ad] OR Limburg[ad] OR Namur[ad] OR Namen[ad] OR Ostflandern[ad] OR Westflandern[ad] OR Ghent[ad] OR Gent[ad] OR Gand[ad] OR Charleroi[ad] OR Bruges[ad] OR Brugge*[ad] OR Schaerbeek[ad] OR Schaarbeek[ad] OR Anderlecht[ad] OR Leuven[ad] OR Louvain[ad]) OR (Austria*[ad] OR Osterreich*[ad] OR Oesterreich*[ad] OR Ostosterreich[ad] OR Ostoesterreich[ad] OR Sudosterreich[ad] OR Sudoesterreich[ad] OR Westosterreich[ad] OR Westoesterreich[ad] OR Burgenland[ad] OR Carinthia[ad] OR Karnten[ad] OR Kaernten[ad] OR Niederosterreich[ad] OR Niederoesterreich[ad] OR Oberosterreich[ad] OR Oberoesterreich[ad] OR Salzburg[ad] OR Saizburg[ad] OR Styria[ad] OR Steiermark[ad] OR Tyrol[ad] OR Tirol[ad] OR Vorarlberg[ad] OR Vienna[ad] OR Wien[ad] OR Graz[ad] OR Linz[ad] OR Innsbruck[ad] OR Klagenfurt[ad] OR Villach[ad] OR Wels[ad] OR "St Polten"[ad] OR "St Poelten"[ad] OR "Sankt Polten"[ad] OR "Sankt Poelten"[ad] OR Dornbirn[ad]))

### Embase search strings

#### #a string for Hepatitis B and Hepatitis C virus

'hepatitis B'/exp OR 'hepatitis B antibody'/exp OR 'hepatitis B antigen'/exp OR 'hepatitis b':ti,ab OR ‘hbv’:ti OR 'hep b':ti,ab OR ‘hbsag’:ti,ab OR 'hbs ag':ti,ab OR 'hepatitis C'/exp OR 'Hepacivirus'/exp OR 'hepatitis C antibody'/exp OR 'hepatitis C antigen'/exp OR 'hepatitis c':ti,ab OR hepaciviru*:ti,ab OR ‘hcv’:ti OR 'hep c':ti,ab OR 'blood borne virus':ti,ab OR 'blood borne viruses':ti,ab OR ‘bbv’:ti

#### #b string for testing

'mass screening'/exp OR 'mandatory testing'/exp OR 'point of care testing'/exp OR 'dried blood spot testing'/exp OR 'early diagnosis'/de OR ‘mandatory test’:ti,ab OR ‘mandatory testing’:ti,ab OR ‘point of care’:ti,ab OR ‘dried blood spot’:ti,ab OR screen:ti,ab OR screened:ti,ab OR screening:ti,ab OR (‘hepatitis B’ NEAR/2 test*):ti,ab OR (‘hepatitis C’ NEAR/2 test*):ti,ab OR (hbv NEAR/2 test*):ti,ab OR (hcv NEAR/2 test*):ti,ab OR ‘bedside test’:ti,ab OR ‘bedside testing’:ti,ab OR diagnos*:ti,ab OR (case* NEAR/2 find*):ti,ab OR (case* NEAR/2 identif*):ti,ab OR (case* NEAR/2 detect*):ti,ab

#### #c string for linkage to care

((uptake OR adhere* OR comply* OR compliance OR complied OR retain* OR retention OR link* OR initiat* OR begin* OR began OR start* OR enter* OR commence* OR refer*) NEAR/4 (care OR healthcare OR treat* OR therap*)):ti,ab OR prevent:ti,ab OR prevention:ti,ab OR preventative:ti,ab

#### #d string for intervention

'program evaluation'/exp OR 'health promotion'/exp OR 'feasibility study'/exp OR 'pilot study'/exp OR intervention*:ti,ab OR approach*:ti,ab OR program*:ti,ab OR campaign*:ti,ab OR promot*:ti,ab OR pilot*:ti,ab OR evaluation*:ti,ab OR appraisal*:ti,ab OR assessment*:ti,ab OR feasibility:ti,ab OR service*:ti,ab OR strateg*:ti,ab OR outreach:ti,ab OR scheme*:ti,ab OR project*:ti,ab OR policy:ti,ab OR policies:ti,ab OR audit*:ti,ab OR (community NEAR/3 based):ti,ab

#### #d string for EU/EEA

'european union'/exp OR 'europe'/de OR europe*:ab,ti OR europa*:ab,ti OR eu:ab,ti OR eea:ab,ti OR efta:ab,ti OR 'eu/eea':ab,ti OR 'eu/efta':ab,ti OR ecsc:ab,ti OR euratom:ab,ti OR eurozone:ab,ti OR eec:ab,ti OR ec:ab,ti OR (schengen:ab,ti AND (area:ab,ti OR countr*:ab,ti OR region*:ab,ti OR state:ab,ti OR states:ab,ti)) OR euroregion:ab,ti OR euroregions:ab,ti OR 'eastern europe'/de OR 'western europe'/de OR 'balkan peninsula'/exp OR balkan:ab,ti OR balkans:ab,ti OR 'baltic states'/de OR baltic:ab,ti OR 'southern europe'/de OR (mediterranean:ab,ti AND (area:ab,ti OR countr*:ab,ti OR region*:ab,ti OR state:ab,ti OR states:ab,ti)) OR (alpine:ab,ti AND (area:ab,ti OR countr*:ab,ti OR region*:ab,ti OR state:ab,ti OR states:ab,ti)) OR 'scandinavia'/de OR scandinavia:ab,ti OR scandinavian:ab,ti OR (nordic NEXT/1 (countr* OR state*)):ab,ti OR danubian:ab,ti OR 'iberian peninsula':ab,ti OR 'peninsula iberica':ab,ti OR 'péninsule ibérique':ab,ti OR 'iberiar penintsula':ab,ti OR iberia:ab,ti OR anatolia:ab,ti OR anadolu:ab,ti OR anatole:ab,ti OR anatolian:ab,ti OR 'yugoslavia (pre-1992)'/de OR 'yugoslavia'/exp OR yugoslavia:ab,ti OR 'czechoslovakia'/de OR czechoslovakia:ab,ti OR 'czecho slovakia':ab,ti OR ceskoslovensko:ab,ti OR 'cesko slovensko':ab,ti OR 'benelux'/exp OR benelux:ab,ti OR fennoscandia:ab,ti OR 'fenno scandinavia':ab,ti OR fennoskandi*:ab,ti OR (visegrád:ab,ti AND (group:ab,ti OR four:ab,ti OR triangle:ab,ti)) OR 'visegrádská čtyřka':ab,ti OR 'visegrádská skupina':ab,ti OR 'visegrádi együttműködés':ab,ti OR 'visegrádi négyek':ab,ti OR 'grupa wyszehradzka':ab,ti OR 'vyšehradská skupina':ab,ti OR 'vyšehradská štvorka':ab,ti OR 'European'/de OR 'EU citizen'/de OR 'Central European'/de OR 'Eastern European'/de OR 'Northern European'/de OR 'Southern European'/de OR 'Western European'/de OR 'austrian'/exp OR 'austria'/exp OR austria*:ab,ti OR osterreich*:ab,ti OR oesterreich*:ab,ti OR ostosterreich:ab,ti OR ostoesterreich:ab,ti OR sudosterreich:ab,ti OR sudoesterreich:ab,ti OR westosterreich:ab,ti OR westoesterreich:ab,ti OR burgenland:ab,ti OR carinthia:ab,ti OR karnten:ab,ti OR kaernten:ab,ti OR niederosterreich:ab,ti OR niederoesterreich:ab,ti OR oberosterreich:ab,ti OR oberoesterreich:ab,ti OR salzburg:ab,ti OR saizburg:ab,ti OR styria:ab,ti OR steiermark:ab,ti OR tyrol:ab,ti OR tirol:ab,ti OR vorarlberg:ab,ti OR vienna:ab,ti OR wien:ab,ti OR graz:ab,ti OR linz:ab,ti OR innsbruck:ab,ti OR klagenfurt:ab,ti OR villach:ab,ti OR wels:ab,ti OR 'st polten':ab,ti OR 'st poelten':ab,ti OR 'sankt polten':ab,ti OR 'sankt poelten':ab,ti OR dornbirn:ab,ti OR 'Belgium'/exp OR 'Belgian'/exp OR Belgi*:ti,ab OR Belge*:ti,ab OR Belg:ti,ab OR Brussel*:ti,ab OR Bruxelles:ti,ab OR Bruxelloise:ti,ab OR Walloon*:ti,ab OR Wallon*:ti,ab OR Vlaams:ti,ab OR Flander*:ti,ab OR Flandern:ti,ab OR Flandre:ti,ab OR Flemish:ti,ab OR Flamand:ti,ab OR Flemisch:ti,ab OR Flämisch*:ti,ab OR Vlaanderen:ti,ab OR Flamande:ti,ab OR Waals:ti,ab OR Antwerp*:ti,ab OR Anvers:ti,ab OR Henegouwen:ti,ab OR Hennegau:ti,ab OR Hainault:ti,ab OR Hainaut:ti,ab OR Liege:ti,ab OR Luik:ti,ab OR Luttich:ti,ab OR Limbourg:ti,ab OR Limburg:ti,ab OR Namur:ti,ab OR Namen:ti,ab OR Ostflandern:ti,ab OR Westflandern:ti,ab OR Ghent:ti,ab OR Gent:ti,ab OR Gand:ti,ab OR Charleroi:ti,ab OR Bruges:ti,ab OR Brugge*:ti,ab OR Schaerbeek:ti,ab OR Schaarbeek:ti,ab OR Anderlecht:ti,ab OR Leuven:ti,ab OR Louvain:ti,ab OR 'bulgaria'/exp OR 'bulgarian (citizen)'/exp OR 'Bulgarian (people)'/exp OR bulgaria*:ti,ab OR balgariya:ab,ti OR balgarija:ab,ti OR blagoevgrad*:ab,ti OR 'croatia'/exp OR 'croatian (citizen)'/exp OR 'Croat (people)'/exp OR croat*:ab,ti OR hrvatsk*:ab,ti OR hrvati:ab,ti OR bjelovar:ab,ti OR 'bjelovarsko bilogorska':ab,ti OR 'brod posavina':ab,ti OR 'brodsko posavska':ab,ti OR 'dubrovnik neretva':ab,ti OR 'dubrovacko neretvanska':ab,ti OR zagreb:ab,ti OR zagrebacka:ab,ti OR istria:ab,ti OR istarska:ab,ti OR karlovacka:ab,ti OR karlovac:ab,ti OR 'koprivnicko krizevacka':ab,ti OR koprivnica:ab,ti OR krizevci:ab,ti OR 'krapina zagorje':ab,ti OR 'krapinsko zagorska':ab,ti OR 'lika senj':ab,ti OR 'licko senjska':ab,ti OR medimurska:ab,ti OR medimurje:ab,ti OR osijek:ab,ti OR baranja:ab,ti OR 'osjecko baranjska':ab,ti OR 'pozega slavonia':ab,ti OR 'pozesko slavonska':ab,ti OR 'primorje gorski kotar':ab,ti OR 'primorsko goranska':ab,ti OR 'sibensko kninska':ab,ti OR 'sibensko kninske':ab,ti OR sibenik:ab,ti OR knin:ab,ti OR sisak:ab,ti OR 'sisacko moslavacka':ab,ti OR moslavina:ab,ti OR 'splitsko dalmatinska':ab,ti OR split:ab,ti OR dalmatia:ab,ti OR varazdin:ab,ti OR varazdinska:ab,ti OR viroviticko:ab,ti OR podravska:ab,ti OR virovitica:ab,ti OR podravina:ab,ti OR 'vukovarsko srijemska':ab,ti OR vukovar:ab,ti OR srijem:ab,ti OR zadar:ab,ti OR zadarska:ab,ti OR rijeka:ab,ti OR 'velika gorica':ab,ti OR 'slavonski brod':ab,ti OR pula:ab,ti OR 'cyprus'/exp OR 'cypriot'/exp OR cyprus:ab,ti OR cypriot*:ab,ti OR kypros:ab,ti OR kibris*:ab,ti OR kypriaki:ab,ti OR kyprioi:ab,ti OR nicosia:ab,ti OR lefkosa:ab,ti OR lefkosia:ab,ti OR famagusta:ab,ti OR magusa:ab,ti OR ammochostos:ab,ti OR gazimagusa:ab,ti OR kyrenia:ab,ti OR girne:ab,ti OR keryneia:ab,ti OR larnaca:ab,ti OR larnaka:ab,ti OR iskele:ab,ti OR limassol:ab,ti OR lemesos:ab,ti OR limasol:ab,ti OR leymosun:ab,ti OR paphos:ab,ti OR pafos:ab,ti OR baf:ab,ti OR strovolos:ab,ti OR lakatamia:ab,ti OR lakadamya:ab,ti OR 'kato polemidia':ab,ti OR 'kato polemidhia':ab,ti OR aglandjia:ab,ti OR eglence:ab,ti OR aglantzia:ab,ti OR aradhippou:ab,ti OR aradippou:ab,ti OR engomi:ab,ti OR 'czech (citizen)'/exp OR 'czech republic'/exp OR 'Czech (people)'/exp OR czech*:ab,ti OR cesky:ab,ti OR ceska:ab,ti OR cech:ab,ti OR cestina:ab,ti OR prague:ab,ti OR praha:ab,ti OR prag:ab,ti OR stredoces*:ab,ti OR jihoces*:ab,ti OR bohemia:ab,ti OR bohemian:ab,ti OR plzen*:ab,ti OR pilsen:ab,ti OR karlovars*:ab,ti OR 'karlovy vary':ab,ti OR usteck*:ab,ti OR usti:ab,ti OR liberec*:ab,ti OR 'hradec kralove':ab,ti OR kralovehradec*:ab,ti OR pardubic*:ab,ti OR olomouc*:ab,ti OR olomoc:ab,ti OR holomoc:ab,ti OR moravskoslezs*:ab,ti OR jihomorav*:ab,ti OR moravia:ab,ti OR moravian:ab,ti OR morava:ab,ti OR vysocina:ab,ti OR zlin:ab,ti OR zlinsk*:ab,ti OR 'ceske budejovice':ab,ti OR budweis:ab,ti OR brno:ab,ti OR ostrava:ab,ti OR 'Denmark'/exp OR 'Danish citizen'/exp OR 'Dane (people)'/exp OR Denmark:ti,ab OR Danish*:ti,ab OR dane:ti,ab OR danes:ti,ab OR Danmark:ti,ab OR dansk*:ti,ab OR Hovedstaden:ti,ab OR Midtjylland:ti,ab OR Nordjylland:ti,ab OR Sjaelland:ti,ab OR Sealand:ti,ab OR 'Zealand region':ti,ab OR 'region Zealand':ti,ab OR Syddanmark:ti,ab OR Jutland:ti,ab OR Jylland:ti,ab OR Sonderjyllands:ti,ab OR Copenhagen:ti,ab OR Kobenhavn:ti,ab OR Arhus:ti,ab OR Aarhus:ti,ab OR Bornholm:ti,ab OR Frederiksberg:ti,ab OR Frederiksborg:ti,ab OR Ringkjobing:ti,ab OR Viborg:ti,ab OR Vejle:ti,ab OR Roskilde:ti,ab OR Storstrøm:ti,ab OR Vestsjaellands:ti,ab OR 'West Zealand':ti,ab OR Funen:ti,ab OR Ribe:ti,ab OR 'Kalaallit Nunaat':ti,ab OR Gronland:ti,ab OR Foroyar:ti,ab OR Faeroerne:ti,ab OR 'Faroe islands':ti,ab OR Aalborg:ti,ab OR Alborg:ti,ab OR Odense:ti,ab OR Esbjerg:ti,ab OR Gentofte:ti,ab OR Gladsaxe:ti,ab OR Randers:ti,ab OR Kolding:ti,ab OR 'Estonia'/exp OR 'Estonian (citizen)'/exp OR 'Estonian (people)'/exp OR Estonia*:ti,ab OR Eesti:ti,ab OR Eestlased:ti,ab OR Eestlane:ti,ab OR Harju:ti,ab OR Harjumaa:ti,ab OR Hiiu:ti,ab OR Hiiumaa:ti,ab OR 'Ida Viru':ti,ab OR 'Ida Virumaa':ti,ab OR Jarvamaa:ti,ab OR Jarva:ti,ab OR Jogevamaa:ti,ab OR Jogeva:ti,ab OR Laanemaa:ti,ab OR Laane:ti,ab OR 'Laane Virumaa':ti,ab OR Parnu:ti,ab OR Parnumaa:ti,ab OR Polva:ti,ab OR Polvamaa:ti,ab OR Rapla:ti,ab OR Raplamaa:ti,ab OR Saare:ti,ab OR Saaremaa:ti,ab OR Tartu:ti,ab OR Tartumaa:ti,ab OR Valga:ti,ab OR Valgamaa:ti,ab OR Viljandimaa:ti,ab OR Viljandi:ti,ab OR Voru:ti,ab OR Vorumaa:ti,ab OR Tallinn:ti,ab OR Narva:ti,ab OR 'Kohtla Jarve':ti,ab OR Rakvere:ti,ab OR Maardu:ti,ab OR Sillamae:ti,ab OR Kuressaare:ti,ab OR 'Finland'/exp OR 'Finn (citizen)'/exp OR 'Finn (people)'/exp OR Finland:ti,ab OR Finnish*:ti,ab OR Finn:ti,ab OR Finns:ti,ab OR Suomi:ti,ab Suomen:ti,ab OR Suomalaiset:ti,ab OR Aland:ti,ab OR Ahvenanmaa:ti,ab OR Uusimaa:ti,ab OR Nyland:ti,ab OR Karelia:ti,ab OR Karjala:ti,ab OR Karelen:ti,ab OR Ostrobothnia:ti,ab OR Pohjanmaa:ti,ab OR Osterbotten:ti,ab OR Savonia:ti,ab OR Savo:ti,ab OR Savolax:ti,ab OR Kainuu:ti,ab OR Kajanaland*:ti,ab OR 'Kanta Hame':ti,ab OR Tavastia:ti,ab OR Tavastland:ti,ab OR Kymenlaakso:ti,ab OR Kymmenedalen:ti,ab OR Lapland:ti,ab OR Lappi:ti,ab OR Lappland:ti,ab OR 'Paijat Hame':ti,ab OR Pirkanmaa:ti,ab OR Birkaland:ti,ab OR Satakunta:ti,ab OR Satakunda:ti,ab OR Helsinki:ti,ab OR Helsingfors:ti,ab OR Espoo:ti,ab OR Esbo:ti,ab OR Tampere:ti,ab OR Tammerfors:ti,ab OR Vantaa:ti,ab OR Vanda:ti,ab OR Oulu:ti,ab OR Uleaborg:ti,ab OR Turku:ti,ab OR Abo:ti,ab OR Jyvaskyla:ti,ab OR Kuopio:ti,ab OR Lahti:ti,ab OR Lahtis:ti,ab OR Kouvola:ti,ab OR 'France'/exp OR 'Frenchman'/exp OR France:ti,ab OR French*:ti,ab OR Francais*:ti,ab OR Alsace:ti,ab OR Elsass:ti,ab OR Aquitaine:ti,ab OR Aquitania:ti,ab OR Akitania:ti,ab OR Aguiéne:ti,ab OR Auvergne:ti,ab OR Auvèrnhe:ti,ab OR Auvèrnha:ti,ab OR Normandie:ti,ab OR Normandy:ti,ab OR Normaundie:ti,ab OR Bourgogne:ti,ab OR Burgundy:ti,ab OR Bregogne:ti,ab OR Borgoégne:ti,ab OR Borgogne:ti,ab OR Brittany:ti,ab OR Breizh:ti,ab OR Bertaèyn:ti,ab OR Bretagne:ti,ab OR 'Champagne Ardenne':ti,ab OR Corse:ti,ab OR Corsica:ti,ab OR 'Franche Comte':ti,ab OR 'Frantche Comte':ti,ab OR 'Franche Comtat':ti,ab OR Guadeloupe:ti,ab OR Guyane:ti,ab OR Guiana:ti,ab OR 'Languedoc Roussillon':ti,ab OR 'Lengadoc Rosselhon':ti,ab OR 'Llenguadoc-Rossello':ti,ab OR Limousin:ti,ab OR Lemosin:ti,ab OR Lorraine:ti,ab OR Lothringen:ti,ab OR Lottringe:ti,ab OR Martinique:ti,ab OR 'Midi Pyrenees':ti,ab OR 'Miègjorn Pirenèus':ti,ab OR 'Mieidia Pirenèus':ti,ab OR 'Mediodia Pirineos':ti,ab OR 'Pays de la Loire':ti,ab OR 'Broioù al Liger':ti,ab OR Picardie:ti,ab OR Picardy:ti,ab OR 'Poitou Charentes':ti,ab OR 'Peitau Charantas':ti,ab OR 'Poetou-Cherentes':ti,ab OR Provence:ti,ab OR Provenca:ti,ab OR Prouvenco:ti,ab OR 'Cote d Azur':ti,ab OR 'Costo d Azur':ti,ab OR 'Costa d Azur':ti,ab OR Reunion:ti,ab OR 'Rhone Alpes':ti,ab OR 'Rono Arpes':ti,ab OR 'Rose Aups':ti,ab OR Ain:ti,ab OR Aisne:ti,ab OR Allier:ti,ab OR 'Alpes de Haute Provence':ti,ab OR 'Haute Alpes':ti,ab OR 'Alpes Maritimes':ti,ab OR Ardeche:ti,ab OR Ardennes:ti,ab OR Ariege:ti,ab OR Aube:ti,ab OR Aude:ti,ab OR Aveyron:ti,ab OR 'Bas Rhin':ti,ab OR 'Bouches du Rhone':ti,ab OR Calvados:ti,ab OR Cantal:ti,ab OR Charente:ti,ab OR Cher:ti,ab OR Correze:ti,ab OR 'Corse du Sud':ti,ab OR 'Cote d Or':ti,ab OR 'Cotes d Armor':ti,ab OR Creuse:ti,ab OR 'Deux Sevres':ti,ab OR Dordogne:ti,ab OR Doubs:ti,ab OR Drome:ti,ab OR Essonne:ti,ab OR Eure:ti,ab OR Finistere:ti,ab OR Gard:ti,ab OR Gers:ti,ab OR Gironde:ti,ab OR 'Haute Corse':ti,ab OR 'Haute Garonne':ti,ab OR 'Haute Marne':ti,ab OR 'Hautes Alpes':ti,ab OR 'Haute Saone':ti,ab OR 'Haute Savoie':ti,ab OR 'Hautes Pyrenees':ti,ab OR 'Haute Vienne':ti,ab OR 'Haut Rhin':ti,ab OR 'Hauts de Seine':ti,ab OR Herault:ti,ab OR 'Ile de France':ti,ab OR 'Ille et Vilaine':ti,ab OR Indre:ti,ab OR Isere:ti,ab OR Jura:ti,ab OR Landes:ti,ab OR Loire:ti,ab OR Loiret:ti,ab OR (Lot NEAR/3 (departement OR department)):ab,ti OR 'Lot et Garonne':ti,ab OR 'Loir et Cher':ti,ab OR Lozere:ti,ab OR Manche:ti,ab OR Marne:ti,ab OR Mayenne:ti,ab OR Mayotte:ti,ab OR 'Meurthe et Moselle':ti,ab OR Meuse:ti,ab OR Morbihan:ti,ab OR Moselle:ti,ab OR (Nord NEAR/3 (department OR departement)):ti,ab OR Nievre:ti,ab OR Oise:ti,ab OR Orne:ti,ab OR 'Pas de calais':ti,ab OR 'Noord-Nauw van Kales':ti,ab OR Paris:ti,ab OR 'Puy de dome':ti,ab OR 'Pyrenees Atlantiques':ti,ab OR 'Pyrenees Orientales':ti,ab OR Rhone:ti,ab OR Sarthe:ti,ab OR Savoie:ti,ab OR 'Seine et Marne':ti,ab OR 'Seine Maritime':ti,ab OR Somme:ti,ab OR Tarn:ti,ab OR 'Territoire de Belfort':ti,ab OR 'Val de Marne':ti,ab OR 'Val d Oise':ti,ab OR Var:ti,ab OR Vaucluse:ti,ab OR Vendee:ti,ab OR Vienne:ti,ab OR Vosges:ti,ab OR Yonne:ti,ab OR Yvelines:ti,ab OR Marseille:ti,ab OR Lyon:ti,ab OR Nice:ti,ab OR Nantes:ti,ab OR Strasbourg:ti,ab OR Montpellier:ti,ab OR Bordeaux:ti,ab OR Lille:ti,ab OR Toulouse:ti,ab OR 'Outre Mer':ti,ab OR 'Seine Saint Denis':ti,ab OR 'Germany'/de OR 'German (citizen)'/exp OR German*:ti,ab OR Deutsch*:ti,ab OR Bundesrepublik:ti,ab OR Westdeutschland:ti,ab OR Ostdeutschland:ti,ab OR Baden:ti,ab OR Wuerttemberg:ti,ab OR Wurttemberg:ti,ab OR Bayern:ti,ab OR Bavaria:ti,ab OR Berlin:ti,ab OR Brandenburg:ti,ab OR Bremen:ti,ab OR Oldenburg:ti,ab OR Mitteldeutschland:ti,ab OR Rhein:ti,ab OR Rhine:ti,ab OR Hannover:ti,ab OR Braunschweig:ti,ab OR Göttingen:ti,ab OR Goettingen:ti,ab OR Nurnberg:ti,ab OR Nuernberg:ti,ab OR Ruhr:ti,ab OR Koln:ti,ab OR koeln:ti,ab OR Bonn:ti,ab OR Hamburg:ti,ab OR Hessen:ti,ab OR Hesse:ti,ab OR Hessia:ti,ab OR Mecklenburg:ti,ab OR Vorpommern:ti,ab OR Pomerania:ti,ab OR Niedersachsen:ti,ab OR Neddersassen:ti,ab OR Saxony:ti,ab OR Niederbayern:ti,ab OR 'Northern Rhine':ti,ab OR 'North Rhine':ti,ab OR Westphalia:ti,ab OR Westfalen:ti,ab OR 'Rhineland Palatinate':ti,ab OR 'Rheinland Pfalz':ti,ab OR Saarland:ti,ab OR Sachsen:ti,ab OR 'Schleswig Holstein':ti,ab OR Thuringia:ti,ab OR Thuringen:ti,ab OR Thueringen:ti,ab OR Munchen:ti,ab OR Muenchen:ti,ab OR Munich:ti,ab OR Frankfurt:ti,ab OR Stuttgart:ti,ab OR Dusseldorf:ti,ab OR Duesseldorf:ti,ab OR Dortmund:ti,ab OR Essen:ti,ab OR 'Greece'/exp OR 'Greek (citizen)'/exp OR 'Greek (people)'/exp OR Greece:ti,ab OR 'Hellenic republic':ti,ab OR Greek*:ti,ab OR Ellada:ti,ab OR Elladas:ti,ab OR 'Elliniki Dimokratia':ti,ab OR Hellas:ti,ab OR Hellenes:ti,ab OR Attica:ti,ab OR Attiki:ti,ab OR Makedonia*:ti,ab OR Macedonia:ti,ab OR Thraki:ti,ab OR Thrace:ti,ab OR Crete:ti,ab OR Kriti:ti,ab OR 'Ionia Nisia':ti,ab OR 'Ionion neson':ti,ab OR 'Ionion nIson':ti,ab OR 'Ionian islands':ti,ab OR 'Ionian island':ti,ab OR Epirus:ti,ab OR Ipeiros:ti,ab OR 'Periféreia Ipeírou':ti,ab OR 'North aegean':ti,ab OR 'Northern Aegean':ti,ab OR 'Aegean islands':ti,ab OR 'Aegean island':ti,ab OR 'Nisoi Agaiou':ti,ab OR 'Notio Aigaio':ti,ab OR Peloponnese:ti,ab OR Peloponniso*:ti,ab OR Thessaly:ti,ab OR Thessalia:ti,ab OR Thessalian:ti,ab OR Petthalia:ti,ab OR 'Voreio Aigaio':ti,ab OR 'Voreio Aigaiou':ti,ab OR 'South aegean':ti,ab OR 'Southern Aegean':ti,ab OR 'Mount athos':ti,ab OR 'Oros Athos':ti,ab OR Cyclades:ti,ab OR Cycklades:ti,ab OR Kiklades:ti,ab OR Dodecanese:ti,ab OR Dodekanisa:ti,ab OR Athens:ti,ab OR Athina:ti,ab OR Thessaloniki:ti,ab OR Thessalonica:ti,ab OR Patras:ti,ab OR Patra:ti,ab OR Pireas:ti,ab OR Piraeus:ti,ab OR Larissa:ti,ab OR Larisa:ti,ab OR Heraklion:ti,ab OR Heraclion:ti,ab OR Iraklion:ti,ab OR Irakleion:ti,ab OR Iraklio:ti,ab OR Volos:ti,ab OR Rhodes:ti,ab OR Rodos:ti,ab OR Ioannina:ti,ab OR Janina:ti,ab OR Yannena:ti,ab OR Chania:ti,ab OR Chalcis:ti,ab OR Chalkida:ti,ab OR 'Hungary'/exp OR 'Hungarian (citizen)'/exp OR 'Magyar (people)'/exp OR Hungar*:ti,ab OR Magyarorszag:ti,ab OR Magyar*:ti,ab OR Dunantuli:ti,ab OR Transdanubia:ti,ab OR Dunantul:ti,ab OR 'Great Plain':ti,ab OR 'Eszak Alfold':ti,ab OR 'Del Alfold':ti,ab OR 'Alfold es eszak':ti,ab OR 'Northern Alfold':ti,ab OR 'North Alfold':ti,ab OR 'South Alfold':ti,ab OR 'Southern Alfold':ti,ab OR Bacs:ti,ab OR Kiskun:ti,ab OR Baranya:ti,ab OR Bekes:ti,ab OR Borsod:ti,ab OR Abauj:ti,ab OR Zemplen:ti,ab OR Budapest:ti,ab OR Csongrad:ti,ab OR Fejer:ti,ab OR gyor:ti,ab OR moson:ti,ab OR sopron:ti,ab OR hajdu:ti,ab OR bihar:ti,ab OR Heves:ti,ab OR 'jasz nagykun szolnok':ti,ab OR komarom:ti,ab OR esztergom:ti,ab OR Nograd:ti,ab OR (Pest NEXT/3 (megye OR county)):ti,ab OR Somogy:ti,ab OR szabolcs:ti,ab OR szatmar:ti,ab OR bereg:ti,ab OR Tolna:ti,ab OR Vas:ti,ab OR Veszprem:ti,ab OR Zala:ti,ab OR Debrecen:ti,ab OR Miskolc:ti,ab OR Szeged:ti,ab OR Pecs:ti,ab OR Gyor:ti,ab OR Nyiregyhaza:ti,ab OR Kecskemet:ti,ab OR Szekesfehervar:ti,ab OR Szombathely:ti,ab OR 'Ireland'/exp OR 'Irish (citizen)'/exp OR Ireland:ti,ab OR Eire:ti,ab OR Irish*:ti,ab OR Fingal:ti,ab OR 'Fine Gall':ti,ab OR Dublin:ti,ab OR 'Ath Cliath':ti,ab OR 'Dun Laoghaire':ti,ab OR Wicklow:ti,ab OR 'Cill Mhantain':ti,ab OR 'Chill Mhantain':ti,ab OR Wexford:ti,ab OR 'Loch Garman':ti,ab OR Carlow:ti,ab OR Ceatharlach:ti,ab OR Kildare:ti,ab OR 'Cill Dara':ti,ab OR 'Chill Dara':ti,ab OR Meath:ti,ab OR 'An Mhi':ti,ab OR 'Contae na Mi':ti,ab OR Louth:ti,ab OR 'Contae Lu':ti,ab OR Monaghan:ti,ab OR Muineachán:ti,ab OR Mhuineacháin:ti,ab OR Cavan:ti,ab OR 'An Cabhan':ti,ab OR 'An Cabhain':ti,ab OR Longford:ti,ab OR 'An Longfort':ti,ab OR 'an Longfoirt':ti,ab OR Langfurd:ti,ab OR Westmeath:ti,ab OR 'An Iarmhi':ti,ab OR 'na Iarmhi':ti,ab OR Offaly:ti,ab OR 'Uibh Fhaili':ti,ab OR Laois:ti,ab OR Laoise:ti,ab OR Kilkenny:ti,ab OR 'Chill Chainnigh':ti,ab OR 'Cill Chainnigh':ti,ab OR Waterford:ti,ab OR 'Port Lairge':ti,ab OR Watterford:ti,ab OR Cork:ti,ab OR Corcaigh:ti,ab OR Chorcai:ti,ab OR Kerry:ti,ab OR Ciarrai:ti,ab OR Chiarrai:ti,ab OR Limerick:ti,ab OR Luimneach:ti,ab OR Luimnigh:ti,ab OR Tipperary:ti,ab OR 'Tiobraid Arann':ti,ab OR 'Thiobraid Arann':ti,ab OR Clare:ti,ab OR 'An Clar':ti,ab OR 'an Chlair':ti,ab OR Galway:ti,ab OR Gaillimh:ti,ab OR 'na Gaillimhe':ti,ab OR Mayo:ti,ab OR 'Maigh Eo':ti,ab OR 'Mhaigh Eo':ti,ab OR Roscommon:ti,ab OR 'Ros comain':ti,ab OR Sligo:ti,ab OR Sligeach:ti,ab OR Shligigh:ti,ab OR Leitrim:ti,ab OR Liatroim:ti,ab OR Liatroma:ti,ab OR Donegal:ti,ab OR 'Dhún na nGall':ti,ab OR Dinnygal:ti,ab OR Dunnyga:ti,ab OR Leinster:ti,ab OR Laighin:ti,ab OR 'Cúige Laighean':ti,ab OR Munster:ti,ab OR Mumhain:ti,ab OR 'Cúige Mumhan':ti,ab OR Connacht:ti,ab OR Connachta:ti,ab OR Drogheda:ti,ab OR 'Droichead Atha':ti,ab OR Dundalk:ti,ab OR 'Dún Dealgan':ti,ab OR Swords:ti,ab OR Sord:ti,ab OR Bray:ti,ab OR Bre:ti,ab OR Navan:ti,ab OR 'An Uaimh':ti,ab OR 'Italy'/exp OR 'Italian (citizen)'/exp OR 'Italic people'/exp OR Italy:ti,ab OR Italia*:ti,ab OR Abruzzo:ti,ab OR Abruzzi:ti,ab OR Basilicata:ti,ab OR Lucania:ti,ab OR Calabria:ti,ab OR Campania:ti,ab OR 'Emilia Romagna':ti,ab OR 'friuli venezia giulia':ti,ab OR Lazio:ti,ab OR Latium:ti,ab OR Liguria*:ti,ab OR Lombardy:ti,ab OR Lombardia:ti,ab OR Marche:ti,ab OR Marches:ti,ab OR Molisano:ti,ab OR Molise:ti,ab OR Piedmont*:ti,ab OR Piemonte:ti,ab OR Bolzano:ti,ab OR Bozen:ti,ab OR Trentino:ti,ab OR Trento:ti,ab OR Puglia:ti,ab OR Apulia:ti,ab OR Sardinia:ti,ab OR Sardegna:ti,ab OR Sicily:ti,ab OR Sicilia:ti,ab OR Toscana:ti,ab OR Tuscany:ti,ab OR Umbria:ti,ab OR 'Valle d Aosta':ti,ab OR 'Vallee d Aoste':ti,ab OR 'Aosta Valley':ti,ab OR Veneto:ti,ab OR Venetia:ti,ab OR Triveneto:ti,ab OR Rome:ti,ab OR Roma:ti,ab OR Milan:ti,ab OR Milano:ti,ab OR Naples:ti,ab OR Napoli:ti,ab OR Turin:ti,ab OR Torino:ti,ab OR Palermo:ti,ab OR Genoa:ti,ab OR Genova:ti,ab OR Bologna:ti,ab OR Florence:ti,ab OR Firenze:ti,ab OR Bari:ti,ab OR Catania:ti,ab OR 'Latvia'/exp OR 'Latvian (citizen)'/exp OR 'Lett (people)'/exp OR Latvi*:ti,ab OR Riga:ti,ab OR Courland:ti,ab OR Kurzeme:ti,ab OR Kurland:ti,ab OR Latgale:ti,ab OR Lettgallia:ti,ab OR Latgola:ti,ab OR Latgalia:ti,ab OR Vidzeme:ti,ab OR Vidumo:ti,ab OR Semigallia:ti,ab OR Semigalia:ti,ab OR Zemgale:ti,ab OR Pieriga:ti,ab OR Daugavpils:ti,ab OR Dinaburg:ti,ab OR Jekabpils:ti,ab OR Jakobstadt:ti,ab OR Jelgava:ti,ab OR Jurmala:ti,ab OR Liepaja:ti,ab OR Libau:ti,ab OR Rezekne:ti,ab OR Rezne:ti,ab OR Rositten:ti,ab OR Valmiera:ti,ab OR Wolmar:ti,ab OR Ventspils:ti,ab OR Windau:ti,ab OR Ogre:ti,ab OR 'Lithuania'/exp OR 'Lithuanian (citizen)'/exp OR Lithuania*:ti,ab OR 'Lietuvos Respublika':ti,ab OR Lietuva:ti,ab OR lietuviu:ti,ab OR Alytus:ti,ab OR Alytaus:ti,ab OR Kaunas:ti,ab OR Kauno:ti,ab OR Klaipeda:ti,ab OR Klaipedos:ti,ab OR Marijampoles:ti,ab OR Marijampole:ti,ab OR Panevezys:ti,ab OR Panevezio:ti,ab OR Siauliai:ti,ab OR Siauliu:ti,ab OR Taurages:ti,ab OR Taurage:ti,ab OR Telsiu:ti,ab OR Telsiai:ti,ab OR Utenos:ti,ab OR Utena:ti,ab OR Vilnius:ti,ab OR Vilniaus:ti,ab OR Mazeikiai:ti,ab OR Jonava:ti,ab OR Mazeikiu:ti,ab OR Jonavos:ti,ab OR 'Luxembourg'/exp OR Luxembourg*:ti,ab OR Luxemburg:ti,ab OR Letzebuerg:ti,ab OR Diekirch:ti,ab OR Grevenmacher:ti,ab OR 'Esch sur Alzette':ti,ab OR 'Esch Uelzecht':ti,ab OR 'Esch an der Alzette':ti,ab OR 'Esch an der Alzig':ti,ab OR Dudelange:ti,ab OR Diddeleng:ti,ab OR Düdelingen:ti,ab OR Duedelingen:ti,ab OR Schifflange:ti,ab OR Scheffleng:ti,ab OR Schifflingen:ti,ab OR Bettembourg:ti,ab OR Beetebuerg:ti,ab OR Bettemburg:ti,ab OR Petange:ti,ab OR Peiteng:ti,ab OR Petingen:ti,ab OR Ettelbruck:ti,ab OR Ettelbreck:ti,ab OR Ettelbrueck:ti,ab OR Diekirch:ti,ab OR Dikrech:ti,ab OR Strassen:ti,ab OR Stroossen:ti,ab OR Bertrange:ti,ab OR Bartreng:ti,ab OR Bartringen:ti,ab OR 'Malta'/exp OR 'Maltese (citizen)'/exp OR Malta:ti,ab OR Maltese*:ti,ab OR Maltin:ti,ab OR Gozo:ti,ab OR Ghawdex:ti,ab OR Valletta:ti,ab OR 'Ill Belt':ti,ab OR Birkirkara:ti,ab OR 'B Kara':ti,ab OR Birchircara:ti,ab OR Mosta:ti,ab OR Qormi:ti,ab OR 'St Paul s Bay':ti,ab OR 'Pawl il Bahar':ti,ab OR Zabbar:ti,ab OR Sliema:ti,ab OR Naxxar:ti,ab OR Gwann:ti,ab OR 'St John':ti,ab OR Zebbug:ti,ab OR 'Citta rohan':ti,ab OR Fgura:ti,ab OR 'Netherlands'/exp OR 'Dutchman'/exp OR Netherlands:ti,ab OR Nederland*:ti,ab OR Dutch*:ti,ab OR Drenthe:ti,ab OR Flevoland:ti,ab OR Friesland:ti,ab OR Fryslan:ti,ab OR Frisia:ti,ab OR Gelderland:ti,ab OR Guelders:ti,ab OR Groningen:ti,ab OR Limburg:ti,ab OR Brabant:ti,ab OR Holland:ti,ab OR Overijssel:ti,ab OR Overissel:ti,ab OR Utrecht:ti,ab OR Zeeland:ti,ab OR Amsterdam:ti,ab OR Rotterdam:ti,ab OR Hague:ti,ab OR 's-Gravenhage':ti,ab OR 'Den Haag':ti,ab OR Eindhoven:ti,ab OR Tilburg:ti,ab OR Almere:ti,ab OR Breda:ti,ab OR Nijmegen:ti,ab OR Nimeguen:ti,ab OR 'Poland'/exp OR 'Polish citizen'/exp OR 'Pole (people)'/exp OR Poland:ti,ab OR Polska:ti,ab OR Polish:ti,ab OR Pole:ti,ab OR Poles:ti,ab OR Polski:ti,ab OR Polak:ti,ab OR Polka:ti,ab OR Polacy:ti,ab OR Dolnoslaskie:ti,ab OR Silesia*:ti,ab OR Slask:ti,ab OR Pomorskie:ti,ab OR Pomerania*:ti,ab OR Kujawsko:ti,ab OR Kuyavian:ti,ab OR Lodzkie:ti,ab OR Lodz:ti,ab OR Lubelskie:ti,ab OR Lublin:ti,ab OR Lubuskie:ti,ab OR Lubusz:ti,ab OR Lubus:ti,ab OR Malopolskie:ti,ab OR Mazowieckie:ti,ab OR Mazowske:ti,ab OR Masovia:ti,ab OR Masovian:ti,ab OR Opolskie:ti,ab OR Opole:ti,ab OR Podkarpackie:ti,ab OR Subcarpathian*:ti,ab OR Podlaskie:ti,ab OR Podlachia:ti,ab OR Podlasie:ti,ab OR Slaskie:ti,ab OR Swietokrzyskie:ti,ab OR 'Varmia Mazuria':ti,ab OR 'Varmian Mazurian':ti,ab OR 'Varmia Masuria':ti,ab OR 'Varmian Masurian':ti,ab OR 'Warmia Mazury':ti,ab OR 'Warminsko Mazurskie':ti,ab OR 'Warmian Masurian':ti,ab OR Wielkopolskie:ti,ab OR Zachodniopomorskie:ti,ab OR Warsaw:ti,ab OR Warszawa:ti,ab OR Krakow:ti,ab OR Cracow:ti,ab OR Wroclaw:ti,ab OR Poznan:ti,ab OR Gdansk:ti,ab OR Szczecin:ti,ab OR Bydgoszcz:ti,ab OR Katowice:ti,ab OR 'Portugal'/exp OR 'Portuguese (citizen)'/exp OR Portugal:ti,ab OR Portugues*:ti,ab OR Azores:ti,ab OR Acores:ti,ab OR Madeira:ti,ab OR Alentejo:ti,ab OR Algarve:ti,ab OR Lisboa:ti,ab OR Lisbon:ti,ab OR 'Alto Tras-os-Montes':ti,ab OR (Ave NEAR/3 (community OR intermunicipal OR comunidade)):ti,ab OR Mondego:ti,ab OR Vouga:ti,ab OR Beira:ti,ab OR Cavado:ti,ab OR Lafoes:ti,ab OR Douro:ti,ab OR Porto:ti,ab OR Oporto:ti,ab OR Tejo:ti,ab OR Minho:ti,ab OR Setubal:ti,ab OR Pinhal:ti,ab OR 'Serra da Estrela':ti,ab OR Tamega:ti,ab OR Leira:ti,ab OR Santarem:ti,ab OR Beja:ti,ab OR Faro:ti,ab OR Evora:ti,ab OR Portalegre:ti,ab OR 'Castelo Branco':ti,ab OR Guarda:ti,ab OR Cimbra:ti,ab OR Aveiro:ti,ab OR Viseu:ti,ab OR Braganca:ti,ab OR Braganza:ti,ab OR Braga:ti,ab OR 'Vila real':ti,ab OR 'Viana do Castelo':ti,ab OR Gaia:ti,ab OR Amadora:ti,ab OR Funchal:ti,ab OR Coimbra:ti,ab OR Almada:ti,ab OR (Agualva:ti,ab AND Cacem:ti,ab) OR 'Romania'/exp OR 'Romanian (citizen)'/exp OR Romania*:ti,ab OR Rumania*:ti,ab OR Roumania*:ti,ab OR Romani:ti,ab OR Rumani:ti,ab OR Alba:ti,ab OR Arad:ti,ab OR Arges:ti,ab OR Bacau:ti,ab OR Bihor:ti,ab OR 'Bistrita Nasaud':ti,ab OR Botosani:ti,ab OR Braila:ti,ab OR Brasov:ti,ab OR Kronstadt:ti,ab OR Brasso:ti,ab OR Brassovia:ti,ab OR Coron:ti,ab OR Bucharest:ti,ab OR Bucuresti:ti,ab OR Buzau:ti,ab OR Calarasi:ti,ab OR 'Caras-Severin':ti,ab OR Cluj:ti,ab OR Klausenburg:ti,ab OR Kolozsvar:ti,ab OR Constanta:ti,ab OR Tomis:ti,ab OR Konstantia:ti,ab OR Kostence:ti,ab OR Covasna:ti,ab OR Dambovita:ti,ab OR Dolj:ti,ab OR Galati:ti,ab OR Galatz:ti,ab OR Galac:ti,ab OR Kalas:ti,ab OR Giurgiu:ti,ab OR Gorj:ti,ab OR Harghita:ti,ab OR Hunedoara:ti,ab OR Ialomita:ti,ab OR Iasi:ti,ab OR Jassy:ti,ab OR Lassy:ti,ab OR Ilfov:ti,ab OR Maramures:ti,ab OR Mehedinti:ti,ab OR Mures:ti,ab OR Neamt:ti,ab OR (Olt:ti,ab AND (river:ti,ab OR county:ti,ab OR region:ti,ab OR judetul:ti,ab OR Raul:ti,ab)) OR Prahova:ti,ab OR Salaj:ti,ab OR 'Satu Mare':ti,ab OR Sibiu:ti,ab OR Suceava:ti,ab OR Teleorman:ti,ab OR Timis:ti,ab OR Tulcea:ti,ab OR Valcea:ti,ab OR Vilcea:ti,ab OR Vaslui:ti,ab OR Vrancea:ti,ab OR Timisoara:ti,ab OR Temeswar:ti,ab OR Temeschburg:ti,ab OR Temeschwar:ti,ab OR Temesvar:ti,ab OR Temisvar:ti,ab OR Timisvar:ti,ab OR Temesva:ti,ab OR Craiova:ti,ab OR Ploiesti:ti,ab OR Ploesti:ti,ab OR Oradea:ti,ab OR Varad:ti,ab OR Varat:ti,ab OR 'Slovakia'/exp OR 'Slovak (citizen)'/exp OR 'Slovak (people)'/exp OR Slovakia:ti,ab OR Slovensk*:ti,ab OR Slovak*:ti,ab OR Slovaci:ti,ab OR Slovenki:ti,ab OR Bratislav*:ti,ab OR Presporok:ti,ab OR Pressburg:ti,ab OR Preßburg:ti,ab OR Posonium:ti,ab OR Banskobystri*:ti,ab OR 'Banska Bystrica':ti,ab OR Neusohl:ti,ab OR Besztercebánya:ti,ab OR Kosic*:ti,ab OR Kaschau:ti,ab OR Kassa:ti,ab OR Nitrian*:ti,ab OR Nitra:ti,ab OR Neutra:ti,ab OR Nyitra:ti,ab OR Nyitria:ti,ab OR Trnav*:ti,ab OR Tyrnau:ti,ab OR Nagyszombat:ti,ab OR Tyrnavia:ti,ab OR Presov*:ti,ab OR Trencian*:ti,ab OR Trencin:ti,ab OR Trentschin:ti,ab OR Trencsén:ti,ab OR Zilina:ti,ab OR Sillein:ti,ab OR Zsolna:ti,ab OR Zylina:ti,ab OR (Martin:ti,ab AND (city:ti,ab OR Svaty:ti,ab)) OR Turócszentmárton:ti,ab OR Poprad:ti,ab OR Deutschendorf:ti,ab OR Zvolen:ti,ab OR 'Slovenia'/exp OR 'Slovenian (citizen)'/exp OR 'Slovene (people)'/exp OR Slovenia*:ti,ab OR Slovenija:ti,ab OR slovensk*:ti,ab OR Slovenci:ti,ab OR Slovene*:ti,ab OR Gorenjska:ti,ab OR Carniola:ti,ab OR Goriska:ti,ab OR Gorizia:ti,ab OR Jugovzhodna:ti,ab OR Koroska:ti,ab OR Carinthia:ti,ab OR 'Notranjsko kraska':ti,ab OR 'Obalno kraska':ti,ab OR 'Coastal karst':ti,ab OR Osrednjeslovenska:ti,ab OR Podravska:ti,ab OR Drava:ti,ab OR Pomurska:ti,ab OR Mura:ti,ab OR Savinjska:ti,ab OR Savinja:ti,ab OR Spodnjeposavska:ti,ab OR Zasavska:ti,ab OR 'Central Sava':ti,ab OR Posavska:ti,ab OR 'Lower Sava':ti,ab OR Ljubljana:ti,ab OR Laibach:ti,ab OR Lubiana:ti,ab OR Maribor:ti,ab OR 'Marburg an der Drau':ti,ab OR Kranj:ti,ab OR Carnium:ti,ab OR Creina:ti,ab OR Chreina:ti,ab OR Krainbur:ti,ab OR Koper:ti,ab OR Capodistria:ti,ab OR Kopar:ti,ab OR Celje:ti,ab OR 'Novo mesto':ti,ab OR Neustadtl:ti,ab OR Domzale:ti,ab OR Velenje:ti,ab OR Wollan:ti,ab OR Woellan:ti,ab OR 'Nova Gorica':ti,ab OR Kamnik:ti,ab OR 'Spain'/exp OR 'Spaniard'/exp OR 'Basque (people)'/exp OR Spain:ti,ab OR Espana:ti,ab OR Spanish:ti,ab OR Espanol*:ti,ab OR Spaniard*:ti,ab OR Andalucia:ti,ab OR Andalusia:ti,ab OR Aragon:ti,ab OR Arago:ti,ab OR Cantabria:ti,ab OR Canarias:ti,ab OR 'Canary Islands':ti,ab OR (Canaries:ti,ab AND island*:ti,ab) OR 'Castile and leon':ti,ab OR 'Castilla y Leon':ti,ab OR 'Castile La Mancha':ti,ab OR 'Castilla La Mancha':ti,ab OR Cataluna:ti,ab OR Catalonia:ti,ab OR Ceuta:ti,ab OR Madrid:ti,ab OR Melilla:ti,ab OR Navarra:ti,ab OR Navarre:ti,ab OR Valencia*:ti,ab OR Extremadura:ti,ab OR Galicia:ti,ab OR Balears:ti,ab OR 'Balearic Islands':ti,ab OR 'Balear Islands':ti,ab OR Baleares:ti,ab OR 'La Rioja':ti,ab OR 'Pais Vasco':ti,ab OR 'Basque Country':ti,ab OR 'Baske region':ti,ab OR Euskadi:ti,ab OR Asturias:ti,ab OR Murcia:ti,ab OR Coruna:ti,ab OR Alava:ti,ab OR Araba:ti,ab OR Albacete:ti,ab OR Alicante:ti,ab OR Alacant:ti,ab OR Almeria:ti,ab OR Avila:ti,ab OR Badajoz:ti,ab OR Badajos:ti,ab OR Barcelona:ti,ab OR Burgos:ti,ab OR Caceres:ti,ab OR Cadiz:ti,ab OR Castellon:ti,ab OR Castello:ti,ab OR 'Ciudad Real':ti,ab OR Cordoba:ti,ab OR Cuenca:ti,ab OR Eivissa:ti,ab OR Ibiza:ti,ab OR Formentera:ti,ab OR 'El Hierro':ti,ab OR Fuerteventura:ti,ab OR Galiza:ti,ab OR Girona:ti,ab OR Gerona:ti,ab OR 'Gran Canaria':ti,ab OR Granada:ti,ab OR Guadalajara:ti,ab OR Guipuzcoa:ti,ab OR Gipuzkoa:ti,ab OR Huelva:ti,ab OR Huesca:ti,ab OR Jaen:ti,ab OR 'La Gomera':ti,ab OR 'La Palma':ti,ab OR Lanzarote:ti,ab OR Leon:ti,ab OR Lleida:ti,ab OR Lerida:ti,ab OR Lugo:ti,ab OR Malaga:ti,ab OR Mallorca:ti,ab OR Majorca:ti,ab OR Menorca:ti,ab OR Minorca:ti,ab OR Murcia:ti,ab OR Ourense:ti,ab OR Orense:ti,ab OR Palencia:ti,ab OR Pontevedra:ti,ab OR Salamanca:ti,ab OR Segovia:ti,ab OR Sevilla:ti,ab OR Seville:ti,ab OR Soria:ti,ab OR Tarragona:ti,ab OR Tenerife:ti,ab OR Teruel:ti,ab OR Toledo:ti,ab OR Valladolid:ti,ab OR Vizcaya:ti,ab OR Biscay:ti,ab OR Zamora:ti,ab OR Zaragoza:ti,ab OR Saragossa:ti,ab OR 'Las Palmas':ti,ab OR Bilbao:ti,ab OR Bilbo:ti,ab OR 'Sweden'/exp OR 'Swedish citizen'/exp OR 'Swede (people)'/exp OR Sweden:ti,ab OR Sverige:ti,ab OR Swedish:ti,ab OR Svenska:ti,ab OR svenskar:ti,ab OR Swede:ti,ab OR Swedes:ti,ab OR Norrland:ti,ab OR Mellansverige:ti,ab OR Smaland:ti,ab OR Stockholm*:ti,ab OR Sydsverige:ti,ab OR Vastsverige:ti,ab OR Blekinge:ti,ab OR Dalarna:ti,ab OR Gavleborg*:ti,ab OR Gotland*:ti,ab OR Halland*:ti,ab OR Jamtland*:ti,ab OR Jonkoping*:ti,ab OR Kalmar:ti,ab OR Kronoberg*:ti,ab OR Norrbotten*:ti,ab OR Orebro:ti,ab OR Ostergotland*:ti,ab OR Skane:ti,ab OR Sodermanlands:ti,ab OR Uppsala:ti,ab OR Varmland*:ti,ab OR Vasterbotten*:ti,ab OR Vasternorrland*:ti,ab OR Vastmanland*:ti,ab OR vastergotland*:ti,ab OR Gotaland*:ti,ab OR Gothenburg:ti,ab OR Goteborg:ti,ab OR Malmo:ti,ab OR Vasteras:ti,ab OR Linkoping:ti,ab OR Helsingborg:ti,ab OR Halsingborg:ti,ab OR Norrkoping:ti,ab OR 'United Kingdom'/exp OR 'British citizen'/exp OR 'GB':ti,ab OR 'United kingdom':ti,ab OR 'UK':ti,ab OR Britain:ti,ab OR British:ti,ab OR England:ti,ab OR English:ti,ab OR Scotland:ti,ab OR Scottish:ti,ab OR Scots:ti,ab OR Wales:ti,ab OR Cymru:ti,ab OR Welsh:ti,ab OR 'North Ireland':ti,ab OR 'Northern Ireland':ti,ab OR Irish:ti,ab OR Avon:ti,ab OR Bedfordshire:ti,ab OR Berkshire:ti,ab OR Bristol:ti,ab OR Buckinghamshire:ti,ab OR Cambridgeshire:ti,ab OR 'Isle of Ely':ti,ab OR Cheshire:ti,ab OR Cleveland:ti,ab OR Cornwall:ti,ab OR Cumberland:ti,ab OR Cumbria:ti,ab OR Derbyshire:ti,ab OR Devon:ti,ab OR Dorset:ti,ab OR Durham:ti,ab OR Essex:ti,ab OR Gloucestershire:ti,ab OR Hampshire:ti,ab OR Southampton:ti,ab OR (Hereford:ti,ab AND Worcester:ti,ab) OR Hertfordshire:ti,ab OR Herefordshire:ti,ab OR Humberside:ti,ab OR Huntingdon:ti,ab OR Huntingdonshire:ti,ab OR 'Isle of Wight':ti,ab OR Kent:ti,ab OR Lancashire:ti,ab OR Leicestershire:ti,ab OR Lincolnshire:ti,ab OR London:ti,ab OR Manchester:ti,ab OR Merseyside:ti,ab OR Middlesex:ti,ab OR Norfolk:ti,ab OR Northamptonshire:ti,ab OR Northumberland:ti,ab OR Nottinghamshire:ti,ab OR Oxfordshire:ti,ab OR Peterborough:ti,ab OR Rutland:ti,ab OR Shropshire:ti,ab OR Salop:ti,ab OR Somerset:ti,ab OR Yorkshire:ti,ab OR Staffordshire:ti,ab OR Suffolk:ti,ab OR Surrey:ti,ab OR Sussex:ti,ab OR (Tyne:ti,ab AND Wear:ti,ab) OR Warwickshire:ti,ab OR Midlands:ti,ab OR Westmorland:ti,ab OR Wiltshire:ti,ab OR Worcestershire:ti,ab OR 'Isle of Man':ti,ab OR Jersey:ti,ab OR Guernsey:ti,ab OR 'Channel Islands':ti,ab OR Aberdeen:ti,ab OR Aberdeenshire:ti,ab OR Angus:ti,ab OR Forfarshire:ti,ab OR Argyll:ti,ab OR Ayrshire:ti,ab OR Banffshire:ti,ab OR Berwickshire:ti,ab OR Bute:ti,ab OR Caithness:ti,ab OR Clackmannanshire:ti,ab OR Cromartyshire:ti,ab OR Dumfriesshire:ti,ab OR Dunbartonshire:ti,ab OR Dumbarton:ti,ab OR Dundee:ti,ab OR Lothian:ti,ab OR Haddingtonshire:ti,ab OR Edinburgh:ti,ab OR Fife:ti,ab OR Glasgow:ti,ab OR Inverness-shire:ti,ab OR Kincardineshire:ti,ab OR Kinross-shire:ti,ab OR Kirkcudbrightshire:ti,ab OR Lanarkshire:ti,ab OR Midlothian:ti,ab OR Moray:ti,ab OR Elginshire:ti,ab OR Nairnshire:ti,ab OR Orkney:ti,ab OR Peeblesshire:ti,ab OR Perthshire:ti,ab OR Renfrewshire:ti,ab OR (Ross:ti,ab AND Cromarty:ti,ab) OR Ross-shire:ti,ab OR Roxburghshire:ti,ab OR Selkirkshire:ti,ab OR Shetland:ti,ab OR Zetland:ti,ab OR Stirlingshire:ti,ab OR Sutherland:ti,ab OR Linlithgowshire:ti,ab OR Wigtownshire:ti,ab OR Anglesey:ti,ab OR Brecknockshire:ti,ab OR Caernarfonshire:ti,ab OR Carmarthenshire:ti,ab OR Cardiganshire:ti,ab OR Ceredigion:ti,ab OR Clwyd:ti,ab OR Denbighshire:ti,ab OR Dyfed:ti,ab OR Flintshire:ti,ab OR Glamorgan:ti,ab OR Gwent:ti,ab OR Gwynedd:ti,ab OR Merionethshire:ti,ab OR Montgomeryshire:ti,ab OR Monmouthshire:ti,ab OR Pembrokeshire:ti,ab OR Powys:ti,ab OR Radnorshire:ti,ab OR Antrim:ti,ab OR Aontroim:ti,ab OR 'Contae Aontroma':ti,ab OR Anthrim:ti,ab OR Antrìm:ti,ab OR Entrim:ti,ab OR Armagh:ti,ab OR 'Ard Mhacha':ti,ab OR Airmagh:ti,ab OR Belfast:ti,ab OR (Down:ti,ab AND (district:ti,ab OR council:ti,ab OR County:ti,ab)) OR 'An Dún':ti,ab OR 'an Dúin':ti,ab OR Doon:ti,ab OR Doun:ti,ab OR Fermanagh:ti,ab OR 'Fear Manach':ti,ab OR 'Fhear Manach':ti,ab OR Fermanay:ti,ab OR Londonderry:ti,ab OR Doire:ti,ab OR Dhoire:ti,ab OR Lunnonderrie:ti,ab OR Derry:ti,ab OR Birmingham:ti,ab OR Leeds:ti,ab OR Sheffield:ti,ab OR Bradford:ti,ab OR Liverpool:ti,ab OR 'Iceland'/exp OR 'Icelander'/exp OR Iceland:ti,ab OR Icelandic*:ti,ab OR islenska*:ti,ab OR Icelander*:ti,ab OR islendinga*:ti,ab OR Islendigar:ti,ab OR Inslenska:ti,ab OR Reykjavík:ti,ab OR Reykjavíkurborg:ti,ab OR Hofudborgarsvaedi:ti,ab OR Sudurnes:ti,ab OR Vesturland:ti,ab OR Vestfirdir:ti,ab OR Westfjords:ti,ab OR Nordurland:ti,ab OR Austurland:ti,ab OR Sudurland:ti,ab OR Kopavogur:ti,ab OR Hafnarfjordur:ti,ab OR Akureyri:ti,ab OR Gardabaer:ti,ab OR Mosfellsbaer:ti,ab OR Keflavik:ti,ab OR Akranes:ti,ab OR Selfoss:ti,ab OR Seltjarnarnes:ti,ab OR 'Bosnia and Herzegovina'/exp OR 'Bosnian (citizen)'/exp OR 'Bosniak (people)'/exp OR Bosnia*:ti,ab OR Herzegov*:ti,ab OR Herzegonine:ti,ab OR Bosna:ti,ab OR Bosne:ti,ab OR Bosanski:ti,ab OR Bosanac:ti,ab OR Bosanci:ti,ab OR Srpska:ti,ab OR Brcko:ti,ab OR Posavski:ti,ab OR Posavina:ti,ab OR posavska:ti,ab OR Tuzlanski:ti,ab OR Tuzla:ti,ab OR Tuzlanska:ti,ab OR 'Zenickho dobojski':ti,ab OR 'Zenicko dobojska':ti,ab OR Zenica:ti,ab OR 'Bosansko Podrinjski':ti,ab OR 'Bosansko Podrinjska':ti,ab OR Srednjobosanski:ti,ab OR hercegovacko:ti,ab OR Zapadnohercegovacki:ti,ab OR Zapadnohercegovacka:ti,ab OR Sarajevo:ti,ab OR Sarajevska:ti,ab OR 'Kanton 10':ti,ab OR '10 kanton':ti,ab OR Hercegbosanska:ti,ab OR 'Unsko sanski':ti,ab OR 'Una Sana':ti,ab OR 'Banja Luka':ti,ab OR bijeljina:ti,ab OR Mostar:ti,ab OR Prijedor:ti,ab OR Cazin:ti,ab OR Doboj:ti,ab OR Zupanija:ti,ab OR 'Kosovo'/exp OR 'Kosovar'/exp OR Kosov*:ti,ab OR Ferizaj*:ti,ab OR Urosevac*:ti,ab OR Gjakov*:ti,ab OR Dakovic*:ti,ab OR Gjilan*:ti,ab OR Gnjilan*:ti,ab OR Mitrovic*:ti,ab OR Pejes:ti,ab OR Peja:ti,ab OR Peje:ti,ab OR Pecki:ti,ab OR Pec:ti,ab OR Pristin*:ti,ab OR Prishtin*:ti,ab OR Pristinski:ti,ab OR Prizrenit:ti,ab OR Prizrenski:ti,ab OR Prizen:ti,ab OR Prizren:ti,ab OR Prizeni:ti,ab OR Produjev*:ti,ab OR Vucitrn:ti,ab OR Vushtrri*:ti,ab OR 'Suva reka':ti,ab OR Suhareka:ti,ab OR Besiana:ti,ab OR Metohija:ti,ab OR Dukagjini:ti,ab OR Dukagjinit:ti,ab OR 'Liechtenstein'/exp OR Liechtenstein:ti,ab OR Lienchtensteiner*:ti,ab OR Balzers:ti,ab OR Eschen:ti,ab OR Gamprin:ti,ab OR Mauren:ti,ab OR Planken:ti,ab OR Ruggell:ti,ab OR Schaan:ti,ab OR Schellenberg:ti,ab OR Triesen:ti,ab OR Triesenberg:ti,ab OR Vaduz:ti,ab OR 'Norway'/exp OR 'Norwegian (citizen)'/exp OR 'Norwegian (people)'/exp OR Norway:ti,ab OR Norwegian*:ti,ab OR Norge:ti,ab OR Noreg:ti,ab OR Norgga:ti,ab OR Akershus:ti,ab OR 'Aust Agder':ti,ab OR Buskerud:ti,ab OR Finnmark:ti,ab OR Hedmark:ti,ab OR Hordaland:ti,ab OR 'More og Romsdal':ti,ab OR 'More and Romsdal':ti,ab OR 'More Romsdal':ti,ab OR Nordland:ti,ab OR Trondelag:ti,ab OR Oppland:ti,ab OR Oslo:ti,ab OR Ostfold:ti,ab OR Rogaland:ti,ab OR 'Sogn og fjordane':ti,ab OR 'Sogn and fjordane':ti,ab OR 'sogn fjordane':ti,ab OR Telemark:ti,ab OR Troms:ti,ab OR Romsa:ti,ab OR Romssa:ti,ab OR 'Vest Agder':ti,ab OR Vestfold:ti,ab OR Bergen:ti,ab OR Stavanger:ti,ab OR Sandnes:ti,ab OR Trondheim:ti,ab OR Trondhjem:ti,ab OR Kaupangen:ti,ab OR Nidaros:ti,ab OR Drammen:ti,ab OR Fredrikstad:ti,ab OR Skien:ti,ab OR Tromso:ti,ab OR Sarpsborg:ti,ab OR europe*:ad OR europa*:ad OR eu:ad OR eea:ad OR efta:ad OR 'eu/eea':ad OR 'eu/efta':ad OR ecsc:ad OR euratom:ad OR eurozone:ad OR eec:ad OR ec:ad OR (schengen:ad AND (area:ad OR countr*:ad OR region*:ad OR state:ad OR states:ad)) OR euroregion:ad OR euroregions:ad OR balkan:ad OR balkans:ad OR baltic:ad OR (mediterranean:ad AND (area:ad OR countr*:ad OR region*:ad OR state:ad OR states:ad)) OR (alpine:ad AND (area:ad OR countr*:ad OR region*:ad OR state:ad OR states:ad)) OR scandinavia:ad OR scandinavian:ad OR (nordic NEXT/1 (countr* OR state*)):ad OR danubian:ad OR 'iberian peninsula':ad OR 'peninsula iberica':ad OR 'péninsule ibérique':ad OR 'iberiar penintsula':ad OR iberia:ad OR anatolia:ad OR anadolu:ad OR anatole:ad OR anatolian:ad OR yugoslavia:ad OR czechoslovakia:ad OR 'czecho slovakia':ad OR ceskoslovensko:ad OR 'cesko slovensko':ad OR benelux:ad OR fennoscandia:ad OR 'fenno scandinavia':ad OR fennoskandi*:ad OR (visegrád:ad AND (group:ad OR four:ad OR triangle:ad)) OR 'visegrádská čtyřka':ad OR 'visegrádská skupina':ad OR 'visegrádi együttműködés':ad OR 'visegrádi négyek':ad OR 'grupa wyszehradzka':ad OR 'vyšehradská skupina':ad OR 'vyšehradská štvorka':ad OR austria*:ad OR osterreich*:ad OR oesterreich*:ad OR ostosterreich:ad OR ostoesterreich:ad OR sudosterreich:ad OR sudoesterreich:ad OR westosterreich:ad OR westoesterreich:ad OR burgenland:ad OR carinthia:ad OR karnten:ad OR kaernten:ad OR niederosterreich:ad OR niederoesterreich:ad OR oberosterreich:ad OR oberoesterreich:ad OR salzburg:ad OR saizburg:ad OR styria:ad OR steiermark:ad OR tyrol:ad OR tirol:ad OR vorarlberg:ad OR vienna:ad OR wien:ad OR graz:ad OR linz:ad OR innsbruck:ad OR klagenfurt:ad OR villach:ad OR wels:ad OR 'st polten':ad OR 'st poelten':ad OR 'sankt polten':ad OR 'sankt poelten':ad OR dornbirn:ad OR Belgi*:ad OR Belge*:ad OR Belg:ad OR Brussel*:ad OR Bruxelles:ad OR Bruxelloise:ad OR Walloon*:ad OR Wallon*:ad OR Vlaams:ad OR Flander*:ad OR Flandern:ad OR Flandre:ad OR Flemish:ad OR Flamand:ad OR Flemisch:ad OR Flämisch*:ad OR Vlaanderen:ad OR Flamande:ad OR Waals:ad OR Antwerp*:ad OR Anvers:ad OR Henegouwen:ad OR Hennegau:ad OR Hainault:ad OR Hainaut:ad OR Liege:ad OR Luik:ad OR Luttich:ad OR Limbourg:ad OR Limburg:ad OR Namur:ad OR Namen:ad OR Ostflandern:ad OR Westflandern:ad OR Ghent:ad OR Gent:ad OR Gand:ad OR Charleroi:ad OR Bruges:ad OR Brugge*:ad OR Schaerbeek:ad OR Schaarbeek:ad OR Anderlecht:ad OR Leuven:ad OR Louvain:ad OR Bulgaria:ad OR balgariya:ad OR balgarija:ad OR blagoevgrad*:ad OR 'pirin macedonia':ad OR burgas:ad OR dobrich:ad OR gabrovo:ad OR haskovo:ad OR kardzhali:ad OR kurdzhali:ad OR kyustendil:ad OR lovech:ad OR lovec:ad OR montana:ad OR pazardzhik:ad OR pernik:ad OR pleven*:ad OR plovdiv:ad OR razgrad:ad OR rousse:ad OR ruse:ad OR rusenka:ad OR shumen:ad OR silistra:ad OR sliven:ad OR smolyan:ad OR sofia:ad OR sofyiska:ad OR sofiiska:ad OR 'stara zagora':ad OR targovishte:ad OR varna:ad OR 'veliko tarnovo':ad OR vidin:ad OR vratsa:ad OR vratza:ad OR yambol:ad OR croat*:ad OR hrvatsk*:ad OR hrvati:ad OR bjelovar:ad OR 'bjelovarsko bilogorska':ad OR 'brod posavina':ad OR 'brodsko posavska':ad OR 'dubrovnik neretva':ad OR 'dubrovacko neretvanska':ad OR zagreb:ad OR zagrebacka:ad OR istria:ad OR istarska:ad OR karlovacka:ad OR karlovac:ad OR 'koprivnicko krizevacka':ad OR koprivnica:ad OR krizevci:ad OR 'krapina zagorje':ad OR 'krapinsko zagorska':ad OR 'lika senj':ad OR 'licko senjska':ad OR medimurska:ad OR medimurje:ad OR osijek:ad OR baranja:ad OR 'osjecko baranjska':ad OR 'pozega slavonia':ad OR 'pozesko slavonska':ad OR 'primorje gorski kotar':ad OR 'primorsko goranska':ad OR 'sibensko kninska':ad OR 'sibensko kninske':ad OR sibenik:ad OR knin:ad OR sisak:ad OR 'sisacko moslavacka':ad OR moslavina:ad OR 'splitsko dalmatinska':ad OR split:ad OR dalmatia:ad OR varazdin:ad OR varazdinska:ad OR viroviticko:ad OR podravska:ad OR virovitica:ad OR podravina:ad OR 'vukovarsko srijemska':ad OR vukovar:ad OR srijem:ad OR zadar:ad OR zadarska:ad OR rijeka:ad OR 'velika gorica':ad OR 'slavonski brod':ad OR pula:ad OR cyprus:ad OR cypriot*:ad OR kypros:ad OR kibris*:ad OR kypriaki:ad OR kyprioi:ad OR nicosia:ad OR lefkosa:ad OR lefkosia:ad OR famagusta:ad OR magusa:ad OR ammochostos:ad OR gazimagusa:ad OR kyrenia:ad OR girne:ad OR keryneia:ad OR larnaca:ad OR larnaka:ad OR iskele:ad OR limassol:ad OR lemesos:ad OR limasol:ad OR leymosun:ad OR paphos:ad OR pafos:ad OR baf:ad OR strovolos:ad OR lakatamia:ad OR lakadamya:ad OR 'kato polemidia':ad OR 'kato polemidhia':ad OR aglandjia:ad OR eglence:ad OR aglantzia:ad OR aradhippou:ad OR aradippou:ad OR engomi:ad OR czech*:ad OR cesky:ad OR ceska:ad OR cech:ad OR cestina:ad OR prague:ad OR praha:ad OR prag:ad OR stredoces*:ad OR jihoces*:ad OR bohemia:ad OR bohemian:ad OR plzen*:ad OR pilsen:ad OR karlovars*:ad OR 'karlovy vary':ad OR usteck*:ad OR usti:ad OR liberec*:ad OR 'hradec kralove':ad OR kralovehradec*:ad OR pardubic*:ad OR olomouc*:ad OR olomoc:ad OR holomoc:ad OR moravskoslezs*:ad OR jihomorav*:ad OR moravia:ad OR moravian:ad OR morava:ad OR vysocina:ad OR zlin:ad OR zlinsk*:ad OR 'ceske budejovice':ad OR budweis:ad OR brno:ad OR ostrava:ad OR Denmark:ad OR Danish*:ad OR dane:ad OR danes:ad OR Danmark:ad OR dansk*:ad OR Hovedstaden:ad OR Midtjylland:ad OR Nordjylland:ad OR Sjaelland:ad OR Sealand:ad OR 'Zealand region':ad OR 'region Zealand':ad OR Syddanmark:ad OR Jutland:ad OR Jylland:ad OR Sonderjyllands:ad OR Copenhagen:ad OR Kobenhavn:ad OR Arhus:ad OR Aarhus:ad OR Bornholm:ad OR Frederiksberg:ad OR Frederiksborg:ad OR Ringkjobing:ad OR Viborg:ad OR Vejle:ad OR Roskilde:ad OR Storstrøm:ad OR Vestsjaellands:ad OR 'West Zealand':ad OR Funen:ad OR Ribe:ad OR 'Kalaallit Nunaat':ad OR Gronland:ad OR Foroyar:ad OR Faeroerne:ad OR 'Faroe islands':ad OR Aalborg:ad OR Alborg:ad OR Odense:ad OR Esbjerg:ad OR Gentofte:ad OR Gladsaxe:ad OR Randers:ad OR Kolding:ad OR Estonia*:ad OR Eesti:ad OR Eestlased:ad OR Eestlane:ad OR Harju:ad OR Harjumaa:ad OR Hiiu:ad OR Hiiumaa:ad OR 'Ida Viru':ad OR 'Ida Virumaa':ad OR Jarvamaa:ad OR Jarva:ad OR Jogevamaa:ad OR Jogeva:ad OR Laanemaa:ad OR Laane:ad OR 'Laane Virumaa':ad OR Parnu:ad OR Parnumaa:ad OR Polva:ad OR Polvamaa:ad OR Rapla:ad OR Raplamaa:ad OR Saare:ad OR Saaremaa:ad OR Tartu:ad OR Tartumaa:ad OR Valga:ad OR Valgamaa:ad OR Viljandimaa:ad OR Viljandi:ad OR Voru:ad OR Vorumaa:ad OR Tallinn:ad OR Narva:ad OR 'Kohtla Jarve':ad OR Rakvere:ad OR Maardu:ad OR Sillamae:ad OR Kuressaare:ad OR Finland:ad OR Finnish*:ad OR Finn:ad OR Finns:ad OR Suomi:ad Suomen:ad OR Suomalaiset:ad OR Aland:ad OR Ahvenanmaa:ad OR Uusimaa:ad OR Nyland:ad OR Karelia:ad OR Karjala:ad OR Karelen:ad OR Ostrobothnia:ad OR Pohjanmaa:ad OR Osterbotten:ad OR Savonia:ad OR Savo:ad OR Savolax:ad OR Kainuu:ad OR Kajanaland*:ad OR 'Kanta Hame':ad OR Tavastia:ad OR Tavastland:ad OR Kymenlaakso:ad OR Kymmenedalen:ad OR Lapland:ad OR Lappi:ad OR Lappland:ad OR 'Paijat Hame':ad OR Pirkanmaa:ad OR Birkaland:ad OR Satakunta:ad OR Satakunda:ad OR Helsinki:ad OR Helsingfors:ad OR Espoo:ad OR Esbo:ad OR Tampere:ad OR Tammerfors:ad OR Vantaa:ad OR Vanda:ad OR Oulu:ad OR Uleaborg:ad OR Turku:ad OR Abo:ad OR Jyvaskyla:ad OR Kuopio:ad OR Lahti:ad OR Lahtis:ad OR Kouvola:ad OR France:ad OR French*:ad OR Francais*:ad OR Alsace:ad OR Elsass:ad OR Aquitaine:ad OR Aquitania:ad OR Akitania:ad OR Aguiéne:ad OR Auvergne:ad OR Auvèrnhe:ad OR Auvèrnha:ad OR Normandie:ad OR Normandy:ad OR Normaundie:ad OR Bourgogne:ad OR Burgundy:ad OR Bregogne:ad OR Borgoégne:ad OR Borgogne:ad OR Brittany:ad OR Breizh:ad OR Bertaèyn:ad OR Bretagne:ad OR 'Champagne Ardenne':ad OR Corse:ad OR Corsica:ad OR 'Franche Comte':ad OR 'Frantche Comte':ad OR 'Franche Comtat':ad OR Guadeloupe:ad OR Guyane:ad OR Guiana:ad OR 'Languedoc Roussillon':ad OR 'Lengadoc Rosselhon':ad OR 'Llenguadoc-Rossello':ad OR Limousin:ad OR Lemosin:ad OR Lorraine:ad OR Lothringen:ad OR Lottringe:ad OR Martinique:ad OR 'Midi Pyrenees':ad OR 'Miègjorn Pirenèus':ad OR 'Mieidia Pirenèus':ad OR 'Mediodia Pirineos':ad OR 'Pays de la Loire':ad OR 'Broioù al Liger':ad OR Picardie:ad OR Picardy:ad OR 'Poitou Charentes':ad OR 'Peitau Charantas':ad OR 'Poetou-Cherentes':ad OR Provence:ad OR Provenca:ad OR Prouvenco:ad OR 'Cote d Azur':ad OR 'Costo d Azur':ad OR 'Costa d Azur':ad OR Reunion:ad OR 'Rhone Alpes':ad OR 'Rono Arpes':ad OR 'Rose Aups':ad OR Ain:ad OR Aisne:ad OR Allier:ad OR 'Alpes de Haute Provence':ad OR 'Haute Alpes':ad OR 'Alpes Maritimes':ad OR Ardeche:ad OR Ardennes:ad OR Ariege:ad OR Aube:ad OR Aude:ad OR Aveyron:ad OR 'Bas Rhin':ad OR 'Bouches du Rhone':ad OR Calvados:ad OR Cantal:ad OR Charente:ad OR Cher:ad OR Correze:ad OR 'Corse du Sud':ad OR 'Cote d Or':ad OR 'Cotes d Armor':ad OR Creuse:ad OR 'Deux Sevres':ad OR Dordogne:ad OR Doubs:ad OR Drome:ad OR Essonne:ad OR Eure:ad OR Finistere:ad OR Gard:ad OR Gers:ad OR Gironde:ad OR 'Haute Corse':ad OR 'Haute Garonne':ad OR 'Haute Marne':ad OR 'Hautes Alpes':ad OR 'Haute Saone':ad OR 'Haute Savoie':ad OR 'Hautes Pyrenees':ad OR 'Haute Vienne':ad OR 'Haut Rhin':ad OR 'Hauts de Seine':ad OR Herault:ad OR 'Ile de France':ad OR 'Ille et Vilaine':ad OR Indre:ad OR Isere:ad OR Jura:ad OR Landes:ad OR Loire:ad OR Loiret:ad OR (Lot NEAR/3 (departement OR department)):ad OR 'Lot et Garonne':ad OR 'Loir et Cher':ad OR Lozere:ad OR Manche:ad OR Marne:ad OR Mayenne:ad OR Mayotte:ad OR 'Meurthe et Moselle':ad OR Meuse:ad OR Morbihan:ad OR Moselle:ad OR (Nord NEAR/3 (department OR departement)):ad OR Nievre:ad OR Oise:ad OR Orne:ad OR 'Pas de calais':ad OR 'Noord-Nauw van Kales':ad OR Paris:ad OR 'Puy de dome':ad OR 'Pyrenees Atlantiques':ad OR 'Pyrenees Orientales':ad OR Rhone:ad OR Sarthe:ad OR Savoie:ad OR 'Seine et Marne':ad OR 'Seine Maritime':ad OR Somme:ad OR Tarn:ad OR 'Territoire de Belfort':ad OR 'Val de Marne':ad OR 'Val d Oise':ad OR Var:ad OR Vaucluse:ad OR Vendee:ad OR Vienne:ad OR Vosges:ad OR Yonne:ad OR Yvelines:ad OR Marseille:ad OR Lyon:ad OR Nice:ad OR Nantes:ad OR Strasbourg:ad OR Montpellier:ad OR Bordeaux:ad OR Lille:ad OR Toulouse:ad OR 'Outre Mer':ad OR 'Seine Saint Denis':ad OR German*:ad OR Deutsch*:ad OR Bundesrepublik:ad OR Westdeutschland:ad OR Ostdeutschland:ad OR Baden:ad OR Wuerttemberg:ad OR Wurttemberg:ad OR Bayern:ad OR Bavaria:ad OR Berlin:ad OR Brandenburg:ad OR Bremen:ad OR Oldenburg:ad OR Mitteldeutschland:ad OR Rhein:ad OR Rhine:ad OR Hannover:ad OR Braunschweig:ad OR Göttingen:ad OR Goettingen:ad OR Nurnberg:ad OR Nuernberg:ad OR Ruhr:ad OR Koln:ad OR koeln:ad OR Bonn:ad OR Hamburg:ad OR Hessen:ad OR Hesse:ad OR Hessia:ad OR Mecklenburg:ad OR Vorpommern:ad OR Pomerania:ad OR Niedersachsen:ad OR Neddersassen:ad OR Saxony:ad OR Niederbayern:ad OR 'Northern Rhine':ad OR 'North Rhine':ad OR Westphalia:ad OR Westfalen:ad OR 'Rhineland Palatinate':ad OR 'Rheinland Pfalz':ad OR Saarland:ad OR Sachsen:ad OR 'Schleswig Holstein':ad OR Thuringia:ad OR Thuringen:ad OR Thueringen:ad OR Munchen:ad OR Muenchen:ad OR Munich:ad OR Frankfurt:ad OR Stuttgart:ad OR Dusseldorf:ad OR Duesseldorf:ad OR Dortmund:ad OR Essen:ad OR Greece:ad OR 'Hellenic republic':ad OR Greek*:ad OR Ellada:ad OR Elladas:ad OR 'Elliniki Dimokratia':ad OR Hellas:ad OR Hellenes:ad OR Attica:ad OR Attiki:ad OR Makedonia*:ad OR Macedonia:ad OR Thraki:ad OR Thrace:ad OR Crete:ad OR Kriti:ad OR 'Ionia Nisia':ad OR 'Ionion neson':ad OR 'Ionion nIson':ad OR 'Ionian islands':ad OR 'Ionian island':ad OR Epirus:ad OR Ipeiros:ad OR 'Periféreia Ipeírou':ad OR 'North aegean':ad OR 'Northern Aegean':ad OR 'Aegean islands':ad OR 'Aegean island':ad OR 'Nisoi Agaiou':ad OR 'Notio Aigaio':ad OR Peloponnese:ad OR Peloponniso*:ad OR Thessaly:ad OR Thessalia:ad OR Thessalian:ad OR Petthalia:ad OR 'Voreio Aigaio':ad OR 'Voreio Aigaiou':ad OR 'South aegean':ad OR 'Southern Aegean':ad OR 'Mount athos':ad OR 'Oros Athos':ad OR Cyclades:ad OR Cycklades:ad OR Kiklades:ad OR Dodecanese:ad OR Dodekanisa:ad OR Athens:ad OR Athina:ad OR Thessaloniki:ad OR Thessalonica:ad OR Patras:ad OR Patra:ad OR Pireas:ad OR Piraeus:ad OR Larissa:ad OR Larisa:ad OR Heraklion:ad OR Heraclion:ad OR Iraklion:ad OR Irakleion:ad OR Iraklio:ad OR Volos:ad OR Rhodes:ad OR Rodos:ad OR Ioannina:ad OR Janina:ad OR Yannena:ad OR Chania:ad OR Chalcis:ad OR Chalkida:ad OR Hungar*:ad OR Magyarorszag:ad OR Magyar*:ad OR Dunantuli:ad OR Transdanubia:ad OR Dunantul:ad OR 'Great Plain':ad OR 'Eszak Alfold':ad OR 'Del Alfold':ad OR 'Alfold es eszak':ad OR 'Northern Alfold':ad OR 'North Alfold':ad OR 'South Alfold':ad OR 'Southern Alfold':ad OR Bacs:ad OR Kiskun:ad OR Baranya:ad OR Bekes:ad OR Borsod:ad OR Abauj:ad OR Zemplen:ad OR Budapest:ad OR Csongrad:ad OR Fejer:ad OR gyor:ad OR moson:ad OR sopron:ad OR hajdu:ad OR bihar:ad OR Heves:ad OR 'jasz nagykun szolnok':ad OR komarom:ad OR esztergom:ad OR Nograd:ad OR (Pest NEXT/3 (megye OR county)):ad OR Somogy:ad OR szabolcs:ad OR szatmar:ad OR bereg:ad OR Tolna:ad OR Vas:ad OR Veszprem:ad OR Zala:ad OR Debrecen:ad OR Miskolc:ad OR Szeged:ad OR Pecs:ad OR Gyor:ad OR Nyiregyhaza:ad OR Kecskemet:ad OR Szekesfehervar:ad OR Szombathely:ad OR Ireland:ad OR Eire:ad OR Irish*:ad OR Fingal:ad OR 'Fine Gall':ad OR Dublin:ad OR 'Ath Cliath':ad OR 'Dun Laoghaire':ad OR Wicklow:ad OR 'Cill Mhantain':ad OR 'Chill Mhantain':ad OR Wexford:ad OR 'Loch Garman':ad OR Carlow:ad OR Ceatharlach:ad OR Kildare:ad OR 'Cill Dara':ad OR 'Chill Dara':ad OR Meath:ad OR 'An Mhi':ad OR 'Contae na Mi':ad OR Louth:ad OR 'Contae Lu':ad OR Monaghan:ad OR Muineachán:ad OR Mhuineacháin:ad OR Cavan:ad OR 'An Cabhan':ad OR 'An Cabhain':ad OR Longford:ad OR 'An Longfort':ad OR 'an Longfoirt':ad OR Langfurd:ad OR Westmeath:ad OR 'An Iarmhi':ad OR 'na Iarmhi':ad OR Offaly:ad OR 'Uibh Fhaili':ad OR Laois:ad OR Laoise:ad OR Kilkenny:ad OR 'Chill Chainnigh':ad OR 'Cill Chainnigh':ad OR Waterford:ad OR 'Port Lairge':ad OR Watterford:ad OR Cork:ad OR Corcaigh:ad OR Chorcai:ad OR Kerry:ad OR Ciarrai:ad OR Chiarrai:ad OR Limerick:ad OR Luimneach:ad OR Luimnigh:ad OR Tipperary:ad OR 'Tiobraid Arann':ad OR 'Thiobraid Arann':ad OR Clare:ad OR 'An Clar':ad OR 'an Chlair':ad OR Galway:ad OR Gaillimh:ad OR 'na Gaillimhe':ad OR Mayo:ad OR 'Maigh Eo':ad OR 'Mhaigh Eo':ad OR Roscommon:ad OR 'Ros comain':ad OR Sligo:ad OR Sligeach:ad OR Shligigh:ad OR Leitrim:ad OR Liatroim:ad OR Liatroma:ad OR Donegal:ad OR 'Dhún na nGall':ad OR Dinnygal:ad OR Dunnyga:ad OR Leinster:ad OR Laighin:ad OR 'Cúige Laighean':ad OR Munster:ad OR Mumhain:ad OR 'Cúige Mumhan':ad OR Connacht:ad OR Connachta:ad OR Drogheda:ad OR 'Droichead Atha':ad OR Dundalk:ad OR 'Dún Dealgan':ad OR Swords:ad OR Sord:ad OR Bray:ad OR Bre:ad OR Navan:ad OR 'An Uaimh':ad OR Italy:ad OR Italia*:ad OR Abruzzo:ad OR Abruzzi:ad OR Basilicata:ad OR Lucania:ad OR Calabria:ad OR Campania:ad OR 'Emilia Romagna':ad OR 'friuli venezia giulia':ad OR Lazio:ad OR Latium:ad OR Liguria*:ad OR Lombardy:ad OR Lombardia:ad OR Marche:ad OR Marches:ad OR Molisano:ad OR Molise:ad OR Piedmont*:ad OR Piemonte:ad OR Bolzano:ad OR Bozen:ad OR Trentino:ad OR Trento:ad OR Puglia:ad OR Apulia:ad OR Sardinia:ad OR Sardegna:ad OR Sicily:ad OR Sicilia:ad OR Toscana:ad OR Tuscany:ad OR Umbria:ad OR 'Valle d Aosta':ad OR 'Vallee d Aoste':ad OR 'Aosta Valley':ad OR Veneto:ad OR Venetia:ad OR Triveneto:ad OR Rome:ad OR Roma:ad OR Milan:ad OR Milano:ad OR Naples:ad OR Napoli:ad OR Turin:ad OR Torino:ad OR Palermo:ad OR Genoa:ad OR Genova:ad OR Bologna:ad OR Florence:ad OR Firenze:ad OR Bari:ad OR Catania:ad OR Latvi*:ad OR Riga:ad OR Courland:ad OR Kurzeme:ad OR Kurland:ad OR Latgale:ad OR Lettgallia:ad OR Latgola:ad OR Latgalia:ad OR Vidzeme:ad OR Vidumo:ad OR Semigallia:ad OR Semigalia:ad OR Zemgale:ad OR Pieriga:ad OR Daugavpils:ad OR Dinaburg:ad OR Jekabpils:ad OR Jakobstadt:ad OR Jelgava:ad OR Jurmala:ad OR Liepaja:ad OR Libau:ad OR Rezekne:ad OR Rezne:ad OR Rositten:ad OR Valmiera:ad OR Wolmar:ad OR Ventspils:ad OR Windau:ad OR Ogre:ad OR Lithuania*:ad OR 'Lietuvos Respublika':ad OR Lietuva:ad OR lietuviu:ad OR Alytus:ad OR Alytaus:ad OR Kaunas:ad OR Kauno:ad OR Klaipeda:ad OR Klaipedos:ad OR Marijampoles:ad OR Marijampole:ad OR Panevezys:ad OR Panevezio:ad OR Siauliai:ad OR Siauliu:ad OR Taurages:ad OR Taurage:ad OR Telsiu:ad OR Telsiai:ad OR Utenos:ad OR Utena:ad OR Vilnius:ad OR Vilniaus:ad OR Mazeikiai:ad OR Jonava:ad OR Mazeikiu:ad OR Jonavos:ad OR Luxembourg*:ad OR Luxemburg:ad OR Letzebuerg:ad OR Diekirch:ad OR Grevenmacher:ad OR 'Esch sur Alzette':ad OR 'Esch Uelzecht':ad OR 'Esch an der Alzette':ad OR 'Esch an der Alzig':ad OR Dudelange:ad OR Diddeleng:ad OR Düdelingen:ad OR Duedelingen:ad OR Schifflange:ad OR Scheffleng:ad OR Schifflingen:ad OR Bettembourg:ad OR Beetebuerg:ad OR Bettemburg:ad OR Petange:ad OR Peiteng:ad OR Petingen:ad OR Ettelbruck:ad OR Ettelbreck:ad OR Ettelbrueck:ad OR Diekirch:ad OR Dikrech:ad OR Strassen:ad OR Stroossen:ad OR Bertrange:ad OR Bartreng:ad OR Bartringen:ad OR Malta:ad OR Maltese*:ad OR Maltin:ad OR Gozo:ad OR Ghawdex:ad OR Valletta:ad OR 'Ill Belt':ad OR Birkirkara:ad OR 'B Kara':ad OR Birchircara:ad OR Mosta:ad OR Qormi:ad OR 'St Paul s Bay':ad OR 'Pawl il Bahar':ad OR Zabbar:ad OR Sliema:ad OR Naxxar:ad OR Gwann:ad OR 'St John':ad OR Zebbug:ad OR 'Citta rohan':ad OR Fgura:ad OR Netherlands:ad OR Nederland*:ad OR Dutch*:ad OR Drenthe:ad OR Flevoland:ad OR Friesland:ad OR Fryslan:ad OR Frisia:ad OR Gelderland:ad OR Guelders:ad OR Groningen:ad OR Limburg:ad OR Brabant:ad OR Holland:ad OR Overijssel:ad OR Overissel:ad OR Utrecht:ad OR Zeeland:ad OR Amsterdam:ad OR Rotterdam:ad OR Hague:ad OR 's-Gravenhage':ad OR 'Den Haag':ad OR Eindhoven:ad OR Tilburg:ad OR Almere:ad OR Breda:ad OR Nijmegen:ad OR Nimeguen:ad OR Poland:ad OR Polska:ad OR Polish:ad OR Pole:ad OR Poles:ad OR Polski:ad OR Polak:ad OR Polka:ad OR Polacy:ad OR Dolnoslaskie:ad OR Silesia*:ad OR Slask:ad OR Pomorskie:ad OR Pomerania*:ad OR Kujawsko:ad OR Kuyavian:ad OR Lodzkie:ad OR Lodz:ad OR Lubelskie:ad OR Lublin:ad OR Lubuskie:ad OR Lubusz:ad OR Lubus:ad OR Malopolskie:ad OR Mazowieckie:ad OR Mazowske:ad OR Masovia:ad OR Masovian:ad OR Opolskie:ad OR Opole:ad OR Podkarpackie:ad OR Subcarpathian*:ad OR Podlaskie:ad OR Podlachia:ad OR Podlasie:ad OR Slaskie:ad OR Swietokrzyskie:ad OR 'Varmia Mazuria':ad OR 'Varmian Mazurian':ad OR 'Varmia Masuria':ad OR 'Varmian Masurian':ad OR 'Warmia Mazury':ad OR 'Warminsko Mazurskie':ad OR 'Warmian Masurian':ad OR Wielkopolskie:ad OR Zachodniopomorskie:ad OR Warsaw:ad OR Warszawa:ad OR Krakow:ad OR Cracow:ad OR Wroclaw:ad OR Poznan:ad OR Gdansk:ad OR Szczecin:ad OR Bydgoszcz:ad OR Katowice:ad OR Portugal:ad OR Portugues*:ad OR Azores:ad OR Acores:ad OR Madeira:ad OR Alentejo:ad OR Algarve:ad OR Lisboa:ad OR Lisbon:ad OR 'Alto Tras-os-Montes':ad OR (Ave NEAR/3 (community OR intermunicipal OR comunidade)):ad OR Mondego:ad OR Vouga:ad OR Beira:ad OR Cavado:ad OR Lafoes:ad OR Douro:ad OR Porto:ad OR Oporto:ad OR Tejo:ad OR Minho:ad OR Setubal:ad OR Pinhal:ad OR 'Serra da Estrela':ad OR Tamega:ad OR Leira:ad OR Santarem:ad OR Beja:ad OR Faro:ad OR Evora:ad OR Portalegre:ad OR 'Castelo Branco':ad OR Guarda:ad OR Cimbra:ad OR Aveiro:ad OR Viseu:ad OR Braganca:ad OR Braganza:ad OR Braga:ad OR 'Vila real':ad OR 'Viana do Castelo':ad OR Gaia:ad OR Amadora:ad OR Funchal:ad OR Coimbra:ad OR Almada:ad OR (Agualva:ad AND Cacem:ad) OR Romania*:ad OR Rumania*:ad OR Roumania*:ad OR Romani:ad OR Rumani:ad OR Alba:ad OR Arad:ad OR Arges:ad OR Bacau:ad OR Bihor:ad OR 'Bistrita Nasaud':ad OR Botosani:ad OR Braila:ad OR Brasov:ad OR Kronstadt:ad OR Brasso:ad OR Brassovia:ad OR Coron:ad OR Bucharest:ad OR Bucuresti:ad OR Buzau:ad OR Calarasi:ad OR 'Caras-Severin':ad OR Cluj:ad OR Klausenburg:ad OR Kolozsvar:ad OR Constanta:ad OR Tomis:ad OR Konstantia:ad OR Kostence:ad OR Covasna:ad OR Dambovita:ad OR Dolj:ad OR Galati:ad OR Galatz:ad OR Galac:ad OR Kalas:ad OR Giurgiu:ad OR Gorj:ad OR Harghita:ad OR Hunedoara:ad OR Ialomita:ad OR Iasi:ad OR Jassy:ad OR Lassy:ad OR Ilfov:ad OR Maramures:ad OR Mehedinti:ad OR Mures:ad OR Neamt:ad OR (Olt:ad AND (river:ad OR county:ad OR region:ad OR judetul:ad OR Raul:ad)) OR Prahova:ad OR Salaj:ad OR 'Satu Mare':ad OR Sibiu:ad OR Suceava:ad OR Teleorman:ad OR Timis:ad OR Tulcea:ad OR Valcea:ad OR Vilcea:ad OR Vaslui:ad OR Vrancea:ad OR Timisoara:ad OR Temeswar:ad OR Temeschburg:ad OR Temeschwar:ad OR Temesvar:ad OR Temisvar:ad OR Timisvar:ad OR Temesva:ad OR Craiova:ad OR Ploiesti:ad OR Ploesti:ad OR Oradea:ad OR Varad:ad OR Varat:ad OR Slovakia:ad OR Slovensk*:ad OR Slovak*:ad OR Slovaci:ad OR Slovenki:ad OR Bratislav*:ad OR Presporok:ad OR Pressburg:ad OR Preßburg:ad OR Posonium:ad OR Banskobystri*:ad OR 'Banska Bystrica':ad OR Neusohl:ad OR Besztercebánya:ad OR Kosic*:ad OR Kaschau:ad OR Kassa:ad OR Nitrian*:ad OR Nitra:ad OR Neutra:ad OR Nyitra:ad OR Nyitria:ad OR Trnav*:ad OR Tyrnau:ad OR Nagyszombat:ad OR Tyrnavia:ad OR Presov*:ad OR Trencian*:ad OR Trencin:ad OR Trentschin:ad OR Trencsén:ad OR Zilina:ad OR Sillein:ad OR Zsolna:ad OR Zylina:ad OR (Martin:ad AND (city:ad OR Svaty:ad)) OR Turócszentmárton:ad OR Poprad:ad OR Deutschendorf:ad OR Zvolen:ad OR Slovenia*:ad OR Slovenija:ad OR slovensk*:ad OR Slovenci:ad OR Slovene*:ad OR Gorenjska:ad OR Carniola:ad OR Goriska:ad OR Gorizia:ad OR Jugovzhodna:ad OR Koroska:ad OR Carinthia:ad OR 'Notranjsko kraska':ad OR 'Obalno kraska':ad OR 'Coastal karst':ad OR Osrednjeslovenska:ad OR Podravska:ad OR Drava:ad OR Pomurska:ad OR Mura:ad OR Savinjska:ad OR Savinja:ad OR Spodnjeposavska:ad OR Zasavska:ad OR 'Central Sava':ad OR Posavska:ad OR 'Lower Sava':ad OR Ljubljana:ad OR Laibach:ad OR Lubiana:ad OR Maribor:ad OR 'Marburg an der Drau':ad OR Kranj:ad OR Carnium:ad OR Creina:ad OR Chreina:ad OR Krainbur:ad OR Koper:ad OR Capodistria:ad OR Kopar:ad OR Celje:ad OR 'Novo mesto':ad OR Neustadtl:ad OR Domzale:ad OR Velenje:ad OR Wollan:ad OR Woellan:ad OR 'Nova Gorica':ad OR Kamnik:ad OR Spain:ad OR Espana:ad OR Spanish:ad OR Espanol*:ad OR Spaniard*:ad OR Andalucia:ad OR Andalusia:ad OR Aragon:ad OR Arago:ad OR Cantabria:ad OR Canarias:ad OR 'Canary Islands':ad OR (Canaries:ad AND island*:ad) OR 'Castile and leon':ad OR 'Castilla y Leon':ad OR 'Castile La Mancha':ad OR 'Castilla La Mancha':ad OR Cataluna:ad OR Catalonia:ad OR Ceuta:ad OR Madrid:ad OR Melilla:ad OR Navarra:ad OR Navarre:ad OR Valencia*:ad OR Extremadura:ad OR Galicia:ad OR Balears:ad OR 'Balearic Islands':ad OR 'Balear Islands':ad OR Baleares:ad OR 'La Rioja':ad OR 'Pais Vasco':ad OR 'Basque Country':ad OR 'Baske region':ad OR Euskadi:ad OR Asturias:ad OR Murcia:ad OR Coruna:ad OR Alava:ad OR Araba:ad OR Albacete:ad OR Alicante:ad OR Alacant:ad OR Almeria:ad OR Avila:ad OR Badajoz:ad OR Badajos:ad OR Barcelona:ad OR Burgos:ad OR Caceres:ad OR Cadiz:ad OR Castellon:ad OR Castello:ad OR 'Ciudad Real':ad OR Cordoba:ad OR Cuenca:ad OR Eivissa:ad OR Ibiza:ad OR Formentera:ad OR 'El Hierro':ad OR Fuerteventura:ad OR Galiza:ad OR Girona:ad OR Gerona:ad OR 'Gran Canaria':ad OR Granada:ad OR Guadalajara:ad OR Guipuzcoa:ad OR Gipuzkoa:ad OR Huelva:ad OR Huesca:ad OR Jaen:ad OR 'La Gomera':ad OR 'La Palma':ad OR Lanzarote:ad OR Leon:ad OR Lleida:ad OR Lerida:ad OR Lugo:ad OR Malaga:ad OR Mallorca:ad OR Majorca:ad OR Menorca:ad OR Minorca:ad OR Murcia:ad OR Ourense:ad OR Orense:ad OR Palencia:ad OR Pontevedra:ad OR Salamanca:ad OR Segovia:ad OR Sevilla:ad OR Seville:ad OR Soria:ad OR Tarragona:ad OR Tenerife:ad OR Teruel:ad OR Toledo:ad OR Valladolid:ad OR Vizcaya:ad OR Biscay:ad OR Zamora:ad OR Zaragoza:ad OR Saragossa:ad OR 'Las Palmas':ad OR Bilbao:ad OR Bilbo:ad OR Sweden:ad OR Sverige:ad OR Swedish:ad OR Svenska:ad OR svenskar:ad OR Swede:ad OR Swedes:ad OR Norrland:ad OR Mellansverige:ad OR Smaland:ad OR Stockholm*:ad OR Sydsverige:ad OR Vastsverige:ad OR Blekinge:ad OR Dalarna:ad OR Gavleborg*:ad OR Gotland*:ad OR Halland*:ad OR Jamtland*:ad OR Jonkoping*:ad OR Kalmar:ad OR Kronoberg*:ad OR Norrbotten*:ad OR Orebro:ad OR Ostergotland*:ad OR Skane:ad OR Sodermanlands:ad OR Uppsala:ad OR Varmland*:ad OR Vasterbotten*:ad OR Vasternorrland*:ad OR Vastmanland*:ad OR vastergotland*:ad OR Gotaland*:ad OR Gothenburg:ad OR Goteborg:ad OR Malmo:ad OR Vasteras:ad OR Linkoping:ad OR Helsingborg:ad OR Halsingborg:ad OR Norrkoping:ad OR 'GB':ad OR 'United kingdom':ad OR 'UK':ad OR Britain:ad OR British:ad OR England:ad OR English:ad OR Scotland:ad OR Scottish:ad OR Scots:ad OR Wales:ad OR Cymru:ad OR Welsh:ad OR 'North Ireland':ad OR 'Northern Ireland':ad OR Irish:ad OR Avon:ad OR Bedfordshire:ad OR Berkshire:ad OR Bristol:ad OR Buckinghamshire:ad OR Cambridgeshire:ad OR 'Isle of Ely':ad OR Cheshire:ad OR Cleveland:ad OR Cornwall:ad OR Cumberland:ad OR Cumbria:ad OR Derbyshire:ad OR Devon:ad OR Dorset:ad OR Durham:ad OR Essex:ad OR Gloucestershire:ad OR Hampshire:ad OR Southampton:ad OR (Hereford:ad AND Worcester:ad) OR Hertfordshire:ad OR Herefordshire:ad OR Humberside:ad OR Huntingdon:ad OR Huntingdonshire:ad OR 'Isle of Wight':ad OR Kent:ad OR Lancashire:ad OR Leicestershire:ad OR Lincolnshire:ad OR London:ad OR Manchester:ad OR Merseyside:ad OR Middlesex:ad OR Norfolk:ad OR Northamptonshire:ad OR Northumberland:ad OR Nottinghamshire:ad OR Oxfordshire:ad OR Peterborough:ad OR Rutland:ad OR Shropshire:ad OR Salop:ad OR Somerset:ad OR Yorkshire:ad OR Staffordshire:ad OR Suffolk:ad OR Surrey:ad OR Sussex:ad OR (Tyne:ad AND Wear:ad) OR Warwickshire:ad OR Midlands:ad OR Westmorland:ad OR Wiltshire:ad OR Worcestershire:ad OR 'Isle of Man':ad OR Jersey:ad OR Guernsey:ad OR 'Channel Islands':ad OR Aberdeen:ad OR Aberdeenshire:ad OR Angus:ad OR Forfarshire:ad OR Argyll:ad OR Ayrshire:ad OR Banffshire:ad OR Berwickshire:ad OR Bute:ad OR Caithness:ad OR Clackmannanshire:ad OR Cromartyshire:ad OR Dumfriesshire:ad OR Dunbartonshire:ad OR Dumbarton:ad OR Dundee:ad OR Lothian:ad OR Haddingtonshire:ad OR Edinburgh:ad OR Fife:ad OR Glasgow:ad OR Inverness-shire:ad OR Kincardineshire:ad OR Kinross-shire:ad OR Kirkcudbrightshire:ad OR Lanarkshire:ad OR Midlothian:ad OR Moray:ad OR Elginshire:ad OR Nairnshire:ad OR Orkney:ad OR Peeblesshire:ad OR Perthshire:ad OR Renfrewshire:ad OR (Ross:ad AND Cromarty:ad) OR Ross-shire:ad OR Roxburghshire:ad OR Selkirkshire:ad OR Shetland:ad OR Zetland:ad OR Stirlingshire:ad OR Sutherland:ad OR Linlithgowshire:ad OR Wigtownshire:ad OR Anglesey:ad OR Brecknockshire:ad OR Caernarfonshire:ad OR Carmarthenshire:ad OR Cardiganshire:ad OR Ceredigion:ad OR Clwyd:ad OR Denbighshire:ad OR Dyfed:ad OR Flintshire:ad OR Glamorgan:ad OR Gwent:ad OR Gwynedd:ad OR Merionethshire:ad OR Montgomeryshire:ad OR Monmouthshire:ad OR Pembrokeshire:ad OR Powys:ad OR Radnorshire:ad OR Antrim:ad OR Aontroim:ad OR 'Contae Aontroma':ad OR Anthrim:ad OR Antrìm:ad OR Entrim:ad OR Armagh:ad OR 'Ard Mhacha':ad OR Airmagh:ad OR Belfast:ad OR (Down:ad AND (district:ad OR council:ad OR County:ad)) OR 'An Dún':ad OR 'an Dúin':ad OR Doon:ad OR Doun:ad OR Fermanagh:ad OR 'Fear Manach':ad OR 'Fhear Manach':ad OR Fermanay:ad OR Londonderry:ad OR Doire:ad OR Dhoire:ad OR Lunnonderrie:ad OR Derry:ad OR Birmingham:ad OR Leeds:ad OR Sheffield:ad OR Bradford:ad OR Liverpool:ad OR Makedon*:ad OR Macedon*:ad OR Fyrom:ad OR Istocen:ad OR Severoistocen:ad OR Jugoistocen:ad OR Jugozapaden:ad OR Pelagonski:ad OR Pelagonia:ad OR Poloski:ad OR Polog:ad OR Skopski:ad OR Skopje:ad OR Ckonje:ad OR Vardar*:ad OR Bitola:ad OR Kumanovo:ad OR Prilep:ad OR Tetovo:ad OR Tetova:ad OR Tetove:ad OR Veles:ad OR Stip:ad OR Shtip:ad OR Ohrid:ad OR Gostivar:ad OR Gostivari:ad OR Strumica:ad OR Iceland:ad OR Icelandic*:ad OR islenska*:ad OR Icelander*:ad OR islendinga*:ad OR Islendigar:ad OR Inslenska:ad OR Reykjavík:ad OR Reykjavíkurborg:ad OR Hofudborgarsvaedi:ad OR Sudurnes:ad OR Vesturland:ad OR Vestfirdir:ad OR Westfjords:ad OR Nordurland:ad OR Austurland:ad OR Sudurland:ad OR Kopavogur:ad OR Hafnarfjordur:ad OR Akureyri:ad OR Gardabaer:ad OR Mosfellsbaer:ad OR Keflavik:ad OR Akranes:ad OR Selfoss:ad OR Seltjarnarnes:ad OR Bosnia*:ad OR Herzegov*:ad OR Herzegonine:ad OR Bosna:ad OR Bosne:ad OR Bosanski:ad OR Bosanac:ad OR Bosanci:ad OR Srpska:ad OR Brcko:ad OR Posavski:ad OR Posavina:ad OR posavska:ad OR Tuzlanski:ad OR Tuzla:ad OR Tuzlanska:ad OR 'Zenickho dobojski':ad OR 'Zenicko dobojska':ad OR Zenica:ad OR 'Bosansko Podrinjski':ad OR 'Bosansko Podrinjska':ad OR Srednjobosanski:ad OR hercegovacko:ad OR Zapadnohercegovacki:ad OR Zapadnohercegovacka:ad OR Sarajevo:ad OR Sarajevska:ad OR 'Kanton 10':ad OR '10 kanton':ad OR Hercegbosanska:ad OR 'Unsko sanski':ad OR 'Una Sana':ad OR 'Banja Luka':ad OR bijeljina:ad OR Mostar:ad OR Prijedor:ad OR Cazin:ad OR Doboj:ad OR Zupanija:ad OR Kosov*:ad OR Ferizaj*:ad OR Urosevac*:ad OR Gjakov*:ad OR Dakovic*:ad OR Gjilan*:ad OR Gnjilan*:ad OR Mitrovic*:ad OR Pejes:ad OR Peja:ad OR Peje:ad OR Pecki:ad OR Pec:ad OR Pristin*:ad OR Prishtin*:ad OR Pristinski:ad OR Prizrenit:ad OR Prizrenski:ad OR Prizen:ad OR Prizren:ad OR Prizeni:ad OR Produjev*:ad OR Vucitrn:ad OR Vushtrri*:ad OR 'Suva reka':ad OR Suhareka:ad OR Besiana:ad OR Metohija:ad OR Dukagjini:ad OR Dukagjinit:ad OR Liechtenstein:ad OR Lienchtensteiner*:ad OR Balzers:ad OR Eschen:ad OR Gamprin:ad OR Mauren:ad OR Planken:ad OR Ruggell:ad OR Schaan:ad OR Schellenberg:ad OR Triesen:ad OR Triesenberg:ad OR Vaduz:ad OR Norway:ad OR Norwegian*:ad OR Norge:ad OR Noreg:ad OR Norgga:ad OR Akershus:ad OR 'Aust Agder':ad OR Buskerud:ad OR Finnmark:ad OR Hedmark:ad OR Hordaland:ad OR 'More og Romsdal':ad OR 'More and Romsdal':ad OR 'More Romsdal':ad OR Nordland:ad OR Trondelag:ad OR Oppland:ad OR Oslo:ad OR Ostfold:ad OR Rogaland:ad OR 'Sogn og fjordane':ad OR 'Sogn and fjordane':ad OR 'sogn fjordane':ad OR Telemark:ad OR Troms:ad OR Romsa:ad OR Romssa:ad OR 'Vest Agder':ad OR Vestfold:ad OR Bergen:ad OR Stavanger:ad OR Sandnes:ad OR Trondheim:ad OR Trondhjem:ad OR Kaupangen:ad OR Nidaros:ad OR Drammen:ad OR Fredrikstad:ad OR Skien:ad OR Tromso:ad OR Sarpsborg:ad OR Gibraltar:ti,ab OR Gibraltar:ad OR Hebrid*:ti,ab OR Hebrid*:ad OR Svalbard*:ti,ab OR Svalbard*:ad

## Inclusion and exclusion criteria

##### Table 3. Inclusion and exclusion criteria on the topic testing

|  | **Inclusion** | **Exclusion** |
| --- | --- | --- |
| Study design/ type | - Randomised controlled trials (RCTs) - Non-randomised, prospective comparative studies - Prospective observational studies (e.g. cohort studies) - Retrospective observational studies (e.g. case-control studies) - Cross-sectional studies - Meta-analysis or systematic review (for hand search) - Conference abstracts | - Narrative review - Case reports - Non-pertinent publication types (e.g. expert opinions, letters to the editor, editorials, comments) - Animal studies - Genetic studies, biochemistry or molecular studies - Mathematical modeling studies - Studies on unlinked/anonymous testing to determine prevalence - Studies describing the sensitivity/specificity of laboratory tests |
| Country | - EU/EEA countries | - All other countries |
| Study subject | - Hepatitis B or C | - Other hepatitis |
| Study population | - General population, population subgroups possibly at risk | - Other populations |
| Specific outcomes of interest | - Description of approach - Acceptance/barriers to testing - Feasibility of testing intervention - Offer of test - Uptake and coverage of testing - Postivity rate/diagnosis rate - Changes in prevalence/incidence in population - Other outcomes relevant to assessing impact of interventions | - Outcomes not related to research questions |

##### Table 4. Reasons for exclusion of publications

| **Reasons for exclusion** | |
| --- | --- |
| Studies relevant to wider search only, without outcomes on testing initiatives or interventions | 46 |
| Country out of scope | 26 |
| Data similar to more recent article (available) | 8 |
| Narrative review/other publication type | 18 |
| No data on objectives | 66 |
| Systematic review | 14 |
| Poor quality | 1 |
| Conference proceeding before 2015 | 103 |
| Limited data available (conference proceeding) | 1 |
| Methods insufficient (conference proceeding) | 4 |
| Qualitative study (conference proceeding) | 3 |
| **Total** | **290** |

##### Table 5. Variables for data extraction

| **Variable** | **Description** | **Values** |
| --- | --- | --- |
| **Reference** | | |
| Author | Surname of first author of the article | Surname |
| Year | Year of publication of the article | Year: yyyy |
| **Study characteristics** | | |
| Country | Country for which the study report prevalence estimates | Country name |
| Period of sampling | Month/s and year/s during which study sampling was conducted | Month/s and year/s |
| Virus | Virus for which prevalence/incidence data are reported | HCV  HBV  both |
| Setting | Narrative field for relevant details of setting | Primary care  Community  Hospital  Prison  STI clinic  Migrant clinic  Other healthcare  ANC  Online  Multiple  Other |
| Level | Level at which the intervention or study is carried out | Clinic/site level  City level  Regional  National  EU/EEA wide  NR |
| Study design | Design of reported study | Randomised controlled trial  Non-randomised trial  Prospective cohort  Retrospective cohort  Pre-post study  Cross-sectional  Qualitative  Mixed methods  Surveillance/evaluation/audit  Other |
| Intervention | Does the study describe a testing intervention (e.g. novel tests, risk group testing, integrated testing and universal testing)? | Yes/No |
| Approach to improve testing | Narrative field for description of the approach used to improve HBV/HCV testing |  |
| Data collection | Method by which data on relevant outcomes was collected | Questionnaire  Clinical record database  Laboratory data  Interviews  NR  Several  Other  Medical records |
| **Study population** | | |
| Population targeted for testing | Population subgroup sampled in the study | General population  Drug users  PWID  Migrants  MSM  Prisoners  Pregnant women  Health care workers  Public safety workers  Waste workers  Sex workers  HIV+  HBV/HCV+  STI infected  People having an STI test  People engaging in high risk sexual behaviour  Sexual assault victims  Intranasal drug users  Sexual contacts of PWID  Recipients of SOHO  (Haemo)dialysis recipients  Recipients of medical/dental interventions  Diabetes patients  Transgender  Anabolic steroid users  Tattoo/piercing artists  Recipients of tattoo/piercings  Recipients of acupuncture, mesotherapy or beauty therapies  People in care homes/institutionalised people  Intellectually disabled people  Homeless  Household/family/sexual contacts  Birth cohort  Travellers  Underpriviledged people  Precarious individuals  Psychiatric patients  Refugees  People having an HVB/HVC test  People with polyarthritis  People at high risk for HBV/HCV  Students  Patients receiving Rituximab  People without healthcare coverage  Several |
| Study population and setting description | Narrative field for any further relevant information on the study population and setting |  |
| Denominator | Population at risk/that could be considered a candidate for testing | Numerical |
| **Results** | | |
| % Offer | Proportion of participants who were offered HBV/HCV testing | % |
| % Accepting | Proportion of participants who accepted HBV/HCV testing of those who were offered HBV/HCV testing | % |
| % Coverage | Proportion of participants who were tested for HBV/HCV | % |
| Positivity rate | Proportion of participants who were HBV/HCV positive of those who were tested for HBV/HCV | % |
| % Newly diagnosed | Proportion of participants who were newly diagnosed with HBV/HCV of those who were tested for HBV/HCV | % |
| Feasibility and acceptability | Narrative field for relevant outcomes regarding the feasibility and acceptability of the testing intervention |  |
| Other HBV/HCV testing outcomes | Narrative field for other relevant outcomes related to HBV/HCV testing |  |
| **General** | | |
| Conference proceedings | A paper presented at a conference and published in a volume called a conference proceeding | Yes/No |
| Quality assessment score | Quality score assessed using SIGN checklists | ++/+/- |
| Quality assessment (studies without score) | Narrative field for aspects of quality assessment for studies for which formal assessment checklists are unavailable | Aspects in which the study diverged from the checklist (table 6) |
| General comments | Narrative field for relevant comments on the study or further interpretation of the data extraction and critical appraisal |  |

## ****Quality assessment****

##### **Table 6:** Quality assessment checklist for studies which could not be assessed by other available checklists

| - The relevance and purpose of the research are clearly described |
| --- |
| - The methods used are clearly described and appropriate for the purpose of the research |
| - The selection of the study population is adequate for the purpose of the research |
| - Data collection is adequate for the purpose of the research |
| - The theoretical background is clearly described |
| - Data are analysed in depth |
| - Results and conclusions are clearly described |
| - The study population is clearly described (including where appropriate case detection and case definition) |
| - The population is representative of the source population |
| - The denominator is chosen appropriately (e.g. in case of surveillance studies) |

## Summary tables for evidence on hepatitis testing initiatives and interventions

##### Table 7. Evidence base for the effectiveness of testing initiatives in primary health care settings

| **Reference** | **Study characteristics** | **Testing approach** | **Study population and setting** | **Sample N** | **Outcomes** | **Critical appraisal** | **General comments** |
| --- | --- | --- | --- | --- | --- | --- | --- |
| **HBV** | | | | | | | |
| Hargreaves, 2014 [1] | Country  UK  Study period  2013  Study design  Surveillance/evaluation/audit | Testing offered to all new migrant patients. Intervention included awareness-raising educational sessions for GPs and practice staff | Migrants visiting two GP practices in a migrant area of west London | 47 new migrants eligible | *Offered (%)*  100%  *Coverage (%)*  70%  *Positivity (%)*  0%  *Acceptance rate (%)*  70% | Quality score NA  No major comments | No comments |
| Roudot-Thoraval, 2015 [2] | Country  France  Study period  May 2007-May 2014 Study design  Surveillance/evaluation/audit | Coordinated programme offering testing to precarious individuals and access to care | Precarious adults attending two primary healthcare settings in Creteil | 2,223 | *Positivity (%)*  6.7%  *Newly diagnosed (%)*  6.4% | Quality score NA  No major comments | No comments |
| **HCV** | | | | | | | |
| Anderson, 2009 [3] | *Country*  UK  *Study period*  Nov 2003-Apr 2004  *Study design*  Non-randomised trial | Opportunistic, age criterion based HCV screening undertaken in one GP practice and compared with a similar practice where no intervention was undertaken | 30-54 year old patients of two general practices in Glasgow | 584 (intervention practice)  NR (control practice) | *Offer*  72% (intervention)  0% (control)  *Coverage*  20% (intervention)  0% (control)  *Positivity*  13% (intervention) | Quality score  Acceptable  No major comments | Set in a socio-economically deprived area of Glasgow with high HCV and IDU prevalence |
| Parisi, 2014 [4] | *Country*  Italy  *Study period*  Jan 2011 - Apr 2014  *Study design* Surveillance/evaluation/audit | A Prevention Program called “EASY test project” using two new oral tests to diagnose the HCV and HIV infection. | People aged >18 years, unaware of their HCV serological status and able to complete the questionnaire in Italian or English who attented two Points-of-care and one HIV-STDs public prevention outpatient clinic (“FreeDay Easy”) of the Infectious Diseases Department of San Raffaele Scientific Institute in Milan and extended to six general practitioner surgeries. | 29,600 (total) 14000 (2011) 10000 (2012) 5000 (2013) 600 (2014) | *Coverage (%)*  15.2% (total) 9.1% (2011) 14% (2012) 16.9% (2013) 22% (2014)  *Positivity (%)*  0.6% (total) 0.5% (2011) 0.5% (2011) 0.4% (2013) 4.5% (2014) | *Quality score* NA  No major comments | No comments |
| Helsper, 2010 [5] | *Country*  Netherlands  *Study period*  Campaign: Oct 2007 - Jan 2008 Before campaign: Oct 2005 - Jan 2006 & Oct 2006 - Jan 2007  *Study design*  Non-randomised trial | A public HCV campaign consisting of radio and newspaper ads and information material distributed at public places, all aiming at increasing public awareness of Hep C and stimulating those at increased risk of HCV infection to consult their GP or the regional Public Health Service for testing and, if positive, referral for treatment. In the **intervention** region, an additional support for primary care was provided by means of brochures, short courses and informative visits. | All primary care practices of GPs who were not related to shelters for drug and alcohol addicts, in two regions in the Netherlands (**Intervention:** Amersfoort region with 110 GP practices. **Control:** Apeldoorn region with 109 GP practices) | 57 (intervention 2005/2006) 172 (intervention 2007)  86 (control 2005/2006) 118 (control 2007)  Intervention proportional increase: 3.02 Control proportional increase: 1.36 OR 2.2 (95% CI 1.5-3.3) | *Positivity (%)*  0% (intervention 2005/2006) 1.7% (intervention 2007)  1.7% (control 2005/2006) 0.8% (control 2007)  Intervention increase 1.7% (95% CI -0.2% - 3.7%) Control: decrease 0.9% (95% CI -4.1% - 2.3%) Difference: 2.6% (95% CI -0.7% - 5.8%) | *Quality score* low  No major comments | No baseline characteristics table and therefore don't know if groups are comparable |
| McLeod, 2014 [6] | *Country*  UK  *Study period*  Jan 1999 - Dec 2011  *Study design*  Pre-post study | Surveillance study on HCV testing examining impact of the Hepatitis C Action Plan consisting of awareness-raising activities, for GPs and those at risk, and the introduction of DBS sampling in community drug services to overcome barriers to testing. | Data on anti-HCV tests provided by the West of Scotland Specialist Virology Centre; East of Scotland Specialist Virology Centre; Department of Medical Microbiology at Aberdeen Royal Infirmary and Ninewells Hospital & Medical School. Positive results were laboratory confirmed. | 93,954 (total) 5,421 (pre-Action Plan) 10,117 (Action Plan) | *Positivity (%)*  9% (total) 9.9% (pre-Action Plan) 7.9% (Action Plan) | *Quality score* NA  No major comments | RR also reported for initial trend, level change in number tested and change in trend over each setting |
| Lambert, 2016 [7] | *Country*  Ireland  *Study period*  NR  *Study design* Surveillance/evaluation/audit | Audit of HCV testing using the OraQuick. In addition, interviews with selected patients exploring the reasons they were lost to follow up after initial diagnosis. | A cohort of homeless people accessing the Safetynet primary healthcare services in Dublin, Ireland | 460 | *Positivity (%)*  0,26%  *Newly diagnosed (%)*  9.6% | *Quality score* NA  No major comments | No comments |
| Hargreaves, 2014 [1] | *Country*  UK  *Study period*  2013  *Study design* Surveillance/evaluation/audit | Testing offered to all new migrant patients. Intervention included awareness-raising educational sessions for GPs and practice staff | Migrants visiting two GP practices in a migrant area of west London | 47 new migrants eligible | *Offered (%)*  100%  *Coverage (%)*  70%  *Positivity (%)*  0%  *Acceptance rate (%)*  70% | *Quality score* NA  No major comments |  |
| Cullen, 2012 [8] | *Country*  UK  *Study period*  Feb 2007 –Oct 2007  *Study design*  Non-randomised trial | A targeted screening **intervention** and GP-based case finding initiative offering HCV testing to IDUs. Eligible persons were informed of the intervention, provided with information leaflets, and offered testing by their GP/practice nurse. Training was offered to practices. | Chronically HCV-infected former intravenous drug users (IDUs) who were aged 30–54 years and had indicators of past IDU (i.e. had ceased injecting at least 6 months prior to the intervention), attending eight general practices in Greater Glasgow and Clyde (GGC) NHS Board area exposed to the intervention and those attending an equivalent number of demographically comparable control practices. | 13037 practice population (intervention) 485 eligible (intervention) 422 attending (intervention)  14189 practice population (control) | *Offered (%)*  52% (range: 5%-88%) intervention  Coverage (%)  24.8% intervention 86% (of those accepting) intervention  0.3% control  *Positivity (%)*  70% intervention  40.9% (PCR positive) intervention  22% control  14% (PCR positive) control  *Newly diagnosed (%)*  71.6%  *Acceptance rate (%)*  56%-76.7%  All interviewed participants (n=23) responded positively about the acceptability, none were offended by testing offer.  Staff interviewed (n=9) viewed the intervention as an opportunity to facilitate identification, and subsequent referral | *Quality score* low  No major comments | Denominator for control group not clear.  The study focused on practises in areas of high deprivation, of which not many were willing to participate. This meant that randomisation was not feasible. |
| **Both HBV/HCV or not specified** | | | | | | | |
| Kunkel, 2015 [9] | *Country*  UK  *Study period*  NR  *Study design* Surveillance/evaluation/audit | A pilot study (HEPScreen Project) of general practitioner (GP) based testing for viral hepatitis in East London. Patients were invited to attend practice for testing. Half of the patients were additionally invited for HIV testing to investigate the influence on uptake. | Registers of two GP practices in East London were screened for migrants at risk of viral hepatitis. African, South Asian and Turkish patients were selected as ‘at-risk’ ethnicities. Patients were included if they were aged 18 and over, had registered with the GP in the last 5 years and did not have known infection with HBV or HCV. | 560 (Total)  200 (African) 170 (South Asian)  190 (Turkish) | *Coverage (%)*  2.3% (Total)  3% (African) 1.7% (South Asian)  2.1% (Turkish)  *Positivity (%)*  0% | *Quality score* NA  No major comments | The pilot was stopped prematurely due to insufficient uptake. This prevented a formal analysis of the benefit or harm of including HIV testing in the program. |

##### Table 8. Evidence base for the effectiveness of testing initiatives in hospital settings

| **Reference** | **Study characteristics** | **Testing approach** | **Study population and setting** | **Sample N** | **Outcomes** | **Critical appraisal** | **General comments** | |
| --- | --- | --- | --- | --- | --- | --- | --- | --- |
| **Emergency department only** | | | | | | | | |
| **HBV** | | | | | | | | |
| O'Connell 2016 [10] | *Country*  Ireland  *Study period*  March 2014 - Jan 2015  *Study design* Surveillance/evaluation/audit | Opt-out BBV screening programme consisting of an extra serum sample taken when undergoing phlebotomy as part of routine clinical care at no extra cost | All patients over the age of 18 with the capacity to consent, who had bloods taken as part of routine clinical care at a large urban Emergency Department in Dublin, Ireland | 10000 | *Coverage (%)*  88.4%  *Positivity (%)*  0.5%  *Newly diagnosed (%)*  0.2% | *Quality score* NA  No major comments | No comments | |
| Orkin, 2016 [11] | *Country*  UK  *Study period*  13-19 October 2014  *Study design* Surveillance/evaluation/audit | Routine opt-out testing offered for BBVs in Emergency department ('Going viral' campaign) | Adult emergency department attendees who had blood taken at nine emergency departments with HIV prevalence exceeding 2/1000, in London, Essex, Leeds and Glasgow | 7807 | *Coverage (%)*  27%  *Positivity (%)*  0.7%  *Newly diagnosed (%)*  0.5% | *Quality score* NA  Sample may not be representative of study population | - Uptake varied among emergency departments; some sites that tested larger numbers are overrepresented in results. - Five of the nine centres were in central London, making the results most applicable to inner London - It was not possible to distinguish between test not offered and refusal to test. | |
| **HCV** | | | | | | | | |
| O'Connell, 2016 [10] | *Country*  Ireland  *Study period*  March 2014 - Jan 2015  *Study design* Surveillance/evaluation/audit | Opt-out BBV screening programme consisting of an extra serum sample taken when undergoing phlebotomy as part of routine clinical care at no extra cost | All patients over the age of 18 with the capacity to consent, who had bloods taken as part of routine clinical care at a large urban Emergency Department in Dublin, Ireland | 10000 | *Coverage (%)*  88.4%  *Positivity (%)*  0,05%  *Newly diagnosed (%)*  0.6% | *Quality score*  NA  No major comments | No comments | |
| Orkin, 2016 [11] | *Country*  UK  *Study period*  13-19 October 2015  *Study design* Surveillance/evaluation/audit | Routine opt-out testing offered for BBVs in Emergency department ('Going viral' campaign) | Adult emergency department attendees who had blood taken at nine emergency departments with HIV prevalence exceeding 2/1000, in London, Essex, Leeds and Glasgow | 7807 | *Coverage (%)*  27%  *Positivity (%)*  1.8%  *Newly diagnosed (%)*  0.7% | *Quality score*  NA  Sample may not be representative of study population | - Uptake varied among emergency departments; some sites that tested larger numbers are overrepresented in results. - Five of the nine centres were in central London, making the results most applicable to inner London - It was not possible to distinguish between test not offered and refusal to test. | |
| **Other hospital departments** | | | | | | | |  |
| **HBV** | | | | | | | |  |
| Aparicio, 2012 [12] | *Country*  France  *Study period*  8 March-9 April 2010  *Study design* Surveillance/evaluation/audit | Targeted BBV screening for patients coming from high prevalence regions | Adult patients from sub-Saharan Africa, French Indies and French Guiana, consulting for a medical issue, dressing or blood sample in the outpatients department of Lariboisiere hospital in Paris | 272 | *Offered (%)*  100%  *Coverage (%)*  61%  *Positivity (%)*  7.8% | *Quality score* NA  No major comments | Article in French |  |
| Richter, 2014 [13] | *Country*  Netherlands  *Study period*  January 2011  *Study design* Surveillance/evaluation/audit | A screening project, involving a campaigning phase with posters and flyers and a website. FGMs received a personal invitation for an educational meeting with free onsite serological screening | FGMs aged >18 years from Afghanistan, Iran, Iraq, the former Soviet Republics and Vietnam living in Arnhem and Rheden invited to three different locations of Rijnstate Hospital | 3,226 registered FGM | *Offered (%)*  100%  *Coverage (%)*  28.7%  *Positivity (%)*  2.2% | *Quality score* NA  No major comments | No comments |  |
| Sanger, 2013 [14] | *Country*  UK  *Study period*  12 mo period  *Study design* Surveillance/evaluation/audit | A service improvement project, offering BBV tests. | Patients with severe mental illness in a central London psychiatric hospital (three open admission wards and one intensive care unit). | 105 | *Offered (%)*  83% had mental capacity to make informed decision  *Coverage (%)*  54%  *Positivity (%)*  7.0%  *Newly diagnosed (%)*  7.0%  *Acceptance rate (%)*  66% | *Quality score* NA  No major comments | No comments |  |
| **HCV** | | | | | | | |  |
| Parisi, 2014 [4] | *Country*  Italy  *Study period*  Jan 2011 - Apr 2014  *Study design* Surveillance/evaluation/audit | A Prevention Program called “EASY test project” using two new oral tests to diagnose the HCV and HIV infection. | People aged >18 years, unaware of their HCV serological status and able to complete the questionnaire in Italian or English who attended two Points-of-care and one HIV-STDs public prevention outpatient clinic (“FreeDay Easy”) of the Infectious Diseases Department of San Raffaele Scientific Institute in Milan and extended to six general practitioner surgeries. | 29,600 (total)  14,000 (2011) 10,000 (2012) 5,000 (2013) 600 (2014) | *Coverage (%)*  15.2% (total) 9.1% (2011) 14% (2012) 16.9% (2013) 22% (2014)  *Positivity (%)*  0.6% (total) 0.5% (2011) 0.5% (2011) 0.4% (2013) 4.5% (2014) | *Quality score* NA  No major comments | No comments |  |
| McLeod, 2014 [6] | *Country*  UK  *Study period*  Jan 1999 - Dec 2011  *Study design*  Pre-post study | Surveillance study on HCV testing examining impact of the Hepatitis C Action Plan consisting of awareness-raising activities, for GPs and those at risk, and the introduction of DBS sampling in community drug services to overcome barriers to testing. | Data on anti-HCV tests provided by the West of Scotland Specialist Virology Centre; East of Scotland Specialist Virology Centre; Department of Medical Microbiology at Aberdeen Royal Infirmary and Ninewells Hospital & Medical School. Positive results were laboratory confirmed. | 145,140(total) 10,536 (pre-Action Plan) 12,170 (Action Plan) | *Positivity (%)*  35.5% (total) 44.4% (pre-Action Plan) 27.0% (Action Plan) | *Quality score* NA  No major comments | RR also reported for initial trend, level change in number tested and change in trend over each setting |  |
| Aparicio, 2012 [12] | *Country*  France  *Study period*  8 March-9 April 2010  *Study design* Surveillance/evaluation/audit | Targeted BBV screening for patients coming from high prevalence regions | Adult patients from sub-Saharan Africa, French Indies and French Guiana, consulting for a medical issue, dressing or blood sample in the outpatients department of Lariboisiere hospital in Paris | 272 | *Offered (%)*  100%  *Coverage (%)*  61%  *Positivity (%)*  3.6% | *Quality score* NA  No major comments | Article in French |  |
| Richter, 2014 [13] | *Country*  Netherlands  *Study period*  January 2011  *Study design* Surveillance/evaluation/audit | A screening project, involving a campaigning phase with posters and flyers and a website. FGMs received a personal invitation for an educational meeting with free onsite serological screening | FGMs aged >18 years from Afghanistan, Iran, Iraq, the former Soviet Republics and Vietnam living in Arnhem and Rheden invited to three different locations of Rijnstate Hospital | 3,226 registered FGM | *Offered (%)*  100%  *Coverage (%)*  28.7%  *Positivity (%)*  0.3% <0.1% (HCV-RNA) | *Quality score* NA  No major comments | No comments |  |
| Sanger, 2013 [14] | *Country*  UK  *Study period*  12 mo period  *Study design* Surveillance/evaluation/audit | A service improvement project, offering BBV tests. | Patients with severe mental illness in a central London psychiatric hospital (three open admission wards and one intensive care unit). | 105 | *Offered (%)*  83% had mental capacity to make informed decision  *Coverage (%)*  54%  *Positivity (%)*  8.7%  *Acceptance rate (%)*  66% | *Quality score* NA  No major comments | No comments |  |

##### Table 9. Evidence base for the effectiveness of testing initiatives in other healthcare settings

| **Reference** | **Study characteristics** | **Testing approach** | **Study population and setting** | **Sample N** | **Outcomes** | **Critical appraisal** | **General comments** | |
| --- | --- | --- | --- | --- | --- | --- | --- | --- |
| **Antenatal services** | | | | | | | | |
| **HBV** | | | | | | | | |
| Keel, 2016 [15] | Country  UK  Study period  Baseline retrospective review: 1 Jan 2009 - 31 Dec 2009  Prospective intervention period: 1 Nov 2010 - 31 Dec 2011  Study design  Non-randomised trial | During the **baseline period**, a retrospective review was carried out of GP records of pregnant women identified through the Enhanced Surveillance of Antenatal Hepatitis B database. The relevant GP was sent a letter explaining the purpose of the review and a questionnaire requesting further information on the case and their household contacts. North Middlesex acted as the prospective intervention arm and Newham acted as the prospective comparison arm where service provision remained unchanged from the baseline review period. In the **intervention group**, women whose household contacts were eligible for home DBS testing were identified by weekly meetings. DBS testing and questionnaire were collected. | Household contacts of all HBsAg-positive pregnant women identified through antenatal screening at two maternity units in London that were selected based on their high antenatal HBV prevalence (North Middlesex and Newham hospitals) during the review and intervention periods. HBsAg-positive pregnant women were excluded if (a) they were known to be positive prior to antenatal screening and their families had been tested and vaccinated appropriately; or (b) their care was transferred to a trust not participating in the intervention. | Number of households:  Middlesex retrospective: 41 (71.9% of pregnant women) Middlesex prospective intervention: 58 (100% of pregnant women) Newham retrospective: 91 (73.4% of pregnant women)  Newham prospective control: 68 (55.7% of pregnant women)   Relationship: child; partner; other adult  Middlesex retrospective: 54; 33; 4 Middlesex prospective intervention: 90; 56; 23 Newham retrospective: 65; 81; 24 Newham prospective control: 63; 39; 59 | *Coverage (%)*  Any household tested:  Middlesex retrospective: 62.1%  Middlesex prospective intervention: 96.6% Newham retrospective: 39.6% Newham prospective control: 39.4%  Relationship: child; partner; other adult  Middlesex retrospective: 57.4%; 30.3%; 25% Middlesex prospective intervention: 100%; 96.4%; 100% Newham retrospective: 64.6%; 29.6%; 25% Newham prospective control: 31.7%; 23.1%; 30.5%  *Positivity (%)*  Current infection: Total: (relationship:child; partner; other adult) 2.9%; 10.0%; 10.9%  Relationship: child; partner; other adult Middlesex retrospective: 0%; 3%; 0% Middlesex prospective intervention: 3.3%; 21.4%; 21.7% Newham retrospective: 6.2%; 7.4%; 8.3%  Newham prospective control: 1.6%; 5.1%; 8.5% | *Quality score* Acceptable  No major comments | Denominator differs | |
| **HCV** | | | | | | | | |
| Diab-Elschahawi, 2013 [16] | *Country*  Austria  *Study period*  1 July 2007 - 28 Feb 2011  *Study design*  Pre-post study | Comparison of a targeted screening approach vs universal screening. During the first 22 months of the study a targeted HCV screening approach was adopted. Universal screening was implemented for the following 22 months. | Pregnant women presenting for antenatal care at the Vienna University Hospital (tertiary-care medical university hospital), Vienna, Austria | 4,369 (targeted screening); 4,222 (universal screening) | *Coverage (%)*  28.3% (targeted screening)  *Positivity (%)*  1.3% (targeted screening); 1.7% (universal screening) | *Quality score* NA  No major comments | The population of pregnant women is mostly patients at risk for pregnancy-related complications, including those enrolled in opiate maintenance therapy programs | |
| **Clinic for precarious individuals** | | | | | | | | |
| **HBV** | | | | | | | | |
| Pauti, 2009 [17] | *Country*  France  *Study period*  2007  *Study design* Surveillance/evaluation/audit | Systematic screening for all new patients as part of the "Doctors of the World France mission" facilitating access to care for vulnerable populations. The project includes staff training, organisation of multidisciplinary teams, individual prevention interviews conducted with patients prior to consultations (at Saint-Denis; in Paris, awareness information was provided in the waiting room) and use of interpreters. | All new patients (mainly immigrants) attending two Reception Centres for Care and Orientation (RCCO) in Saint-Denis and Paris | 1205 tests performed | *Coverage (%)*  At Saint-Denis RCCO: 90% when screening is proposed during a prevention interview; 71% without interview.  *Positivity (%)*  6.9% | *Quality score* NA  Methods and results were not clearly described | - 90% of study population were immigrants - Before October 2007, no free interpretation was provided and this meant that mainly French-speaking subjects were reached - Methods and results were often vague, it is not clear why only some results are shown for certain centres | |
| **HCV** | | | | | | | | |
| Pauti, 2009 [17] | *Country*  France  *Study period*  2007  *Study design* Surveillance/evaluation/audit | Systematic screening for all new patients as part of the "Doctors of the World France mission" facilitating access to care for vulnerable populations. The project includes staff training, organisation of multidisciplinary teams, individual prevention interviews conducted with patients prior to consultations (at Saint-Denis; in Paris, awareness information was provided in the waiting room) and use of interpreters. | All new patients (mainly immigrants) attending two Reception Centres for Care and Orientation (RCCO) in Saint-Denis and Paris | 1205 tests performed | Coverage (%)  At Saint-Denis RCCO: 90% when screening is proposed during a prevention interview; 71% without interview.  Positivity (%)  5.8% | *Quality score* NA  Methods and results were not clearly described | - 90% of study population were immigrants - Before October 2007, no free interpretation was provided and this meant that mainly French-speaking subjects were reached - Methods and results were often vague, it is not clear why only some results are shown for certain centres | |
| **Drug services** | | | | | | | | |
| **HBV** | | | | | | | | |
| Schreuder, 2010 [18] | *Country*  Netherlands  *Study period*  2004 - 2008 (Amsterdam) 2003 -2009 (Heerlen)  *Study design* Surveillance/evaluation/audit | Voluntary screening for infectious diseases at methadone posts | Opioid drug users (ODU) attending methadone posts in Amsterdam and Heerlen, the Netherlands | 2024 (Amsterdam) 287 (Heerlen) | *Coverage (%)*  34% (680/2024)(Amsterdam) 69% (197/287)(Heerlen)  *Positivity (%)*  33% (anti-HBc Amsterdam) 48% (anti-HBc Heerlen) Newly diagnosed (%) | *Quality score* NA  No major comments | No comments | |
| **HCV** | | | | | | | | |
| Arain, 2016 [19] | *Country*  Belgium  *Study period*  Feb 2014 - Dec 2014  *Study design*  RCT | Pilot study combining formal education, peer education and FibroScan assessment. The participants were randomized into two groups: the **control group**, who received the current standard of care (the availability of information brochures in the waiting room was announced), and the **intervention group**, who received an information session (1 hour meeting with powerpoint presentation, followed by additional information from peers over personal experiences of treatment) followed by a FibroScan at a hospital (transport provided). | Persons who use drugs age ≥18 years with a history of substance use and attending opioid substitution treatment program at the Center for Alcohol and other Drug problems located in Limburg, Belgium (comprised of former and current substance users). | 52 (27 control 25 intervention) 17 completed all questionnaires | *Coverage (%)*  7% (2/27) control 20% (5/25) intervention | *Quality score* acceptable  No major comments | The study was single blind, as it was impossible to blind the research team due to the researcher's involvement in the information session and its coordination. | |
| Lindenburg, 2011 [20] | *Country*  Netherlands  *Study period*  Jan 2005 - Apr 2007  *Study design* Surveillance/evaluation/audit | Drug Users Treatment for Chronic Hepatitis-C (DUTCH-C) project offering HCV screening and treatment in a multidisciplinary unit established for this purpose, including ACS medical staff, a liver specialist, a psychiatrist, and a virologist from the Amsterdam Medical Center (AMC) and addiction specialists and case-load managers from methadone clinics. | Active and former drug users (DU), injecting or not, participating in the Amsterdam Cohort Study (ACS), visiting from December 2004 the Public Health Service Amsterdam (PHSA) (population 1). In 2007 non-ACS DU, either been tested HCV positive before or were considered to have a high likelihood of being infected, referred from methadone clinics and other addiction clinics in Amsterdam, were also included (population 2). | 497 (population 1) 81 (population 2) | *Coverage (%)*  90% (population 1) 98% (population 2)  *Positivity (%)*  60% (overall) 54% (HIV- overall) 89% (HIV+ overall) Newly diagnosed (%) | *Quality score* NA  Study population not clearly described | The study population is not clearly described - the text suggests that only people with a background of psychiatric co-morbidity and social problems are included, however from the data this does not appear to be the case | |
| Schreuder, 2010 [18] | *Country*  Netherlands  *Study period*  2004 - 2008 (Amsterdam) 2003 -2009 (Heerlen)  *Study design* Surveillance/evaluation/audit | Voluntary screening for infectious diseases at methadone posts | Opioid drug users (ODU) attending methadone posts in Amsterdam and Heerlen, the Netherlands | 2566 (Amsterdam) 287 (Heerlen) | *Coverage (%)*  53% (1359/2566)(Amsterdam) 66% (190/287)(Heerlen)  *Positivity (%)*  26% (Amsterdam) 61% (Heerlen) Newly diagnosed (%) | *Quality score* NA  No major comments | No comments | |
| Tait, 2013 [21] | *Country*  UK  *Study period*  2009-2011  *Study design* Surveillance/evaluation/audit | DBS testing offered to all individuals who accessed needle exchange (NEXC) or drug treatment services (DTS) | Drug users living in Tayside who accessed needle exchange (NEXC) or drug treatment services (DTS) | Tested:  1123 total 631 NEXC 492 DTC | *Coverage (%)*  84.2% 18.7% (follow-up test within 1 y after the first)  *Positivity (%)*  31.2% total 27.5% NEXC 35.3% DTC  *Newly diagnosed (%)*  22.6% total 22.4% NEXC 22.8% DTC | *Quality score* NA  No major comments | No comments | |
| **Free clinics** | | | | | | | | |
| **HBV** | | | | | | | | |
| Bottero, 2015 [22] | *Country*  France  *Study period*  25 Feb 2013 - 21 June 2013  *Study design*  RCT | A randomized, prospective, pilot intervention trial (The Optiscreen III study). Eligible participants were randomized to receive 1 of 2 testing interventions for HIV, HBV, and HCV infection: a standard serology-based test (S arm), where participants received a prescription for serology performed at an outside laboratory; or a Point-of-Care (POC) rapid test (RT arm). | Individuals seeking care at an innercity clinic for persons without healthcare coverage (“Médecins du Monde”, Paris, France) were asked to participate if they were ≥18 years old and could be available for further medical follow-up testing at Hôpital Saint-Antoine (Paris, France), if necessary. Persons already followed for HIV, HBV, and/or HCV infection or persons whose HIV, HBV, and/or HCV test result from 3 months before inclusion was available were excluded. | 162 (S arm) 162 (RT arm) | *Coverage (%)*  64.2% (S arm) 98.2% (RT arm)  *p*<0.001  *Positivity (%)*  8.3% (95% CI 5.4%-12.2%) Total  9.6% S arm  8.1% RT arm  *Patient indicators*  Proportion who prefer rapid testing to standard serological tests: 76%  Proportion who preferred serological tests vs. had no preference: 7% vs 17%  Common reasons for preferring rapid tests: less stress with same-day results, more practical use.  Reasons for preferring serological tests: able to perform several tests at once, felt that the test was more reliable/accurate or caused less anxiety.  6 rapid test failures and 6 patients unable to be tested due to over calloused finger tips.  *Provider indicators*  Proportion that said that rapid testing simplified their consultation: 50%  Proportion that claimed it had no effect: 35%  Proportion that stated that it became more complicated: 15% | *Quality score* high  No major comments | No comments | |
| **HCV** | | | | | | | | |
| Bottero, 2015 [22] | *Country*  France  *Study period*  25 Feb 2013 - 21 June 2013  *Study design*  RCT | A randomized, prospective, pilot intervention trial (The Optiscreen III study). Eligible participants were randomized to receive 1 of 2 testing interventions for HIV, HBV, and HCV infection: a standard serology-based test (S arm), where participants recieved a prescription for serology performed at an outside laboratory; or a Point-of-Care (POC) rapid test (RT arm). | Individuals seeking care at an innercity clinic for persons without healthcare coverage (“Médecins du Monde”, Paris, France) were asked to participate if they were ≥18 years old and could be available for further medical follow-up testing at Hôpital Saint-Antoine (Paris, France), if necessary. Persons already followed for HIV, HBV, and/or HCV infection or persons whose HIV, HBV, and/or HCV test result from 3 months before inclusion was available were excluded. | 162 (S arm) 162 (RT arm) | *Coverage (%)*  64.2% (S arm) 98.2% (RT arm)  *p*<0.001  *Positivity (%)*  2.9% (95% CI 1.3% - 5.6%) Total  3.8% S arm  2.5% RT arm  *Patient indicators*  Proportion who prefer rapid testing to standard serological tests: 76%  Proportion who preferred serological tests vs. had no preference: 7% vs 17%  Common reasons for preferring rapid tests: less stress with same-day results, more practical use.  Reasons for preferring serological tests: able to perform several tests at once, felt that the test was more reliable/accurate or caused less anxiety.  6 rapid test failures and 6 patients unable to be tested due to over calloused finger tips.  *Provider indicators*  Proportion that said that rapid testing simplified their consultation: 50%  Proportion that claimed it had no effect: 35%  Proportion that stated that it became more complicated: 15% | *Quality score* high  No major comments | No comments | |
| **Migrant clinics** | | | | | | | | |
| **HBV** | | | | | | | | |
| Nosotti, 2016 [23] | *Country*  Italy  *Study period*  March 2013 - June 2014  *Study design* Surveillance/evaluation/audit | Blood HBV test and vaccination offered to immigrants. | Immigrants coming from different high and intermediate endemic areas accessing the Outpatient Clinic of National Institute for Health, Migration and Poverty. | 516 | *Coverage (%)*  87%  *Positivity (%)*  7.7% | *Quality score* NA  No major comments | No comments | |
| El-Hamad, 2015 [24] | *Country*  Italy  *Study period*  Jan 2006 - Apr 2010  *Study design* Surveillance/evaluation/audit | Point of care screening | All consecutive migrants who accessed the Service of International Medicine of Brescia’s Local Health Authority, coming from non-EU and non-western countries and newly accessing the Service of International Medicine, aged more than 18 years, and able to give a written consent. | 4,078 | *Coverage (%)*  91.4%  *Positivity (%)*  6.0% (HbsAg) 43.3% (HBcAb) 1.6% (HBeAg) | *Quality score* NA  No major comments | No comments | |
| **HCV** | | | | | | | | |
| El-Hamad, 2015 [24] | *Country*  Italy  *Study period*  Jan 2006 - Apr 2010  *Study design* Surveillance/evaluation/audit | Point of care screening | All consecutive migrants who accessed the Service of International Medicine of Brescia’s Local Health Authority, coming from non-EU and non-western countries and newly accessing the Service of International Medicine, aged more than 18 years, and able to give a written consent. | 4,078 | *Coverage (%)*  90.8%  *Positivity (%)*  3.6% | *Quality score* NA  No major comments | No comments | |
| **Pharmacies** | | | | | | | | |
| **HCV** | | | | | | | | |
| Radley, 2017 [25] | *Country*  UK  *Study period*  Jan 2014 - Dec 2014  *Study design*  Non-randomised trial | Comparison of non-randomly chosen **intervention** groups (DBST in ORT pharmacies) and **control** groups (HCV testing in any other setting) for access and uptake of DBST. | Population receiving opioid replacement therapy (ORT) attending six ORT pharmacies offering dried blood spot testing (DBST) in the intervention group and ORT service users accessing HCV testing from 36 community ORT pharmacies (which did not offer DBST) in the city of Dundee, Scotland. Pharmacies were selected if they provided OST supervision for at least 30 patients and staff was willing to participate. | 143 (intervention) 561 (control) | *Coverage (%)*  30% (43/143)(intervention)  13% (75/561) (control) OR: 2.25 (95% CI 1.48 - 3.42)  *Patient indicators*  Pharmacies found to be good place to be tested, positive relationships built with staff. Pharmacies viewed as part of the local community. Some suspicious when offered testing due to previous experience of discrimination at pharmacy. Some clients wished for better explanation of how DBS test worked  *Provider indicators*  Strong leadership, team involvement important for success of pharmacy DBS testing. Initial anxieties about contact with blood and concerns about workload. DBST was found to be simple to perform | *Quality score* acceptable  No major comments | No comments | |
| Radley, 2017 [26] | *Country*  UK  *Study period*  Nov 2015-Sept 2016  *Study design*  RCT | RCT evaluating uptake of DBS testing and treatment in a conventional care pathway vs. Pharmacist-led pathway. - Conventional care pathway: the pharmacist opportunistically discussed HCV testing with patients and performed DBS if the patient consented and had no recent test. Patients testing positive were invited to attend a clinic where standard care was carried out. - Pharmacist-led pathway: Identical to conventional, but patients testing positive were assessed by the pharmacist for treatment. If suitable, patients received treatment daily at the same time as their OST. | Patients prescribed opioid substitution therapy (OST) at eight pharmacies within the Tayside region of Scotland | 244 (conventional pathway); 262 (pharmacist-led pathway) | *Coverage (%)*  24% (58/244) (conventional) 36% (94/262) (pharmacist-led)  *Positivity (%)*  25.9% (conventional) 26.5% (pharmacist-led) | *Quality score* acceptable  No major comments | Need to attend for off-site phlebotomy for confirmation led to some loss of potential patients in the pharmacist-led pathway | |
| **Prisons** | | | | | | | | |
| **HBV** | | | | | | | | |
| Patel, 2016 [27] | *Country*  UK  *Study period*  NR  *Study design* Surveillance/evaluation/audit | A walk-in blood spot service advertised and provided to prisoners | Prisoners at one medium security prison | 160 male prisoners tested | *Positivity (%)*  0% 1.3% anti-HBc+ | *Quality score* NA  No major comments | No comments | |
| Sagnelli, 2012 [28] | *Country*  Italy  *Study period*  NR  *Study design* Surveillance/evaluation/audit | Screening project based on a peer-to-peer communication, followed by a month of blood sampling (on a voluntary basis) to test for HCV, HBV, HIV, Syphilis and Latent Tuberculosis. | Prisoners of 9 Italian prisons | 3,468 | *Coverage (%)*  65.3%  *Positivity (%)*  4.4%  *Newly diagnosed (%)*  1.5% | *Quality score* NA  Method section is limited | No comments | |
| **HCV** | | | | | | | | |
| McLeod, 2014 [6] | | *Country*  UK  *Study period*  Jan 1999 - Dec 2011  *Study design*  Pre-post study | Surveillance study on HCV testing examining impact of the Hepatitis C Action Plan consisting of awareness-raising activities, for GPs and those at risk, and the introduction of DBS sampling in community drug services to overcome barriers to testing. | Data on anti-HCV tests provided by the West of Scotland Specialist Virology Centre; East of Scotland Specialist Virology Centre; Department of Medical Microbiology at Aberdeen Royal Infirmary and Ninewells Hospital & Medical School. Positive results were laboratory confirmed. | 4,200 (total) 257 (pre-Action Plan) 429 (Action Plan) | *Positivity (%)*  4.2% (total) 5.2% (pre-Action Plan) 3.5% (Action Plan) | *Quality score* NA  No major comments | RR also reported for initial trend, level change in number tested and change in trend over each setting |
| Sagnelli, 2012 [28] | | *Country*  Italy  *Study period*  NR  *Study design* Surveillance/evaluation/audit | Screening project based on a peer-to-peer communication, followed by a month of blood sampling (on a voluntary basis) to test for HCV, HBV, HIV, Syphilis and Latent Tuberculosis. | Prisoners of 9 Italian prisons | 3,468 | *Coverage (%)*  64.6%  *Positivity (%)*  22.8%  *Newly diagnosed (%)*  1.5% | *Quality score* NA  Method section is limited | No comments |
| Patel, 2016 [27] | | *Country*  UK  *Study period*  NR  *Study design* Surveillance/evaluation/audit | A walk-in blood spot service advertised and provided to prisoners | Prisoners at one medium security prison | 160 male prisoners tested | *Positivity (%)*  33.8% 23.8% HCV-RNA+ | *Quality score* NA  No major comments | No comments |
| Craine, 2015 [29] | | *Country*  UK  *Study period*  March 2011 - Sept 2012  *Study design*  RCT | A stepped wedge cluster RCT design with the intervention being randomized by start date. The **intervention** was the offer of DBST for HCV to prisoners. | All prisoners, able to give informed consent for diagnostic testing, of five UK prisons (4/5 prisons never implemented routine HCV testing and 1 prison carried out routine HCV testing services (venepuncture) before the study) | ≈ 3,600 | ORs for effect of the intervention on testing rate:  Intention to treat (ITT): date is set as planned  Actual intervention: the date it occurred  OR: 0.84; 95% CI: 0.68-1.03; P=0.088 (ITT)  OR: 0.86; 95% CI: 0.71-1.06; P=0.153 (Actual interventions)  OR: 0.77; 95% CI: 0.53-1.13; P=0.157 (ITT pooled results of 5 imputations)  OR: 0.80; 95% CI: 0.55-1.15; P=0.192 (Actual interventions pooled results of 5 imputations) | *Quality score* acceptable  No major comments | Article presents figure with total test rate over time with fitted LOESS curve for each prison and for all five prisons. |
| **Specialist services** | | | | | | | | |
| **HCV** | | | | | | | | |
| McAllister, 2014 [30] | | *Country*  UK  *Study period*  May 2009-Dec 2010  *Study design* Surveillance/evaluation/audit | DBS testing introduced to Scotland as part of the Hepatitis C Action Plan for Scotland | Individuals (mainly PWID) across Scotland who were tested by DBS | 1322 | *Positivity (%)*  55% 36% (active infection) | *Quality score* NA  Study population not clearly described | 4.6% of the positive-testing population is non-PWID. It is not clear from the study methods if they are other drug users or why they were tested by DBS. The rest of the article insinuates that the DBS testing programme was focused on PWID, but this is not actually stated. |
| **STI clinics** | | | | | | | | |
| **HBV** | | | | | | | | |
| Richens, 2010 [31] | | *Country*  UK  *Study period*  June 2005 - July 2006  *Study design*  RCT | A two-centre parallel three-arm randomised controlled open trial named The Computer Assisted Sexual Health Interviewing (CASHI) study. Patients were randomly assigned to be interviewed in one of three ways: 1. **Computer-assisted self-interview (CASI)** 2. **Computer-assisted personal interview (CAPI)**, patient and clinician viewing the screen together, using the same interview as in CASI, but with data input by the clinician. 3. **Pen and paper interview (PAPI)** with a clinician following the normal clinic practice of completing a proforma with the patient (usual care arm). | Patients over the age of 16 years with a new clinical episode (suggesting STI) attending two large London sexual health clinics, the Mortimer Market Centre and the Courtyard Clinic. | 2480 assessed to be eligible (total) 2318 participated (total) 801 allocated; 795 analyzed (CASI) 763 allocated; 744 analyzed (CAPI) 787 allocated; 779 analyzed (PAPI) | *Coverage (%)*  24.2% (CAPI) 16.9% (CASI) 16.3% (PAPI)  *Patients indicators*  Computer-assisted interviewing appeared to encourage the disclosure of sexual risk-taking. | *Quality score* acceptable  No major comments | - Not all participants received the intervention that they were randomly allocated to. - Many different formats for electronic interviews are possible, such as the wording of questions and whether respondents are given freedom to skip questions, which are likely to affect response rates.  - Clinicians seeing patients recruited into the CASI and CAPI arms of the study were required to conduct consultations in a way that was new and different (and in the case of CAPI, rather unpopular). If conducted in an environment where these new approaches were more familiar and established, it is likely that this might have produced different results. |
| **HCV** | | | | | | | | |
| Parisi, 2014 [4] | | *Country*  Italy  *Study period*  Jan 2011 - Apr 2014  *Study design* Surveillance/evaluation/audit | A Prevention Program called “EASY test project” using two new oral tests to diagnose the HCV and HIV infection. | People aged >18 years, unaware of their HCV serological status and able to complete the questionnaire in Italian or English who attented two Points-of-care and one HIV-STDs public prevention outpatient clinic (“FreeDay Easy”) of the Infectious Diseases Department of San Raffaele Scientific Institute in Milan and extended to six general practitioner surgeries. | 29,600 (total) 14000 (2011) 10000 (2012) 5000 (2013) 600 (2014) | *Coverage (%)*  15.2% (total) 9.1% (2011) 14% (2012) 16.9% (2013) 22% (2014)  *Positivity (%)*  0.6% (total) 0.5% (2011) 0.5% (2011) 0.4% (2013) 4.5% (2014) Newly diagnosed (%) | *Quality score* NA  No major comments | No comments |
| McLeod, 2014 [6] | | *Country*  UK  *Study period*  Jan 1999 - Dec 2011  *Study design*  Pre-post study | Surveillance study on HCV testing examining impact of the Hepatitis C Action Plan consisting of awareness-raising activities, for GPs and those at risk, and the introduction of DBS sampling in community drug services to overcome barriers to testing. | Data on anti-HCV tests provided by the West of Scotland Specialist Virology Centre; East of Scotland Specialist Virology Centre; Department of Medical Microbiology at Aberdeen Royal Infirmary and Ninewells Hospital & Medical School. Positive results were laboratory confirmed. | 33,677 (total) 1,699 (pre-Action Plan) 4,018 (Action Plan) | *Positivity (%)*  15.5% (total) 19.6% (pre-Action Plan) 10.1% (Action Plan) | *Quality score* NA  No major comments | RR also reported for initial trend, level change in number tested and change in trend over each setting |
| Murira, 2016 [32] | | *Country*  UK  *Study period*  Oct 2013 - Oct 2014  *Study design*  Pre-post study | Introduction of a clinic specific guideline in April 2014 for Hep C screening. | All genitourinary medicine (GUM) clinic attendees who were born in a country of Hep C prevalence of >2%. All HIV positive individuals were excluded. | 2,664 (total) 1,299 (6 mo pre guidline) 1,354 (6 mo post guidline) | *Coverage (%)*  4.7% (6 mo pre guidline) 13.6% (6 mo post guidline) 2.88 times increase | *Quality score* NA  No major comments | No comments |
| Scott, 2010 [33] | | *Country*  UK  *Study period*  1 Jan 2007 - 31 June 2007  *Study design* Surveillance/evaluation/audit | Unselected anti-HCV screening for MSM attending sexual health screens introduced in clinics | All MSM unaware of their HCV status attending three sexual health clinics at the Chelsea and Westminster hospital. MSM who reported previous HCV infection were excluded from screening. | 3365 attending STI screens 325 HIV positive | *Coverage (%)*  69%  *Positivity (%)*  0.65% (95% CI 0.36%-1.1%) 0.88% HIV positive group (95% CI 0.18%-2.6%) | *Quality score* NA  Method section is limited | No comments |
| Richens, 2010 [31] | | *Country*  UK  *Study period*  June 2005 - July 2006  *Study design*  RCT | A two-centre parallel three-arm randomised controlled open trial named The Computer Assisted Sexual Health Interviewing (CASHI) study. Patients were randomly assigned to be interviewed in one of three ways: 1. **Computer-assisted self-interview (CASI)** 2. **Computer-assisted personal interview (CAPI)**, patient and clinician viewing the screen together, using the same interview as in CASI, but with data input by the clinician. 3. **Pen and paper interview (PAPI)** with a clinician following the normal clinic practice of completing a proforma with the patient (usual care arm). | Patients over the age of 16 years with a new clinical episode (suggesting STI) attending two large London sexual health clinics, the Mortimer Market Centre and the Courtyard Clinic. | 2480 assessed to be eligible (total) 2318 participated (total) 801 allocated; 795 analyzed (CASI) 763 allocated; 744 analyzed (CAPI) 787 allocated; 779 analyzed (PAPI) | *Coverage (%)*  8.9% (CAPI) 3.3% (CASI) 2.8% (PAPI) | *Quality score* acceptable  No major comments | - Not all participants received the intervention that they were randomly allocated to. - Many different formats for electronic interviews are possible, such as the wording of questions and whether respondents are given freedom to skip questions, which are likely to affect response rates.  - Clinicians seeing patients recruited into the CASI and CAPI arms of the study were required to conduct consultations in a way that was new and different (and in the case of CAPI, rather unpopular). If conducted in an environment where these new approaches were more familiar and established, it is likely that this might have produced different results. |
| **Mixed settings (not stratified)** | | | | | | | | |
| **HCV** | | | | | | | | |
| Bishton, 2014 [34] | | *Country*  UK  *Study period*  18 months  *Study design* Surveillance/evaluation/audit | DBS testing offered for HCV, HBV and HIV, and vaccination offered for HBV | All who considered themselves at risk and current tier 3 drug treatment clients (including alcohol clients with a previous history of drug use), clients at the tier 2 drug treatment needle exchange, at the needle exchange at the local homeless clinic and at the two pharmacy needle-exchange sites with the highest current usage located in North East Essex. | NR | *Coverage*  266 tested  *Positivity (%)*  35%  53% (of % positive had active infection) | *Quality score* NA  Method section is limited and no structure in the article | Short communication article |
| Defossez, 2008 [35] | | *Country*  France  *Study period*  1 Nov 1997 - 31 Dec 1997 1 Feb 2000 - 31 March 2000 1 Feb 2003 - 31 May 2003  *Study design*  Cross-sectional | Repeated comparative cross-sectional survey of HCV testing at three different time points to document trends in screening practises since implementation of a national plan to promote screening, including campaigns and screening guideline and set up of a network | Population living in the Poitou-Charentes region of southwest France who had their data tested or collected at private and public medical laboratories and prescribing physicians. | Unclear   (1640453 1997 census data) (1649804 2000 census data) (1677855 2003 census data) | *Coverage (%)*  2.3% 1997 2.6% 2000 3.7% 2003  *Newly diagnosed (%)*  53% (69/130) 1997 55% (58/106) 2000 44% (96/216) 2003 | *Quality score* NA  Denominator was not clear | No comments |
| Hickman, 2008 [36] | | *Country*  UK  *Study period*  Intervention start date: June - Dec 2004 (duration 6 mo)  *Study design*  RCT | A cluster randomized controlled trial. Sites were matched into pairs, with one site in each pair randomly allocated. **Intervention** sites: offered dried blood spot for diagnostic HCV antibody testing. **Control** sites continued with their current HCV testing practice: either testing patients on request or at selected times each week when specialist staff were available or referring patients elsewhere. | 28 (14 pairs) specialist drug clinics and six (three pairs) prisons throughout England and Wales | 6,550 (intervention) 5,800 (control) | *Coverage (%)*  Intervention 8.4% (6 mo before intervention)  20.6% (during intervention)  Control 7.7% (6 mo before intervention) 5.4% (during intervention)  % difference intervention vs control: 14.5% (95% CI 1.3–28%, paired t-test, P = 0.033)  *Positivity (%)*  Overall from both intervention and control sites HCV positive antibodies were detected in 320 of the 1034 (32%) of the patients during the trial. During the same period in 11 of the laboratories, there were a total of 2700 of 51 000 (5%) specimens that tested HCV positive. | *Quality score* low  No major comments | No baseline characteristics table and therefore don't know if groups are comparable |

##### Table 10. Evidence base for the effectiveness of testing initiatives in community settings

| **Reference** | **Study characteristics** | **Testing approach** | **Study population and setting** | **Sample N** | **Outcomes** | **Critical appraisal** | **General comments** |
| --- | --- | --- | --- | --- | --- | --- | --- |
| **Community based testing sites** | | | | | | | |
| **HBV** | | | | | | | |
| Tafuri, 2010 [37] | *Country*  Italy  *Study period*  May 2008 - July 2008  *Study design* Surveillance/evaluation/audit | Voluntary screening offered for HBV, HCV, HIV and syphillis. | A population of refugees of various nationalities who were in apparent good health and did not report signs or symptoms in the recent or remote past, living in the Asylum Seeker Centre in Bari Palese in Puglia, Southern Italy. | 744 | *Coverage (%)*  71.1% (529/744)  *Positivity (%)*  8.3% 45.6% (anti-HBc) | *Quality score* NA  No major comments | No comments |
| Okpo, 2015 [38] | *Country*  UK  *Study period*  freshers week in autumn 2013 and spring 2014  *Study design* Surveillance/evaluation/audit | DBS testing offered for BBV. Information packs and leaflets were provided to the students before testing. | New students registering at two universities in Aberdeen | 512 | *Coverage (%)*  37%  *Positivity (%)*  9.4%  3.1% (current infection)  *Acceptance rate (%)*  37%  *Patient indicators*  No reports by students that the offer or testing process was stigmatising or undesirable  *Provider indicators*  No reports by staff that the offer or testing process was stigmatising or undesirable | *Quality score* NA  Limited method section | No comments |
| **HCV** | | | | | | | |
| Selvapatt, 2015 [39] | *Country*  UK  *Study period*  8 July 2014 - 22 Jan 2015  *Study design* Surveillance/evaluation/audit | Outreach screening using oral swab testing followed by direct linkage into local hepatology clinic without need for a GP referral | Homeless visiting the St Mungo’s Centre for the Homeless, London | 32 completed questionnaire | *Coverage*  95 were tested  *Positivity (%)*  63%  1% HIV coinfected  0% HBV coinfected  *Patient indicators*  Would agree to screening: 91%  Thought oral swab test was acceptable form of testing: 91% | *Quality score* NA  No major comments | No comments |
| Tafuri, 2010 [37] | *Country*  Italy  *Study period*  May 2008 - July 2008  *Study design* Surveillance/evaluation/audit | Voluntary screening offered for HBV, HCV, HIV and syphillis. | A population of refugees of various nationalities who were in apparent good health and did not report signs or symptoms in the recent or remote past, living in the Asylum Seeker Centre in Bari Palese in Puglia, Southern Italy. | 744 | *Coverage (%)*  71.1% (529/744)  *Positivity (%)*  4.7% | *Quality score* NA  No major comments | No comments |
| Okpo, 2015 [38] | *Country*  UK  *Study period*  freshers week in autumn 2013 and spring 2014  *Study design* Surveillance/evaluation/audit | DBS testing offered for BBV. Information packs and leaflets were provided to the students before testing. | New students registering at two universities in Aberdeen | 512 | Coverage (%)  37%  Positivity (%)  0%  *Acceptance rate (%)*  37%  *Patient indicators*  No reports by students that the offer or testing process was stigmatising or undesirable  *Provider indicators*  No reports by staff that the offer or testing process was stigmatising or undesirable | Quality score NA Limited method section | No comments |
| **Drug services** | | | | | | | |
| **HBV** | | | | | | | |
| O'Sullivan, 2016 [40] | *Country*  UK  *Study period*  Dec 2013 - Nov 2017  *Study design* Surveillance/evaluation/audit | Offer of dry blood spot testing (DBST), mobile transient elastography (TE), HCV treatment . | Drug users attending a large substance misuse service in South East England | 391 | Coverage (%)  49% (190/391) (DBST)  Positivity (%) 20% HBcAb+ | *Quality score* NA  No major comments | Denominators not clear for HBV testing |
| Apoola, 2011 [41] | *Country*  UK  *Study period*  Feb 2007 - Dec 2008  *Study design*  RCT | **The intervention (oral swab test) group** had a pre-test discussion for HIV followed by an oral swab test for HIV, HBV and HCV. Results were available in two days.  **The control (blood test) group** had a pre-test discussion for HIV followed by a referral to the same day HIV testing service carried out at the STI clinic. Here they were offered blood tests for HIV, HBV and HCV on the same day. | All young people (under 20 years, male and female) engaged with the young person’s substance misuse service deemed competent to provide informed consent | 27 (intervention) 28 (control) | *Coverage (%)*  92.6% (intervention) 7.4% (control) | *Quality score* acceptable  No major comments | Randomization and blinding could be better; a simple randomisation procedure with opaque envelopes was used |
| **HCV** | | | | | | | |
| Selvapatt, 2017 [42] | *Country*  UK  *Study period*  1 Apr 2012 - 1 Nov 2014  *Study design* Surveillance/evaluation/audit | A HCV screening and treatment outreach programme offering BBV screening using dry blood spot (DBS) testing, implemented within a DTU. | All persons attending the North Westminster Drug and Alcohol Service (NWDAS), a Central London Drug Treatment Unit (DTU). | 321 | *Coverage (%)*  67.3% (216/321)  *Positivity (%)*  41%  31% (viraemic) | *Quality score* NA  Data collection methods were not clear. | Data on costs/cost-effectiveness available |
| O'Sullivan, 2016 [40] | *Country*  UK  *Study period*  Dec 2013 - Nov 2017  *Study design* Surveillance/evaluation/audit | Offer of dry blood spot testing (DBST), mobile transient elastography (TE), HCV treatment . | Drug users attending a large substance misuse service in South East England | 391 | *Coverage (%)*  49% (190/391) (DBST)  *Positivity (%)*  0,53% | *Quality score* NA  No major comments | Denominators not clear for HCV testing |
| Tait, 2013 [21] | *Country*  UK  *Study period*  2009-2011  *Study design* Surveillance/evaluation/audit | DBS testing offered to all individuals who accessed needle exchange (NEXC) or drug treatment services (DTS) | Drug users living in Tayside who accessed needle exchange (NEXC) or drug treatment services (DTS) | Tested:  1123 total 631 NEXC 492 DTC | *Coverage (%)*  84.2% 18.7% (follow-up test within 1 y after the first)  *Positivity (%)*  31.2% total 27.5% NEXC 35.3% DTC  *Newly diagnosed (%)*  22.6% total 22.4% NEXC 22.8% DTC | *Quality score* NA  No major comments | No comments |
| Apoola, 2011 [41] | *Country*  UK  *Study period*  Feb 2007 - Dec 2008  *Study design*  RCT | **The intervention (oral swab test) group** had a pre-test discussion for HIV followed by an oral swab test for HIV, HBV and HCV in the community setting. Results were available in two days.  **The control (blood test) group** had a pre-test discussion for HIV followed by a referral to the same day HIV testing service carried out at the STI clinic. Here they were offered blood tests for HIV, HBV and HCV on the same day. | All young people (under 20 years, male and female) engaged with the young person’s substance misuse service deemed competent to provide informed consent | 27 (intervention) 27 (control) | *Coverage (%)*  100% (intervention) 7.4% (control) | *Quality score* acceptable  No major comments | Randomization and blinding could be better; a simple randomisation procedure with opaque envelopes was used |
| McLeod, 2014 [6] | *Country*  UK  *Study period*  Jan 1999 - Dec 2011  *Study design*  Pre-post study | Surveillance study on HCV testing examining impact of the Hepatitis C Action Plan consisting of awareness-raising activities, for GPs and those at risk, and the introduction of DBS sampling in community drug services to overcome barriers to testing. | Data on anti-HCV tests provided by the West of Scotland Specialist Virology Centre; East of Scotland Specialist Virology Centre; Department of Medical Microbiology at Aberdeen Royal Infirmary and Ninewells Hospital & Medical School. Positive results were laboratory confirmed. | 5,399 (total) 67 (pre-Action Plan) 973 (Action Plan) | *Positivity (%)*  9% (total) 10.5% (pre-Action Plan) 8.3% (Action Plan) | *Quality score* NA  No major comments | RR also reported for initial trend, level change in number tested and change in trend over each setting |
| Roux, 2016 [43] | *Country*  France  *Study period*  2011 - 2013  *Study design*  Non-randomised trial | A national, clustered, multi-site intervention study ANRS-AERLI, providing community-based educational training and education about injection, organized as a series of participant-centered face-to-face educational sessions at harm-reduction centres **(intervention),** compared with centres with no educational sessions (**control).** All participants were interviewed on three occasions: enrolment, 6 and 12 months. | PWID who spontaneously asked for help or information related to injection and who could be reached by phones attending 17 low-threshold drug user harm reduction (HR) centers in France (8 intervention 9 control) | Baseline 88 (intervention) 114 (control)  12 mo 27 (intervention) 54 (control) | *Coverage (%)*  Intervention (baseline; 12 mo) 44%; 85% testing during previous 6 mo   Control group (baseline; 12 mo) 51%; 78% testing during previous 6 mo  *Positivity (%)*  Self-reported HCV seropositivity 15% (intervention) 14% (control) Newly diagnosed (%) | *Quality score* acceptable  No major comments | Participants received a monetary incentive for each telephone interview |
| Hope, 2012 [44] | *Country*  UK  *Study period*  2000 - 2008  *Study design* Surveillance/evaluation/audit | Annual voluntary unlinked-anonymous survey examining the impact of the HCV Action Plan, launched in 2004; a national framework of activities to improve prevention, diagnosis and treatment of HCV. The survey consisted of a HCV test in oral fluid and measured self-reports of uptake of voluntary confidential testing (VCT) for HCV. | Recent initiates to injecting drug use (i.e. had first injected in the preceding 3 years) who participated in the survey, attending 60 drug agencies (both statutory and nonstatutory providers of advice, harm reduction, or treatment services) throughout England. | 3,463 participants of the survey between 2000-2008 | *Coverage (%)*  42% (reported having ever had a VCT for HCV)  *Positivity (%)*  18% | *Quality score* NA  No major comments | Self-reported uptake |
| Craine, 2009 [45] | *Country*  UK  *Study period*  1 May 2007 - 30 Apr 2008 (DBS testing)  1 May 2006 - 30 Apr 2007 (BBV testing)  *Study design*  Pre-post study | A clinical audit of the uptake of DBS testing in a single SMS in the first year of its introduction as a standard diagnostic testing option compared with BBV testing of SMS clients by venepuncture in the previous year. | All clients of the substance misuse service (SMS) national healthcare clinic with a history of injecting drug use |  | 35 tested by venepuncture (2006-2007)  24 tested by venepuncture (2007-2008)  202 tested by DBS (2007-2008) | *Quality score* NA  No major comments | No comments |
| **Online** | | | | | | | |
| **HBV** | | | | | | | |
| Williams, 2017 [46] | *Country*  UK  *Study period*  Sep 2015 – Mar 2016  *Study design*  Surveillance/evaluation/audit | Pilot home-sampling service, accessible via an online service, allowing access to screening for sexually transmitted infections (STIs). Overall acceptability was assessed via online feedback survey and in-depth interviews with service users. |  |  | *Coverage (%)*  4305 kits requested  48% of the test returned (15% providing insufficient blood samples)  *Positivity rate (%)*  0.2%  *Patient indicators*  39% reported difficulties taking blood samples.  95% said they would use the online service again and 93% would recommend it to family and friends.  There was no significant reduction in asymptomatic attendances to STI clinics since introduction of the online service. | *Quality score* NA  No major comments | No comments |
| van der Veen, 2014 [47] | *Country*  Netherlands  *Study period*  September 2010  *Study design*  RCT | A clustered randomized three-group pre and post-test design with three online interventions designed to promote HBV screening in first generation Turkish migrants. One intervention offered **behavioural tailoring (BT)**, and the other intervention offered **behavioural tailoring plus cultural tailoring (BCT)**. Both interventions were based on computer tailoring (a strategy that targets a specific person based on characteristics that are unique to that person). The last intervention group received **generic information (GI)** about HBV, HBV prevention and HBV treatment (this information was the same as that offered in the knowledge modules in the BT and BCT interventions). Subsequently, free HBV screening was offered | Rotterdam-registered people who were born in Turkey and who were 16–40 years old on 1 September 2010. Migrants who had been tested (and were sure that they were an HBV carrier or were immune to HBV) or if they had been vaccinated and had a sufficient vaccine response were excluded. | 10069 invited 1512 logged in via website  1400 eligible for screening  496 (GI) 432 (BT) 472 (BCT) | *Coverage (%)*  44.5 (overall) 43.9% BCT (OR 0.94 95% CI 0.69-1.26) 43.5% BT (OR 0.88 95% CI 0.65-1.19) 46.0% GI | *Quality score* acceptable  No major comments | - the low response rate (15%) may have caused selection bias, as participants were likely more motivated regarding HBV testing than nonparticipants, and this may have influenced their response to the randomized interventions. - Due to unanticipated political sensitivity regarding culturally specific health promotion activities in the target population, the programme organization was severely limited in seeking public attention for this programme. |
| Ruutel, 2015 [48] | *Country*  Estonia  *Study period*  Apr 2013 - Sept 2013  *Study design* Surveillance/evaluation/audit | An Internet-based recruitment system for HIV and STI screening using a questionnaire. All the participants were offered voluntary, anonymous and free-of-charge HIV and STI testing using a special web-based testing service called Testikodus (‘Test at Home’ in English). | Participants were self-identifying as male; living in Estonia; age of 18 years or older; and being sexually attracted to men and/or having ever had sex with a man. Testing took place at the QM laboratory in six sites in larger cities across Estonia. | 265 | *Coverage (%)*  16.2% (43/265)  *Positivity (%)*  0% | *Quality score* NA  No major comments | No comments |
| **HCV** | | | | | | | |
| Ruutel, 2015 [48] | *Country*  Estonia  *Study period*  Apr 2013 - Sept 2013  *Study design* Surveillance/evaluation/audit | An Internet-based recruitment system for HIV and STI screening using a questionnaire. All the participants were offered voluntary, anonymous and free-of-charge HIV and STI testing using a special web-based testing service called Testikodus (‘Test at Home’ in English). | Participants were self-identifying as male; living in Estonia; age of 18 years or older; and being sexually attracted to men and/or having ever had sex with a man. Testing took place at the QM laboratory in six sites in larger cities across Estonia. | 265 | *Coverage (%)*  16.2% (43/265)  *Positivity (%)*  4.6% | *Quality score* NA  No major comments | No comments |
| Zuure, 2011 [49] | *Country*  Netherlands  *Study period*  April 2007 - Dec 2008  *Study design* Surveillance/evaluation/audit | A pilot project combining a mass media information campaign on HCV in the general population with an online risk-assessment tool and free blood-testing procedure for HCV. Individuals who reported at least one risk factor for HCV were advised to have a blood test. | Inhabitants of Amsterdam and South Limburg in the Netherlands, who were at risk for HCV. Individuals already diagnosed with HCV infection were excluded. | 9653 eligible website visitors who completed questionnaire | *Offered (%)*  15.3%  *Coverage (%)*  4.4%  *Positivity (%)*  4.5% 2.8% chronic infection  *Acceptance rate (%)*  28.4% | *Quality score* NA  No major comments | The reach of the media campaign was not measured. It is unknown whether participants at risk who were not tested through the project decided to be tested elsewhere. |
| **Outreach** | | | | | | | |
| **HBV** | | | | | | | |
| Foucher, 2009 [50] | *Country*  France  *Study period*  Jan 2006 - Jan 2007  *Study design* Surveillance/evaluation/audit | Street-based outreach offering non-invasive fibroscan, counseling and testing for HCV and HBV. | All consecutive drug users (>18 years) in two street-based outreaches | 298 | *Offered (%)*  100%  *Coverage (%)*  76.2%  *Positivity (%)*  0%  *Acceptance rate (%)*  97.3% (agreed to the principle) | *Quality score* NA  No major comments | No comments |
| Story, 2016 [51] | *Country*  UK  *Study period*  March 2011-June 2013  *Study design* Surveillance/evaluation/audit | NHS mobile screening service | Homeless attending an NHS mobile screening service |  | *Coverage*  491 tested  *Positivity (%)*  12.4% past or current infection | *Quality score* NA  No major comments | No comments |
| Coenen, 2016 [52] | *Country*  Netherlands  *Study period*  2009 - 2013  *Study design* Surveillance/evaluation/audit | Five large-scale Dutch independent outreach screening programmes that offered targeted HBV screening to first-generation Chinese migrants aiming at secondary prevention. The campaigns combined disease awareness activities with free HBV testing. Key Chinese organisations and figures were engaged in the campaign. The campaign started on Chinese new year, and information was printed in Chinese newspapers, folders and a special website. In Utrecht, Arnhem and Eindhoven, registered Chinese were contacted by letter | First-generation Chinese migrants from Rotterdam (2009), The Hague (2010), Utrecht (2011), Arnhem/Nijmegen region (2013) and Eindhoven (2013). In the screening phase participants were offered free HBV testing at community outreach locations (e.g. Chinese schools and churches). | 29000 | *Coverage (%)*  ≈ 15.3%: 4423 of approximately 29,000 (the Chinese population living in Rotterdam, the Hague, Utrecht, Arnhem/Nijmegen and Eindhoven)  *Positivity (%)*  6.0%  *Newly diagnosed (%)*  2.9% | *Quality score* NA  Data collection methods were not clear. | The actual population might be higher because a large number of Chinese migrants are not officially registered. |
| Zuure, 2013 [53] | *Country*  Netherlands  *Study period*  2009-2010  *Study design* Surveillance/evaluation/audit | Community-based HBV/HCV free-of-cost screening programme; combining education and screening sessions, promoted by key community figures with flyers | Egyptian first generation migrants (18 years and older) recruited/tested at a Coptic church, two mosques, an Egyptian women's empowerment organisation, an Egyptian trade organisation, a weekend school for Islamic Egyptians and an Egyptian supermarket | +- 1500 flyers sent to community organisations for distribution | *Coverage (%)*  31% (465/1500)  *Positivity (%)*  1.1%  16.8 % Anti-HBc+  *Newly diagnosed (%)*  0.6% | *Quality score* NA  Denominator is not appropriate | Denominator is the approximate number of flyers sent for distribution, it is not known how many individuals received flyers, so Coverage may be underestimated |
| Richter, 2012 [54] | *Country*  Netherlands  *Study period*  NR  *Study design* Surveillance/evaluation/audit | Culturally-targeted screening project involving campaigns with posters, brochures, newspaper and radio segments, a documentary video and website. Educational meetings concerning hepatitis were organized, with all participants being offered a blood screening test by a mobile laboratory team. | Turkish migrants living in the Turkish community of Arnhem, the Netherlands and attending community centres, mosques and/or the GP | 6337 total Turkish population of Arnhem | *Coverage (%)*  11.2% 10.2% tested with complete data set  *Positivity (%)*  2.78% (HBsAg) 3.87% (anti-HBc) 0% (HBeAg)  *Patient indicators*  Considered educational meeting good and understandable: 97%  Willing to pass on the new knowledge to family members and friends:48%  Willing to distribute brochures: 14%  Orally, they reported back that they were very satisfied regarding the information, the organization of the project, and that they felt they were taken seriously | *Quality score* NA  Denominator not clear | - Results unclearly written, denominator in each case was not clear.  - Positivity rate was higher than that reported from Arnhem hospital records - The testing offer was taken up by around 10% of the population, but it is not reported how many were aware of the testing offer and what the uptake was among these. |
| Veldhuijzen, 2012 [55] | *Country*  Netherlands  *Study period*  2009  *Study design* Surveillance/evaluation/audit | A campaign combining disease awareness activities with free testing at outreach locations | Chinese migrants (first and second generation) living in Rotterdam |  | *Coverage*  1090 tested  *Positivity (%)*  8.5% Chronic HBV (anti-HBc+/HBsAg+) 41% Previous HBV (anti-HBc+/HBsAg-) | *Quality score* NA  Sample may not be representative of study population | - Women and older people were overrepresented among the respondents. People who know they are chronically infected may not take part in screening, however, people with infected family members may be more likely to take part |
| McPherson, 2013 [56] | *Country*  UK  *Study period*  NR  *Study design* Surveillance/evaluation/audit | Targeted case finding: Subjects invited to HBV education and screening sessions held in community centres. HBsAg and HBcAb were tested with dry blood spot tests (DBST). | A ‘high’ prevalence community of North East (NE) England – British–Chinese, as well as an ‘intermediate’ community – the British–South Asian community (Pakistan, India, Bangladesh or Sri Lanka) attending 4 sites in the NE of England (the NCHLC; the True Jesus Church, Shieldfield, Newcastle; the True Jesus Church, Sunderland and The Tees Valley Chinese Community Centre, Middlesbrough) and 5 Mosques in Newcastle and Middlesbrough and the Sunderland Bangladeshi community centre, respectively. | 86,000 (total) 21,000 (British-Chinese) 65,000 (British-South Asian) | *Coverage*  606 tested (British-Chinese) 520 tested (British-South Asian)  *Positivity (%)*  5.5% (of which 16.1% had previous infection) 8% (anti-HBc)  8.7% (British-Chinese) 1.7% (British-South Asian) 13% (anti-HBc British-Chinese) 1.7% (anti-HBc British-South Asian)  *Newly diagnosed (%)*  4.6% cHBV (excluding with known cHBV) 7.2% (British-Chinese) 1.7% (British-South Asian) | *Quality score* NA  Data collection methods were not clear. | No comments |
| Vedio, 2013 [57] | *Country*  UK  *Study period*  Sept 2009-June 2012  *Study design* Surveillance/evaluation/audit | Pilot project of DBS testing in outreach settings frequented by the Chinese community. Testing was advertised in Chinese language newspapers , papers and bulletins and through GPs | Chinese migrants offered DBS testing at the Kinhon Chinese centre, a Chinese church, a Saturday Chinese school and a chinese wholesaler in Sheffield |  | *Coverage*  299 tested  *Positivity (%)*  8.7%  12.2% past infection (HBsAg−/HBcAb+)  *Patient indicators*  Information session was useful: 100%  Able to ask all the questions: 93.7%  Explanations were clear: 96.5%  The test caused any discomfort: 5% | *Quality score* NA  Sample may not be representative of study population | - The sample was a self-selected sample, as it was composed entirely of people requesting or volunteering for testing following prior or on the spot advertisement. |
| Jafferbhoy, 2012 [58] | *Country*  UK  *Study period*  July 2009  *Study design* Surveillance/evaluation/audit | An outreach testing intervention consisting of a series of HCV awareness meetings. Thereafter short-term outreach HCV testing clinics were set up in the same venues. Venous blood samples were obtained and tested for HCV IgG and HbsAg. A short questionnaire was also completed. | Immigrant Pakistani population visiting the three mosques and Pakistani Women’s centre in the city of Dundee, Scotland | 1,723 Pakistani individuals registered in Dundee | *Coverage (%)*  9,8% (170/1723)  *Positivity (%)*  0.6% | Quality score NA  No major comments | - The testing offer was taken up by almost 10% of the population, but it is not reported how many were aware of the testing offer and what the uptake was among these.  - Testing was offered at mosques, which excludes individuals who do not attend and some women who may pray at home. |
| Sahajian, 2011 [59] | *Country*  France  *Study period*  22 Oct 2007 - 22 Apr 2009  *Study design*  RCT | Two **screening strategies (S1 and S2) and non-intervention strategy (S0)** completed by a mobile team. The intervention shelters involved group information sessions followed by an individual consultation (IC) during which subjects were offered systematic taking of blood (for biological blood examinations and screening serology) associated with a medical check-up. S1 shelters referred participants for testing at a health centre and S2 shelters performed on-site testing. | Underprivileged people aged over 18 years old living in 18 shelters in the Lyon area (6 shelters per intervention group). Taking of blood and check-up were either conducted at the Welfare and Health Examination Centres (WHECs) (S1) or at the Lyon area shelters (S2). | Total population: 811 (S0) 1041 (S1) 784 (S2)  Included population (for S1 and S2 these are participants attending group information sessions and accepting taking of blood): 811 (S0) 222 (S1) 243 (S2) | *Coverage (%)*  Coverage in total pop: 1.5% (S0) 9.1% (S1) 18.6% (S2) p<10-6  Coverage in included pop: 1.5% (12/811)(S0) 42.8% (95/222)(S1) 59.7% (145/243)(S2) p <10-6  *Positivity (%)*  HBsAg; anti-HBc  0%; 0% (S0) 2.1%; 67.4% (S1) 4.8%; 70.3%(S2) | *Quality score* acceptable  No major comments | Randomization method and concealment were not described. |
| **HCV** | | | | | | | |
| Foucher, 2009 [50] | *Country*  France  *Study period*  Jan 2006 - Jan 2007  *Study design* Surveillance/evaluation/audit | Street-based outreach offering non-invasive fibroscan, counseling and testing for HCV and HBV. | All consecutive drug users (>18 years) in two street-based outreaches | 298 | *Offered (%)*  100%  *Coverage (%)*  76.2%  *Positivity (%)*  37.6%  *Newly diagnosed (%)*  11%  *Acceptance rate (%)*  97.3% (agreed to the principle) | *Quality score* NA  No major comments | No comments |
| Story, 2016  [51] | *Country*  UK  *Study period*  March 2011-June 2013  *Study design* Surveillance/evaluation/audit | NHS mobile screening service | Homeless people attending an NHS mobile screening service |  | *Coverage*  491 tested  *Positivity rate (%)*  13.0% current infection | *Quality score* NA  No major comments | No comments |
| Zuure, 2013 [53] | *Country*  Netherlands  *Study period*  2009-2010  *Study design* Surveillance/evaluation/audit | Community-based HBV/HCV free-of-cost screening programme; combining education and screening sessions, promoted by key community figures with flyers | Egyptian first generation migrants (18 years and older) recruited/tested at a Coptic church, two mosques, an Egyptian women's empowerment organisation, an Egyptian trade organisation, a weekend school for Islamic Egyptians and an Egyptian supermarket | +- 1500 flyers sent to community organisations for distribution | *Coverage (%)*  31% (465/1500)  *Positivity (%)*  2.4%  2.2% HCV-RNA+  *Newly diagnosed (%)*  1.9% | *Quality score* NA  Denominator is not appropriate | Denominator is the approximate number of flyers sent for distribution, it is not known how many individuals received flyers, so uptake may be underestimated |
| Richter, 2012 [54] | *Country*  Netherlands  *Study period*  NR  *Study design* Surveillance/evaluation/audit | Culturally-targeted screening project involving campaigns with posters, brochures, newspaper and radio segments, a documentary video and website. Educational meetings concerning hepatitis were organized, with all participants being offered a blood screening test by a mobile laboratory team. | Turkish migrants living in the Turkish community of Arnhem, the Netherlands and attending community centres, mosques and/or the GP | 6337 total Turkish population of Arnhem | *Coverage (%)*  11.2% 10.2% tested with complete data set  *Positivity (%)*  0.3%  0.3% (HCV-RNA)  *Patient indicators*  Considered educational meeting good and understandable: 97%  Willing to pass on the new knowledge to family members and friends:48%  Willing to distribute brochures: 14%  Orally, they reported back that they were very satisfied regarding the information, the organization of the project, and that they felt they were taken seriously | *Quality score* NA  Denominator not clear | - Results unclearly written, denominator in each case was not clear.  - Positivity rate was higher than that reported from Arnhem hospital records - The testing offer was taken up by around 10% of the population, but it is not reported how many were aware of the testing offer and what the uptake was among these. |
| Jafferbhoy, 2012 [58] | *Country*  UK  *Study period*  July 2009  *Study design* Surveillance/evaluation/audit | An outreach testing intervention consisting of a series of HCV awareness meetings. Thereafter short-term outreach HCV testing clinics were set up in the same venues. Venous blood samples were obtained and tested for HCV IgG and HbsAg. A short questionnaire was also completed. | Immigrant Pakistani population visiting the three mosques and Pakistani Women’s centre in the city of Dundee, Scotland | 1,723 Pakistani individuals registered in Dundee | *Coverage (%)*  9,8% (170/1723)  *Positivity (%)*  4.1% (95% CI 2%-8%)  2.9% (HCV-RNA) | Quality score NA  No major comments | - The testing offer was taken up by almost 10% of the population, but it is not reported how many were aware of the testing offer and what the uptake was among these.  - Testing was offered at mosques, which excludes individuals who do not attend and some women who may pray at home. |
| McPherson, 2013 [56] | *Country*  UK  *Study period*  NR  *Study design* Surveillance/evaluation/audit | Targeted case finding: Subjects invited to HBV education and screening sessions held in community centres. HBsAg and HBcAb were tested with dry blood spot tests (DBST). | A ‘high’ prevalence community of North East (NE) England – British–Chinese, as well as an ‘intermediate’ community – the British–South Asian community (Pakistan, India, Bangladesh or Sri Lanka) attending 4 sites in the NE of England (the NCHLC; the True Jesus Church, Shieldfield, Newcastle; the True Jesus Church, Sunderland and The Tees Valley Chinese Community Centre, Middlesbrough) and 5 Mosques in Newcastle and Middlesbrough and the Sunderland Bangladeshi community centre, respectively. | 65,000 (British-South Asian) | *Coverage*  520 tested (British-South Asian)  *Positivity (%)*  0.8% | *Quality score* NA  Data collection methods were not clear. | No comments |
| Sahajian, 2011 [59] | *Country*  France  *Study period*  22 Oct 2007 - 22 Apr 2009  *Study design*  RCT | Two **screening strategies (S1 and S2) and non-intervention strategy (S0)** completed by a mobile team. The intervention shelters involved group information sessions followed by an individual consultation (IC) during which subjects were offered systematic taking of blood (for biological blood examinations and screening serology) associated with a medical check-up. S1 shelters referred participants for testing at a health centre and S2 shelters performed on-site testing. | Underprivileged people aged over 18 years old living in 18 shelters in the Lyon area (6 shelters per intervention group). Taking of blood and check-up were either conducted at the Welfare and Health Examination Centres (WHECs) (S1) or at the Lyon area shelters (S2). | Total population: 811 (S0) 1041 (S1) 784 (S2)  Included population (for S1 and S2 these are participants attending group information sessions and accepting taking of blood): 811 (S0) 222 (S1) 243 (S2) | *Coverage (%)*  Coverage in total pop: 1.5% (S0) 9.1% (S1) 18.6% (S2) p<10-6  Coverage in included pop: 1.5% (12/811)(S0) 42.8% (95/222)(S1) 59.7% (145/243)(S2) p <10-6  *Positivity (%)*  0% (S0)  3.2% (S1)  2.8% (S2) | *Quality score* acceptable  No major comments | Randomization method and concealment were not described. |
| **Both HBV/HCV not specified** | | | | | | | |
| Wood, 2014 [60] | *Country*  UK  *Study period*  Apr 2013 - Dec 2013  *Study design* Surveillance/evaluation/audit | A novel outreach STI screening service incorporating nurse-delivered screening at a monthly screening clinic held at the sauna, and self-sampled postal testing kits (DBS fingerprick testing). Health promotion workers were present at the venue three days a week. The service was compared to standard screening at a local sexual health clinic. | Asymptomatic men who have sex with men (MSM) visiting a local sauna in a predominantly rural area | First 30 users of each service:  30 (sauna nurse outreach) 30 (DIY postal kits) 30 (sexual health clinic) | *Coverage (%)*  Ever tested for STI; accepted blood screening 53.3%; 83.3% (sauna nurse outreach) 60%; 53.3% (DIY postal kits) 93.3%; 100% (Sexual health clinic)  *Positivity (%)*  HBV or HCV active; cleared infection 0%; 4% (Sauna nurse outreach) 6.25%; 25% (DIY postal kits) 0%; 0% (Sexual health clinic) Newly diagnosed (%) | *Quality score* NA  Result section is limited and data is not analysed in depth | Outcomes not specified for HBV or HCV |
| **Multiple settings without separate results** | | | | | | | |
| **HCV** | | | | | | | |
| Bishton, 2014 [34] | *Country*  UK  *Study period*  18 months  *Study design* Surveillance/evaluation/audit | DBS testing offered for HCV, HBV and HIV, and vaccination offered for HBV | All who considered themselves at risk and current tier 3 drug treatment clients (including alcohol clients with a previous history of drug use), clients at the tier 2 drug treatment needle exchange, at the needle exchange at the local homeless clinic and at the two pharmacy needle-exchange sites with the highest current usage located in North East Essex. | NR | *Coverage*  266 tested  *Positivity (%)*  35%  53% (of % positive had active infection) | *Quality score* NA  Method section is limited and no structure in the article | Short communication article |
| Fernandez-Lopez, 2016 [61] | *Country*  Spain  *Study period*  Apr 2011 - Dec 2011  *Study design* Surveillance/evaluation/audit | Oral fluid rapid test | PWID who never had a test, had been tested but not sure of the result or had a negative test in the previous 6 months, who visit 13 Harm Reduction Programmes (HRP) in Catalonia, Spain (Six HRP were classified as facility-based centres, five as mobile units or street outreach teams and two HRP were classified as mixed HRP) | 240 | *Offered (%)*  73%  *Coverage (%)*  72% (172/240)  *Positivity (%)*  20.3% (overall) 11.3% (13/115 facility-based center) 44.8% (13/29 mobile unit) 32.1% (9/28 mixed)  *Acceptance rate (%)*  98.2%  *Provider indicators*  Both oral swab testing and test interpretation were either easy or very easy: 100% . Expressing full confidence in the results: 60%; Partially confident of the test results: 40% | *Quality score* NA  Data collection methods were not clear. | No comments |

##### Table 11. Evidence base for the effectiveness of testing initiatives in multiple settings

| **Reference** | **Study characteristics** | **Testing approach** | **Study population and setting** | **Sample N** | **Outcomes** | **Critical appraisal** | **General comments** |
| --- | --- | --- | --- | --- | --- | --- | --- |
| **HCV** | | | | | | | |
| Delarocque-Astagneau, 2010 [62] | *Country*  France  *Study period*  Sero-prevalence surveys: 1994 and 2004  Rena-VHC and Hepatology reference centres surveillance networks: 2000-2006  *Study design*  Pre-post study | Before-after study to look at impact of national HCV prevention programme (The national institute for surveillance [Institut de Veille Sanitaire (InVS)]) | Individuals tested as part of a seroprevalence survey, eligible for a medical check-up in a Social Security Medical Centre (SSMC) in four regions of France (1994) and of five Primary Health Insurance Units linked to a SSMC (2004). In addition all blood samples tested for HCV in on of the 281 laboratories participating in the Rena-VHC surveillance network and newly referred HCV+ patients attending 26 hepatology reference centres which were part of the Hepatology reference centrer surveillance network. |  | *Coverage*  Before: 301,998 tests After: 437,524 (2005) After: 394,882 (2006)  *Positivity (%)*  4.3% (2000) 2.9% (2006) 1.3% (2000, confirmed positive) 0.6% (2006, confirmed positive) | *Quality score* NA  No major comments | No comments |
| McLeod, 2014 [6] | *Country*  UK  *Study period*  Jan 1999 - Dec 2011  *Study design*  Pre-post study | Surveillance study on HCV testing examining impact of the Hepatitis C Action Plan consisting of awareness-raising activities, for GPs and those at risk, and the introduction of DBS sampling in community drug services to overcome barriers to testing. | Data on anti-HCV tests provided by the West of Scotland Specialist Virology Centre; East of Scotland Specialist Virology Centre; Department of Medical Microbiology at Aberdeen Royal Infirmary and Ninewells Hospital & Medical School. Positive results were laboratory confirmed. | 297,689 (total) 19,058 (pre-Action Plan) 29,045 (Action Plan) | *Positivity (%)*  8.1% (total) 9.6% (pre-Action Plan) 6.8% (Action Plan) | *Quality score* NA  No major comments | RR also reported for initial trend, level change in number tested and change in trend over each setting |
| McLeod, 2014 [6] | *Country*  UK  *Study period*  Jan 1999 - Dec 2011  *Study design*  Pre-post study | Surveillance study on HCV testing examining impact of the Hepatitis C Action Plan consisting of awareness-raising activities, for GPs and those at risk, and the introduction of DBS sampling in community drug services to overcome barriers to testing. | Data on anti-HCV tests provided by the West of Scotland Specialist Virology Centre; East of Scotland Specialist Virology Centre; Department of Medical Microbiology at Aberdeen Royal Infirmary and Ninewells Hospital & Medical School. Positive results were laboratory confirmed. | 15,319 (total) 1,079 (pre-Action Plan) 1,338 (Action Plan) | *Coverage*  67 tested pre-action plan 973 tested during action plan  *Positivity (%)*  36.3% (total) 19.4% (pre-Action Plan) 38.1% (Action Plan) | *Quality score* NA  No major comments | RR also reported for initial trend, level change in number tested and change in trend over each setting |

1. Hargreaves, S., et al., *Screening for latent TB, HIV, and hepatitis B/C in new migrants in a high prevalence area of London, UK: a cross-sectional study.* BMC Infect Dis, 2014. **14**: p. 657.

2. Roudot-Thoraval, F., et al., *Successful management of precarious population after systematic HBV testing in france: A prospective cohort study.* Journal of Hepatology, 2015. **62**: p. S825-S826.

3. Anderson, E.M., et al., *Evaluation of a general practice based hepatitis C virus screening intervention.* Scott Med J, 2009. **54**(3): p. 3-7.

4. Parisi, M.R., et al., *Point-of-care testing for HCV infection: recent advances and implications for alternative screening.* New Microbiol, 2014. **37**(4): p. 449-57.

5. Helsper, C.W., et al., *A support programme for primary care leads to substantial improvements in the effectiveness of a public hepatitis C campaign.* Fam Pract, 2010. **27**(3): p. 328-32.

6. McLeod, A., et al., *Rise in testing and diagnosis associated with Scotland's Action Plan on Hepatitis C and introduction of dried blood spot testing.* J Epidemiol Community Health, 2014. **68**(12): p. 1182-8.

7. Lambert, J.S., et al., *The Dublin hepcheck study: Community based testing of HCV by point of care oraquick® HCV saliva test in homeless populations.* Journal of Hepatology, 2016. **64**(2): p. S726.

8. Cullen, B.L., et al., *Identifying former injecting drug users infected with hepatitis C: an evaluation of a general practice-based case-finding intervention.* J Public Health (Oxf), 2012. **34**(1): p. 14-23.

9. Kunkel, J., et al., *Screening for viral hepatitis among migrants in the EU: What lessons can we learn from poor response to a pilot trial using GP registers?* Journal of Hepatology, 2015. **62**: p. S838.

10. O'Connell, S., et al., *Hepatitis C diagnosis and linkage to care rates in an urban emergency department blood borne virus screening programme.* Journal of Hepatology, 2016. **64**(2): p. S525.

11. Orkin, C., et al., *Incorporating HIV/hepatitis B virus/hepatitis C virus combined testing into routine blood tests in nine UK Emergency Departments: The "Going Viral" campaign.* HIV Medicine, 2016. **17**(3): p. 222-230.

12. Aparicio, C., et al., *[Proposal of HIV, HBV and HCV targeted screening: short period feasibility study in a free-access outpatient medical structure].* Presse Med, 2012. **41**(10): p. e517-23.

13. Richter, C., et al., *Screening for chronic hepatitis B and C in migrants from Afghanistan, Iran, Iraq, the former Soviet Republics, and Vietnam in the Arnhem region, The Netherlands.* Epidemiol Infect, 2014. **142**(10): p. 2140-6.

14. Sanger, C., et al., *Acceptability and necessity of HIV and other blood-borne virus testing in a psychiatric setting.* Br J Psychiatry, 2013. **202**(4): p. 307-8.

15. Keel, P., et al., *Assessing the impact of a nurse-delivered home dried blood spot service on uptake of testing for household contacts of hepatitis B-infected pregnant women across two London trusts.* Epidemiol Infect, 2016. **144**(10): p. 2087-97.

16. Diab-Elschahawi, M., et al., *Evaluation of a universal vs a targeted hepatitis C virus screening strategy among pregnant women at the Vienna University Hospital.* Am J Infect Control, 2013. **41**(5): p. 459-60.

17. Pauti, M.D., N. Simonnot, and P. Estecahandy, *[Development of actions for the prevention of HIV, hepatitis and sexually transmitted infections among immigrants consulting in the doctors of the world "Missions France"].* Med Mal Infect, 2009. **39**(3): p. 191-5.

18. Schreuder, I., et al., *Seroprevalence of HIV, hepatitis b, and hepatitis c among opioid drug users on methadone treatment in the netherlands.* Harm Reduction Journal, 2010. **7**.

19. Arain, A., et al., *Pilot Study: Combining Formal and Peer Education with FibroScan to Increase HCV Screening and Treatment in Persons who use Drugs.* J Subst Abuse Treat, 2016. **67**: p. 44-9.

20. Lindenburg, C.E., et al., *Hepatitis C testing and treatment among active drug users in Amsterdam: results from the DUTCH-C project.* Eur J Gastroenterol Hepatol, 2011. **23**(1): p. 23-31.

21. Tait, J.M., et al., *Dry blood spot testing for hepatitis C in people who injected drugs: Reaching the populations other tests cannot reach.* Frontline Gastroenterology, 2013. **4**(4): p. 255-262.

22. Bottero, J., et al., *Simultaneous Human Immunodeficiency Virus-Hepatitis B-Hepatitis C Point-of-Care Tests Improve Outcomes in Linkage-to-Care: Results of a Randomized Control Trial in Persons Without Healthcare Coverage.* Open Forum Infect Dis, 2015. **2**(4): p. ofv162.

23. Nosotti, L., et al., *HBV infection prevalence and vaccination in an immigrant population in Rome.* Digestive and Liver Disease, 2016. **48**: p. e130.

24. El-Hamad, I., et al., *Point-of-care screening, prevalence, and risk factors for hepatitis B infection among 3,728 mainly undocumented migrants from non-EU countries in northern Italy.* J Travel Med, 2015. **22**(2): p. 78-86.

25. Radley, A., et al., *A quasi-experimental evaluation of dried blood spot testing through community pharmacies in the Tayside region of Scotland.* Frontline Gastroenterology, 2017. **8**(3): p. 221-228.

26. Radley, A., J. Tait, and J.F. Dillon, *DOT-C: A cluster randomised feasibility trial evaluating directly observed anti-HCV therapy in a population receiving opioid substitute therapy from community pharmacy.* Int J Drug Policy, 2017.

27. Patel, S., B. Clarke, and G. Bird, *Hepatitis B and hepatitis c virus case finding in a medium security UK prison.* Canadian Journal of Gastroenterology and Hepatology, 2016. **2016**.

28. Sagnelli, E., et al., *Blood born viral infections, sexually transmitted diseases and latent tuberculosis in italian prisons: a preliminary report of a large multicenter study.* European review for medical and pharmacological sciences, 2012. **16**(15): p. 2142-2146.

29. Craine, N., et al., *A stepped wedge cluster randomized control trial of dried blood spot testing to improve the uptake of hepatitis C antibody testing within UK prisons.* Eur J Public Health, 2015. **25**(2): p. 351-7.

30. McAllister, G., et al., *Uptake of hepatitis C specialist services and treatment following diagnosis by dried blood spot in Scotland.* J Clin Virol, 2014. **61**(3): p. 359-64.

31. Richens, J., et al., *A randomised controlled trial of computer-assisted interviewing in sexual health clinics.* Sex Transm Infect, 2010. **86**(4): p. 310-4.

32. Murira, J. and E. Monteiro, *Hepatitis C screening by country of birth in a genitourinary medicine clinic-how much are we missing?* Sexually Transmitted Infections, 2016. **92**: p. A21.

33. Scott, C., et al., *Unselected hepatitis C screening of men who have sex with men attending sexual health clinics.* J Infect, 2010. **60**(5): p. 351-3.

34. Bishton, E., et al., *Screening for Hepatitis C in injecting and ex-injecting drug users in North East Essex.* Public Health, 2014. **128**(11): p. 1036-1038.

35. Defossez, G., et al., *Evaluation of the French national plan to promote screening and early management of viral hepatitis C, between 1997 and 2003: a comparative cross-sectional study in Poitou-Charentes region.* Eur J Gastroenterol Hepatol, 2008. **20**(5): p. 367-72.

36. Hickman, M., et al., *Increasing the uptake of hepatitis C virus testing among injecting drug users in specialist drug treatment and prison settings by using dried blood spots for diagnostic testing: a cluster randomized controlled trial.* J Viral Hepat, 2008. **15**(4): p. 250-4.

37. Tafuri, S., et al., *Prevalence of Hepatitis B, C, HIV and syphilis markers among refugees in Bari, Italy.* BMC infectious diseases, 2010. **10**(1): p. 213.

38. Okpo, E., H. Corrigan, and P. Gillies, *Blood borne virus (BBV) testing in a university setting in North-East Scotland: a pilot initiative.* Public Health, 2015. **129**(6): p. 825-7.

39. Selvapatt, N., L. Harrison, and A. Brown, *A pilot study of outreach testing for hepatitis C and linkage to care in a London centre for homeless persons.* Gut, 2015. **64**: p. A109.

40. O'Sullivan, M., et al., *Project ITTREAT (integrated community based test-stage-treat) HCV service for people who inject drugs (PWID).* Hepatology, 2016. **63**(1): p. 385A.

41. Apoola, A. and L. Brunt, *A randomised controlled study of mouth swab testing versus same day blood tests for HIV infection in young people attending a community drug service.* Drug Alcohol Rev, 2011. **30**(1): p. 101-3.

42. Selvapatt, N., et al., *The cost impact of outreach testing and treatment for hepatitis C in an urban Drug Treatment Unit.* Liver Int, 2017. **37**(3): p. 345-353.

43. Roux, P., et al., *Increased Uptake of HCV Testing through a Community-Based Educational Intervention in Difficult-to-Reach People Who Inject Drugs: Results from the ANRS-AERLI Study.* PLoS ONE, 2016. **11**(6).

44. Hope, V., et al., *Hepatitis C infection among recent initiates to injecting in England 2000-2008: Is a national hepatitis C action plan making a difference?* J Viral Hepat, 2012. **19**(1): p. 55-64.

45. Craine, N., et al., *Improving blood-borne viral diagnosis; clinical audit of the uptake of dried blood spot testing offered by a substance misuse service.* J Viral Hepat, 2009. **16**(3): p. 219-22.

46. Williams, S., et al., *Acceptability, uptake and impact of online home-sampling for stis in Hampshire, UK: A service evaluation.* Sexually Transmitted Infections, 2017. **93**: p. A6.

47. van der Veen, Y.J., et al., *Cultural tailoring to promote hepatitis B screening in Turkish Dutch: a randomized control study.* Health Promot Int, 2014. **29**(4): p. 692-704.

48. Ruutel, K., L. Lohmus, and J. Janes, *Internet-based recruitment system for HIV and STI screening for men who have sex with men in Estonia, 2013: analysis of preliminary outcomes.* Euro Surveill, 2015. **20**(15).

49. Zuure, F.R., et al., *Using mass media and the internet as tools to diagnose hepatitis Cinfections in the general population.* American Journal of Preventive Medicine, 2011. **40**(3): p. 345-352.

50. Foucher, J., et al., *FibroScan used in street-based outreach for drug users is useful for hepatitis C virus screening and management: a prospective study.* J Viral Hepat, 2009. **16**(2): p. 121-31.

51. Story, A., A. Hayward, and R. Aldridge, *Co-infection with hepatitis C, hepatitis B, HIV and latent TB infection among homeless people in London.* Journal of Hepatology, 2016. **64**(2): p. S455-S456.

52. Coenen, S., et al., *Clinical impact of five large-scale screening projects for chronic hepatitis B in Chinese migrants in the Netherlands.* Liver Int, 2016. **36**(10): p. 1425-32.

53. Zuure, F.R., et al., *Screening for hepatitis B and C in first-generation Egyptian migrants living in the Netherlands.* Liver International, 2013. **33**(5): p. 727-738.

54. Richter, C., et al., *Hepatitis B prevalence in the Turkish population of Arnhem: implications for national screening policy?* Epidemiol Infect, 2012. **140**(4): p. 724-30.

55. Veldhuijzen, I.K., et al., *Identification and treatment of chronic hepatitis B in Chinese migrants: Results of a project offering on-site testing in Rotterdam, the Netherlands.* Journal of Hepatology, 2012. **57**(6): p. 1171-1176.

56. McPherson, S., et al., *Targeted case finding for hepatitis B using dry blood spot testing in the British-Chinese and South Asian populations of the North-East of England.* J Viral Hepat, 2013. **20**(9): p. 638-44.

57. Vedio, A.B., et al., *Hepatitis B: report of prevalence and access to healthcare among Chinese residents in Sheffield UK.* J Infect Public Health, 2013. **6**(6): p. 448-55.

58. Jafferbhoy, H., et al., *The effectiveness of outreach testing for hepatitis C in an immigrant Pakistani population.* Epidemiol Infect, 2012. **140**(6): p. 1048-53.

59. Sahajian, F., et al., *A randomized trial of viral hepatitis prevention among underprivileged people in the Lyon area of France.* J Public Health (Oxf), 2011. **33**(2): p. 182-92.

60. Wood, M., R. Elks, and M. Grobicki, *Outreach sexual infection screening and postal tests in men who have sex with men: How do they compare with clinicbased screening?* HIV Medicine, 2014. **15**: p. 32.

61. Fernandez-Lopez, L., et al., *Implementation of rapid HIV and HCV testing within harm reduction programmes for people who inject drugs: a pilot study.* AIDS Care, 2016. **28**(6): p. 712-6.

62. Delarocque-Astagneau, E., et al., *The impact of the prevention programme of hepatitis C over more than a decade: the French experience.* J Viral Hepat, 2010. **17**(6): p. 435-43.
